# Participation of Women in Cardiovascular Trials From 2017 to 2023: A Systematic Review

**DOI:** 10.1001/jamanetworkopen.2025.29104

**Published:** 2025-08-31

**Authors:** Frederick Berro Rivera, John Vincent Magalong, Nathan Ross B. Bantayan, Nicole Tesoro, Mark Jason Milan, Vikramjit Purewal, Polyn Luz S. Pine, Chieh-Mei Tsai, Ann Marie Navar, Sharon L. Mulvagh, James Januzzi, C. Michael Gibson, Anuradha Lala, Susan Cheng, Kyla Lara-Breitinger, Mayra Guerrero, Martha Gulati

**Affiliations:** 1 Division of Cardiology, Mayo Clinic, Rochester, Minnesota; 2 College of Medicine, San Beda University, Manila, Philippines; 3 University of the Philippines College of Medicine, Manila, Philippines; 4 Department of Medicine, NYC Health + Hospitals/South Brooklyn Health, New York, New York; 5 Department of Medicine, HonorHealth Mountain Vista Medical Center, Mesa, Arizona; 6 Ateneo School of Medicine and Public Health, Manila, Philippines; 7 Division of Cardiology, Department of Internal Medicine, University of Texas Southwestern Medical Center, Dallas; 8 Women’s Heart Health Clinic, Queen Elizabeth II Health Sciences Centre, Halifax, Canada; 9 Division of Cardiology, Department of Medicine, Massachusetts General Hospital, Harvard Medical School, Boston; 10 Baim Institute for Clinical Research, Boston, Massachusetts; 11 Boston Clinical Research Institute, Boston, Massachusetts; 12 Samuel Bronfman Department of Medicine (Cardiology), The Lauder Family Cardiovascular Center of Mount Sinai Heart, New York, New York; 13 Department of Cardiology, Barbra Streisand Women’s Heart Center, Cedars-Sinai Smidt Heart Institute, Los Angeles, California

## Abstract

**Importance:**

Cardiovascular (CV) disease is the leading cause of death globally for both men and women, yet women remain historically underrepresented in CV clinical trials, despite facing a disproportionately high burden of morbidity and mortality in many forms of CV disease.

**Objective:**

To determine the representation of women across a broad range of CV trials.

**Evidence Review:**

The participation of women in CV trials registered on ClinicalTrials.gov from 2017 to 2023 was systematically determined through the extraction of publicly available information. Data were extracted to identify the country of study, disease type, trial size, clinical intervention, and age of the participants. The proportion of women and the ratio of number of female to male participants (F:M ratio) were calculated for each trial. The women’s participation:prevalence ratio (PPR) was estimated for each trial based on the relative prevalence of the disease by sex in the specified region.

**Findings:**

A total of 1079 registered CV trials were identified, including 1 396 104 participants, of whom 571 641 (41.0%) were women. The F:M ratio was significantly lower for studies on arrhythmia (median [IQR], 0.5), coronary heart disease (median [IQR], 0.39 [0.33-0.70]), acute coronary syndrome (median [IQR], 0.32 [0.24-0.51]), and heart failure (median [IQR], 0.51 [0.32-0.87]) but higher for obesity (median [IQR], 1.44 [1.08-4.50]) and pulmonary hypertension (median [IQR], 2.86 [1.50-3.97]) trials. The F:M ratio was higher for trials on lifestyle interventions (median [IQR], 1.51 [0.77-3.24]) than for drug trials. PPRs were low for clinical trials on coronary heart disease (median [IQR], 0.66 [0.50-0.86]), acute coronary syndrome (median [IQR], 0.79 [0.51-0.87]), and stroke (median [IQR], 0.74 [0.61-0.95]). Representation of women in CV trials varied by disease state, region, intervention, and sponsor type.

**Conclusions and Relevance:**

These findings highlight both progress and persistent challenges in representation of women within CV trials. These gaps not only limit the generalizability of trial outcomes but also perpetuate inequities in evidence-based care for women with CV conditions.

## Key Points

**Question:**

What are the trends of participation of women in cardiovascular trials from 2017 to 2023?

**Findings:**

This systematic review of 1079 cardiovascular clinical trials registered from 2017 to 2023 found that median female:male ratios were significantly lower for trials on arrhythmia, coronary heart disease, acute coronary syndrome, and heart failure and participation:prevalence ratios were low for trials on coronary heart disease, acute coronary syndrome, and stroke. Representation of women in cardiovascular trials varied by disease state, region, intervention, and sponsor type.

**Meaning:**

These findings suggest that targeted and sustained efforts, including the implementation of inclusive trial designs, enhanced outreach strategies, and regulatory policies mandating diversity, are needed to bridge this disparity.

## Teaser

This systematic review describes the representation of women across a broad range of cardiovascular trials registered from 2017 to 2023.

## Introduction

Cardiovascular (CV) diseases are the leading cause of mortality globally, affecting both men and women.^1^ Well-documented sex-specific differences in disease manifestation, progression, and treatment response highlight the critical need for representation of women in CV clinical trials in proportion to the sex distribution of disease prevalence.^2,3,4,5,6,7,8,9,10,11,12^ Yet, women remain underrepresented, limiting the generalizability of trial findings and contributing to potential disparities in clinical outcomes.^13,14^ Prior evaluations of women’s underrepresentation have often focused narrowly on conditions, such as acute coronary syndromes (ACS) or heart failure (HF), or specific trial types, leaving important gaps in understanding broader enrollment trends.^5,15,16^ Moreover, many studies have not accounted for sex-based differences in disease prevalence, a critical factor when contextualizing participation rates.^17^

The underrepresentation of women in CV trials is driven by intersecting barriers that affect diagnosis, eligibility, participation, analysis, and leadership. These include diagnostic bias and phenotype exclusion; eligibility barriers, such as comorbidities, reproductive considerations (including pregnancy and breastfeeding risk), and age-related exclusions; socioeconomic and logistical barriers to participation, such as caregiving responsibilities and scheduling constraints^15,16^; insufficient sex-specific reporting and analysis^17,18,19^; and structural factors, including the underrepresentation of women in trial leadership. Although incremental progress has been made, disparities persist, particularly in high-risk conditions like ACS and HF, where sex-based differences in drug efficacy, adverse effects, and procedural outcomes remain underexamined due to limited enrollment.^20,21^

Finally, little is known about trial characteristics or strategies that promote greater participation of women, complicating efforts to address these disparities. Recognizing the potential for heterogeneity by region, therapeutic area, intervention type, and sponsor type, this systematic review provides a comprehensive evaluation of women’s participation in CV randomized clinical trials conducted between 2017 and 2023. By analyzing enrollment trends across a broad set of variables, this study aims to identify both progress and persistent gaps, offering guidance for future improvement in trial design and equity.

## Methods

This systematic review did not require institutional review board approval because it exclusively used publicly available data, ensuring no direct involvement of human participants or collection of identifiable private information. This systematic review follows the Preferred Reporting Items for Systematic Reviews and Meta-analyses (PRISMA) reporting guideline for systematic reviews and meta-analyses (eFigure in Supplement 1).

Using ClinicalTrials.gov, a US government resource managed by the National Library of Medicine and National Institutes of Health (NIH), we searched for CV and cardiometabolic trials. The search terms used were *cardiovascular diseases*, *cardiometabolic*, and *obesity* for condition/disease, *interventional* for study type, *completed* for study status, and *with results* for study result status. The study search included trials enrolling adults aged at least 18 years. After trial selection, age stratification was applied post hoc into groups of 19 to 55, 56 to 60, 61 to 65, and older than 65 years, based on median age reported. The primary completion date was between January 1, 2017, and December 31, 2023. Trials were excluded if the disease type was not one of the 10 preidentified CV (stroke, arrhythmia, coronary heart disease [CHD], ACS, pulmonary hypertension [PH], HF, hypertension, diabetes, dyslipidemia, obesity, or multiple outcomes) or cardiometabolic diseases, there were fewer than 20 participants, information on sex proportion was not given or provided (4 trials excluded), or the median age of the study population was younger than 18 years. The 10 disease categories were selected based on the most commonly studied CV and cardiometabolic conditions in ClinicalTrials.gov and to ensure feasibility of prevalence matching; conditions such as valvular, aortic, or vascular disease were excluded due to heterogeneity in definitions and lack of consistent prevalence estimates suitable for participation:prevalence ratio (PPR) calculation. This study was registered in the International Prospective Register of Systematic Reviews (PROSPERO; identifier: CRD42024617258).^22^

### Data Extraction

Trials meeting inclusion criteria within the 10 preidentified disease categories were selected for data extraction. Data were extracted by N.R.B.B., N.T., and V.P., and the data were independently verified by F.B.R. and J.V.M. Trials were screened, and those that met the criteria were selected for the data extraction process. The following trial characteristics were extracted: National Clinical Trial number, year of primary completion date, disease type, sponsor and sponsor type, intervention and intervention type, trial location and region of enrollment, total sample size, number and proportion of women enrolled, age in years and SD (if available), and inclusion and exclusion criteria. The type of intervention was divided into drug, device, lifestyle intervention, procedure, or multiple or other interventions. Sponsor type was categorized into industry, research institute (including hospitals, medical centers, and/or universities), government (including the NIH and Department of Veterans Affairs, and other federal agencies when specified), research institute and industry, research institute and government, multiple sponsors, and individual sponsors. Detailed categorization of independent or nonacademic research sponsors was not feasible due to limitations in ClinicalTrials.gov sponsor reporting. Trial location and region of enrollment were categorized into US, Europe, North America (except US), Asia, South America, Africa, and Australia. Trials conducted in more than 1 region were considered global. Age groups included median ages within 19 to 55, 56 to 60, 61 to 65, and older than 65 years if median age was reported. Trial sizes (number of participants) were divided into 4 groups based on quartiles (Qs), with Q1 including 47 participants or fewer; Q2, 48 to 124 participants; Q3, 125 to 398 participants; and Q4, 399 participants or more. To assess the representation of women relative to disease prevalence, we collected sex-specific prevalence data from large, epidemiologic, population-based sources, including American Heart Association reports, Global Burden of Disease (GBD) data, and country or regional studies (eTable in Supplement 1). Multiple data sources were intentionally used to capture regional specificity and ensure the most contemporaneous estimates. We acknowledge that variability in data quality and heterogeneity across regions may limit direct comparability. We calculated the women’s PPR for each trial, defined as the proportion of women enrolled divided by the proportion of women with the disease in the corresponding population. A PPR of 1 indicates proportional representation, with values less than 0.8 suggesting underrepresentation and greater than 1.2 suggesting overrepresentation. Thresholds between 0.8 and 1.2 are commonly used as pragmatic indicators of adequate representation, reflecting an approximate ±20% margin to account for sampling variability and expected demographic fluctuations.

### Statistical Analysis

The proportion of women and the ratio of female:male (F:M) participation was computed for every study. Descriptive statistics were obtained summarizing these proportions in terms of comorbidities, age category, intervention type, location of trial, trial size, and sponsors. PPRs were computed for each trial using the prevalence estimates. The differences among groups in terms of proportion of female participants, F:M ratio, and PPR were assessed using Kruskal-Wallis test, followed by Dunn post hoc comparison procedure using 2-sided 5% level of significance. Nonparametric trend tests were used to assess trend of change of PPR across time. Statistical analyses were mainly conducted using STATA version 14. Data were analyzed from March to April 2025.

## Results

**Figure 1.**
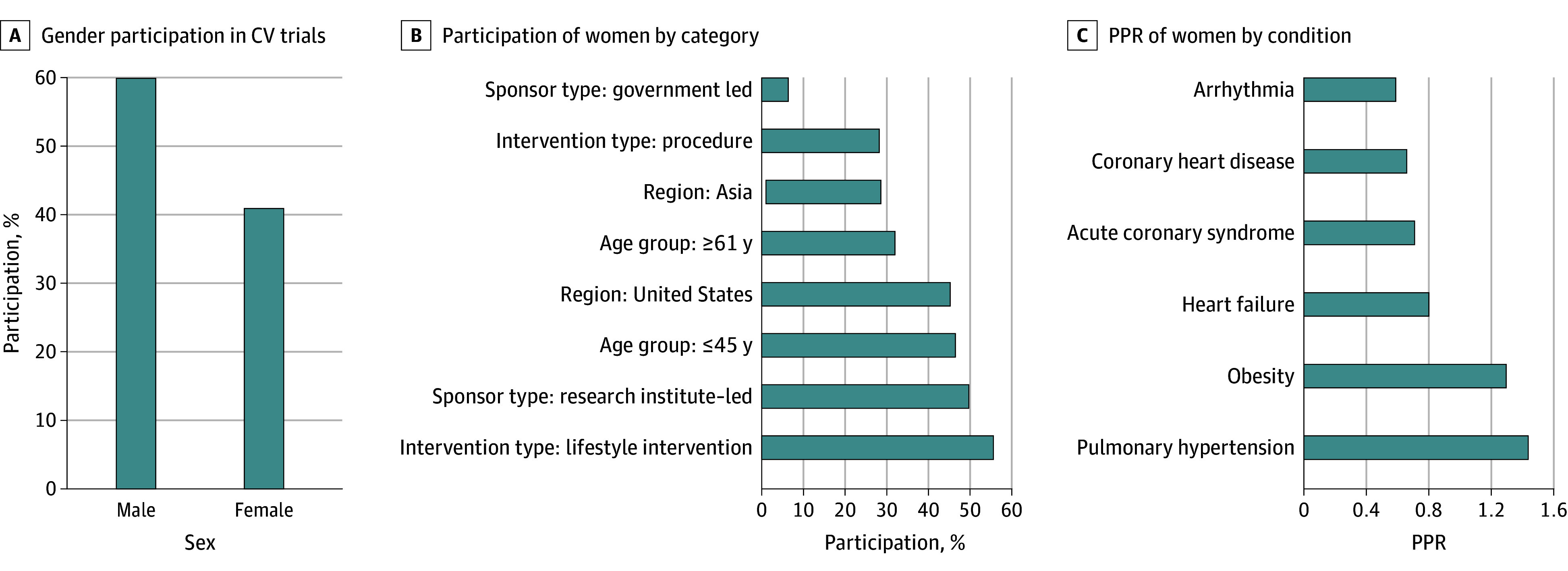
Women’s Participation in Cardiovascular (CV) Trials, 2017-2023 PPR indicates population:prevalence ratio.

A total of 2304 clinical trials were screened, of which 1079 clinical trials were included in the final analysis after exclusion (eReferences in Supplement 1). These trials encompassed 1 396 104 study participants, of whom 571 641 (41.0%) were women (Figure 1). Most of the included trials focused on obesity (243 trials [22.5%]), HF (190 trials [17.6%]), and cardiac arrhythmias (159 trials [14.7%]). Some trials investigated more than 2 comorbidities (120 trials [11.1%]). In terms of trial size, studies on obesity and hypertension had the highest participation of women, at 58.1% and 50.3%, respectively. Most trials involved patients aged between 19 and 55 years (330 trials [30.6%]). Moreover, participation of women was highest among trials involving younger participants, with trials of participants aged 19 to 55 years including 46.7% women, and declined with increasing median age (≥61 years). Approximately half of the trials were drug treatment trials (543 trials [50.3%]), where participation of women was relatively low (34.2% women). However, female participation varied by disease type. Drug trials for hypertension and dyslipidemia had comparatively higher enrollment of women, with hypertension drug trials showing a significant upward trend during the COVID-19 pandemic years. Although device trials are often associated with lower female participation, the proportion of women did not differ significantly across intervention types (drug, device, or lifestyle). Conversely, higher participation of women was observed in trials on lifestyle interventions (55.8%).

The trials were predominantly sponsored by industry (428 trials [39.7%]) and research institutions, including universities (353 trials [32.7%]). Trials sponsored by research institutions involved more women (50.0% women) compared with those sponsored by industry (37.3% women). Many of the trials were conducted in the US (676 trials [62.7%]), while 231 trials (21.41%) were conducted at multiple trial sites globally. Most trials had more than 399 participants (682 trials [63.2%]), which represented Q4 of sample sizes in our dataset but does not reflect large-scale enrollment typical of late-phase CV trials, and participation did not differ significantly across trial sizes (Table).

**Table.** Clinical Trial Characteristics and Participant Demographics

Category	No. (%)		
	Trials	Sample size	Female participants
Overall	1079 (100)	1 396 104 (100)	571 641 (41.0)
Comorbidities			
Multiple outcomes	120 (11.1)	114 899 (8.2)	44 643 (38.9)
Stroke	35 (3.2)	31 448 (2.3)	11 278 (35.9)
Arrhythmia	159 (14.7)	463 140 (33.2)	193 040 (41.7)
Coronary heart disease	107 (9.9)	157 295 (11.3)	48 443 (30.8)
Acute coronary syndrome	43 (4.0)	82 400 (5.9)	18 174 (22.1)
Pulmonary hypertension	44 (4.1)	20 770 (1.5)	5758 (27.7)
Heart failure	190 (17.6)	127 825 (9.2)	47 829 (37.4)
Hypertension	77 (7.1)	34 010 (2.4)	17 099 (50.3)
Diabetes	34 (3.2)	134 356 (9.6)	61 536 (45.8)
Dyslipidemia	27 (2.5)	46 462 (3.3)	17 213 (37.1)
Obesity	243 (22.5)	183 499 (13.1)	106 628 (58.1)
Sponsor			
Research institute	353 (32.7)	276 925 (19.8)	138 439 (50.0)
Industry	428 (39.7)	484 126 (34.7)	180 875 (37.4)
Government	49 (4.5)	40 573 (2.9)	2663 (6.6)
Research institute and industry	93 (8.6)	512 041 (36.7)	210 835 (41.2)
Research institute and government	121 (11.2)	54 445 (3.9)	25 942 (47.7)
Multiple sponsors	26 (2.4)	27 282 (2.0)	12 694 (46.5)
Individual	9 (0.8)	712 (0.1)	193 (27.1)
Median age, y			
≤55	330 (30.6)	513 368 (36.8)	239 759 (46.7)
56-60	165 (15.3)	70 518 (5.1)	28 217 (40.0)
61-65	236 (21.9)	281 414 (20.2)	90 478 (32.2)
>65	258 (23.9)	339 541 (24.3)	109 549 (32.3)
Not reported	90 (8.3)	191 263 (13.7)	103 638 (54.2)
Intervention			
Other or multiple	48 (4.5)	114 383 (8.2)	52 376 (45.8)
Drug	543 (50.3)	539 614 (38.7)	184 503 (34.2)
Device	251 (23.3)	512 216 (36.7)	217 424 (42.5)
Lifestyle intervention	193 (17.9)	190 168 (13.6)	106 078 (55.8)
Procedure	44 (4.1)	39 723 (2.9)	11 260 (28.4)
Region			
Global	231 (21.4)	453 616 (32.5)	151 478 (33.4)
US	676 (62.7)	889 759 (63.7)	403 893 (45.4)
Europe	94 (8.7)	13 300 (1.0)	4303 (32.4)
North America (except US)	18 (1.7)	3773 (0.3)	1460 (38.7)
Asia	52 (4.8)	34 873 (2.5)	10 044 (28.8)
South America	2 (0.2)	215 (0.0)	109 (50.7)
Africa	5 (0.5)	464 (0.0)	354 (76.3)
Australia	1 (0.1)	104 (0.0)	0
Trial size, participants, No.			
≤47	325 (30.1)	11 402 (0.8)	5512 (48.3)
48-124	45 (4.2)	2644 (0.2)	1361 (51.5)
125-398	27 (2.5)	1697 (0.1)	860 (50.7)
≥399	682 (63.2)	1 380 361 (98.9)	563 908 (40.9)

### Representation of Women in CV Trials

**Figure 2.**
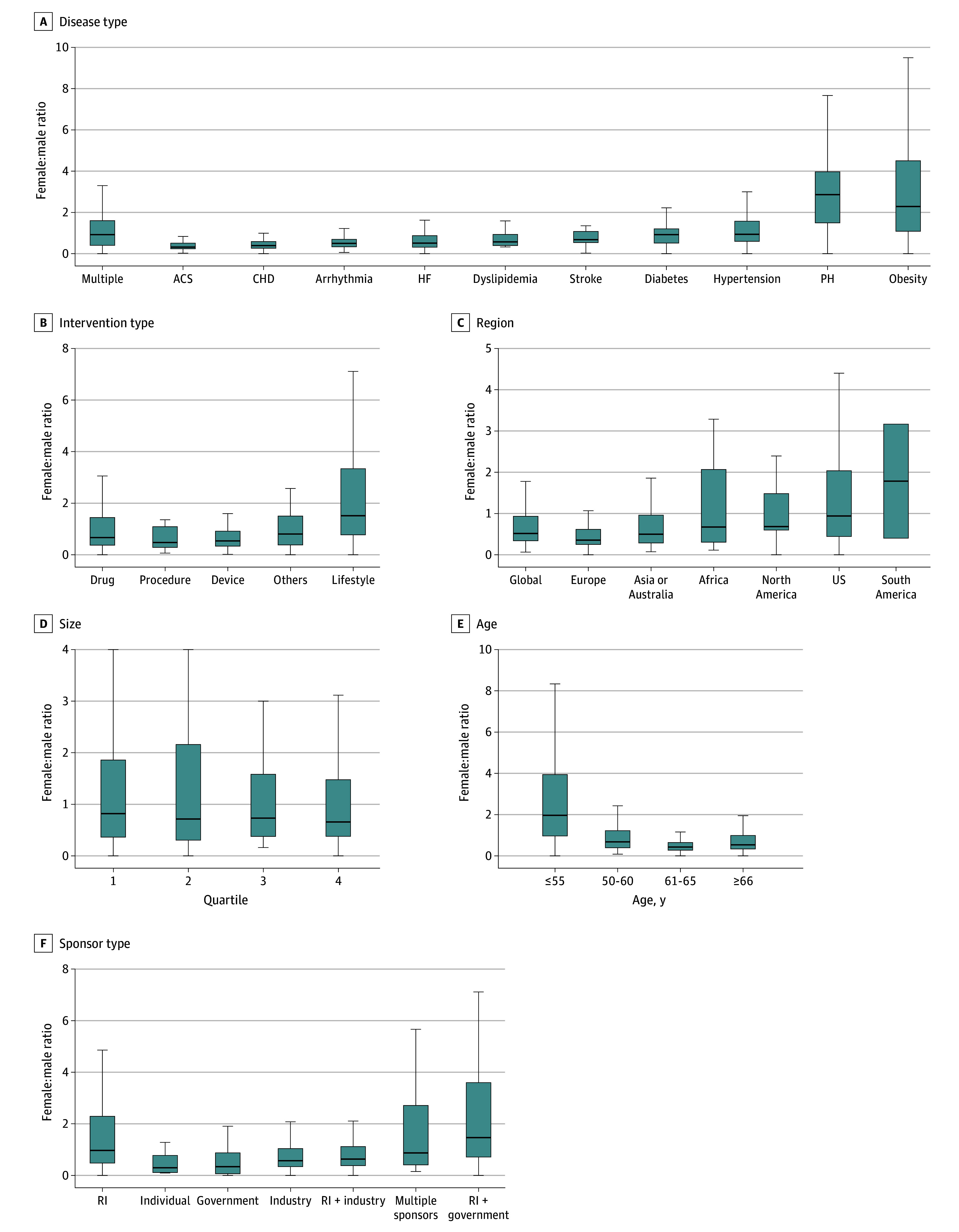
Median Female:Male Ratio in 1079 Trials ACS indicates acute coronary syndrome; CHD, coronary heart disease; HF, heart failure; PH, pulmonary hypertension; RI, research institute. Trial size quartiles are 1, 47 participants or fewer; 2, 48 to 124 participants; 3, 125 to 398 participants; and 4, 399 participants or more. Black lines indicate median; boxes, IQRs; whiskers, ranges.

The proportion of women varied substantially by disease category. Women’s participation was lowest in studies on arrhythmia (median [IQR] F:M ratio, 0.50 [0.33-0.70]), CHD (median [IQR] F:M ratio, 0.39 [0.26-0.59]), ACS (median [IQR] F:M ratio, 0.32 [0.24-0.51]), and HF (median [IQR] F:M ratio, 0.51 [0.31-0.87]). In contrast, participation was highest in studies on obesity (median [IQR] F:M ratio, 2.29 [1.08-4.50]) and PH (median [IQR] F:M ratio, 2.86 [1.50-3.97]) (Figure 2). Trials sponsored by research institutes enrolled more women (median [IQR] F:M ratio, 0.97 [0.48-2.29]) than those sponsored by industry (median [IQR] F:M ratio, 0.57 [0.34-1.04]) or government (median [IQR] F:M ratio, 0.34 [0.07-0.87]). Women’s participation was also higher in CV trials enrolling younger populations (age ≤55 years: median [IQR] F:M ratio, 1.97 [0.96-3.94]) compared with older age groups, largely reflecting disease areas with younger onset (eg, obesity, hypertension, lifestyle interventions). Studies of lifestyle interventions had greater participation (median [IQR] F:M ratio, 1.51 [0.77-3.34]). Trials conducted in the US (median [IQR] F:M ratio, 0.94 [0.44-2.04]) and other North American sites (median [IQR] F:M ratio, 0.69 [0.60-1.48]) had higher recruitment of women than those conducted elsewhere. Notably, participation did not differ significantly by intervention type (drug, device, or lifestyle).

**Figure 3.**
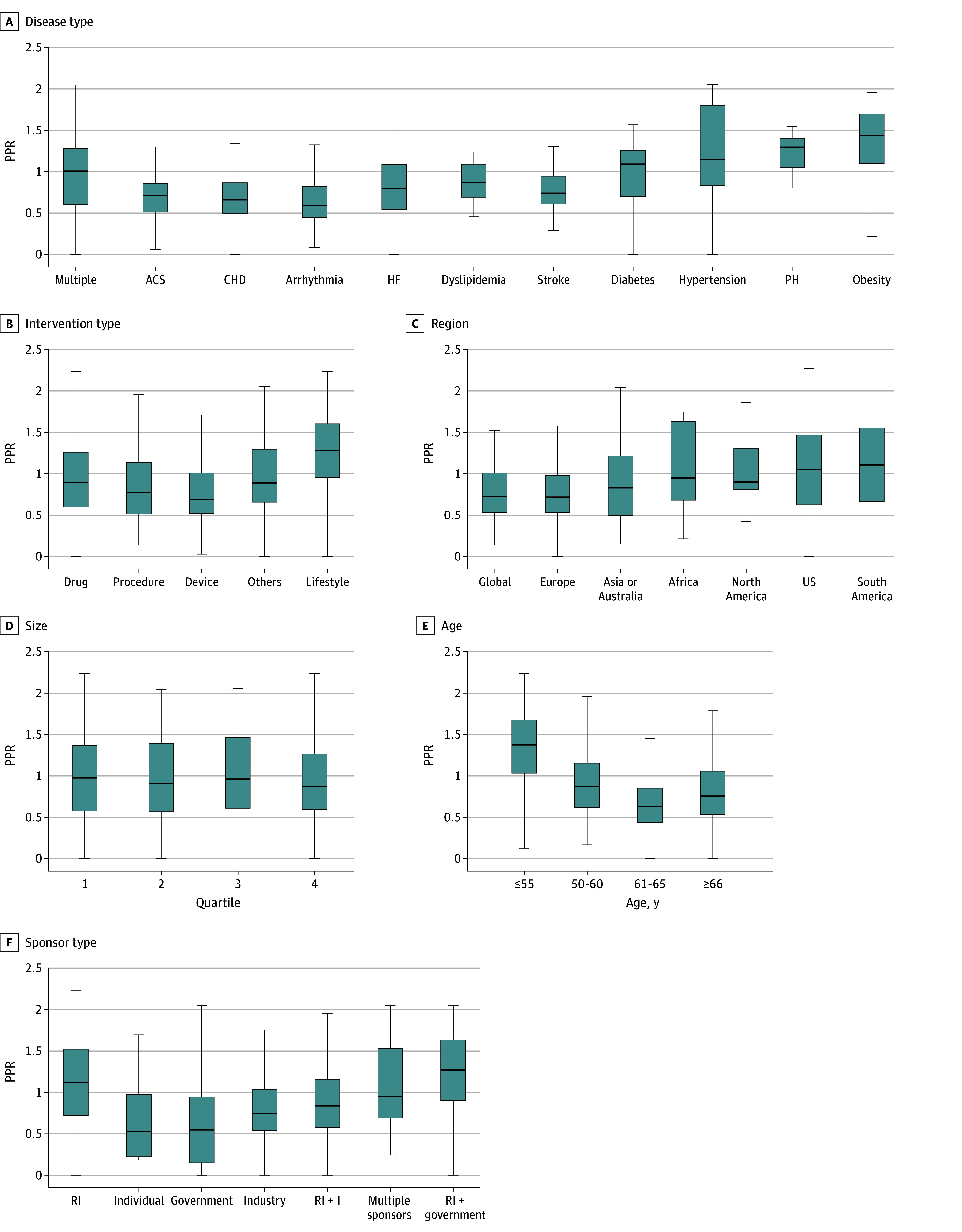
Median Participation:Prevalence Ratio (PPR) in 1079 Trials ACS, acute coronary syndrome; CHD, coronary heart disease; HF, heart failure; PH, pulmonary hypertension; RI, research institute. Trial size quartiles are 1, 47 participants or fewer; 2, 48 to 124 participants; 3, 125 to 398 participants; and 4, 399 participants or more. Black lines indicate median; boxes, IQRs; whiskers, ranges.

The PPRs for women were lowest for atrial fibrillation (median [IQR] PPR, 0.59 [0.45-0.82]), CHD (median [IQR] PPR, 0.66 [0.50-0.86]), ACS (median [IQR] PPR, 0.71 [0.51-0.86]), and stroke (median [IQR] PPR, 0.74 [0.61-0.95]), whereas higher PPRs were observed for obesity (median [IQR] PPR, 1.44 [1.10-1.70]) and PH (median [IQR] PPR, 1.30 [1.05-1.40]) (*P* < .001). These findings reflect underlying sex-specific prevalence patterns, with obesity and PH consistently showing higher female prevalence in population-based data (Table). CV trials sponsored by research institutes consistently had higher PPR of women (median [IQR] PPR, 1.12 [0.72-1.52]) compared with industry-funded trials (median [IQR] PPR, 0.74 [0.54-1.04]) or government-funded trials (median [IQR] PPR, 0.55 [0.15-0.95]) (*P* < .001). Trials that included younger participants (ie, age 18-55 years) demonstrated a significantly higher PPR of women (median [IQR] PPR, 1.37 [1.03-1.68]) compared with those focusing on older age groups. Participation of women was adequate for CV trials on drugs (median [IQR] PPR, 0.90 [0.60-1.26]) and was highest for lifestyle interventions (median [IQR] PPR, 1.28 [0.95-1.60]) (*P* < .001). CV trials with multiple sites globally (median [IQR] PPR, 0.72 [0.54-1.01]) and those done in Europe (median [IQR] PPR, 0.71 [0.53-0.98]) had lower PPRs for women compared with trials done in the US (median [IQR] PPR, 1.05 [0.63-1.47]), North America (median [IQR] PPR, 0.90 [0.81-1.30]), South America (median [IQR] PPR, 1.11 [0.66-1.55]), and Africa (median [IQR] PPR, 0.95 [0.68-1.63]) (Figure 3).

**Figure 4.**
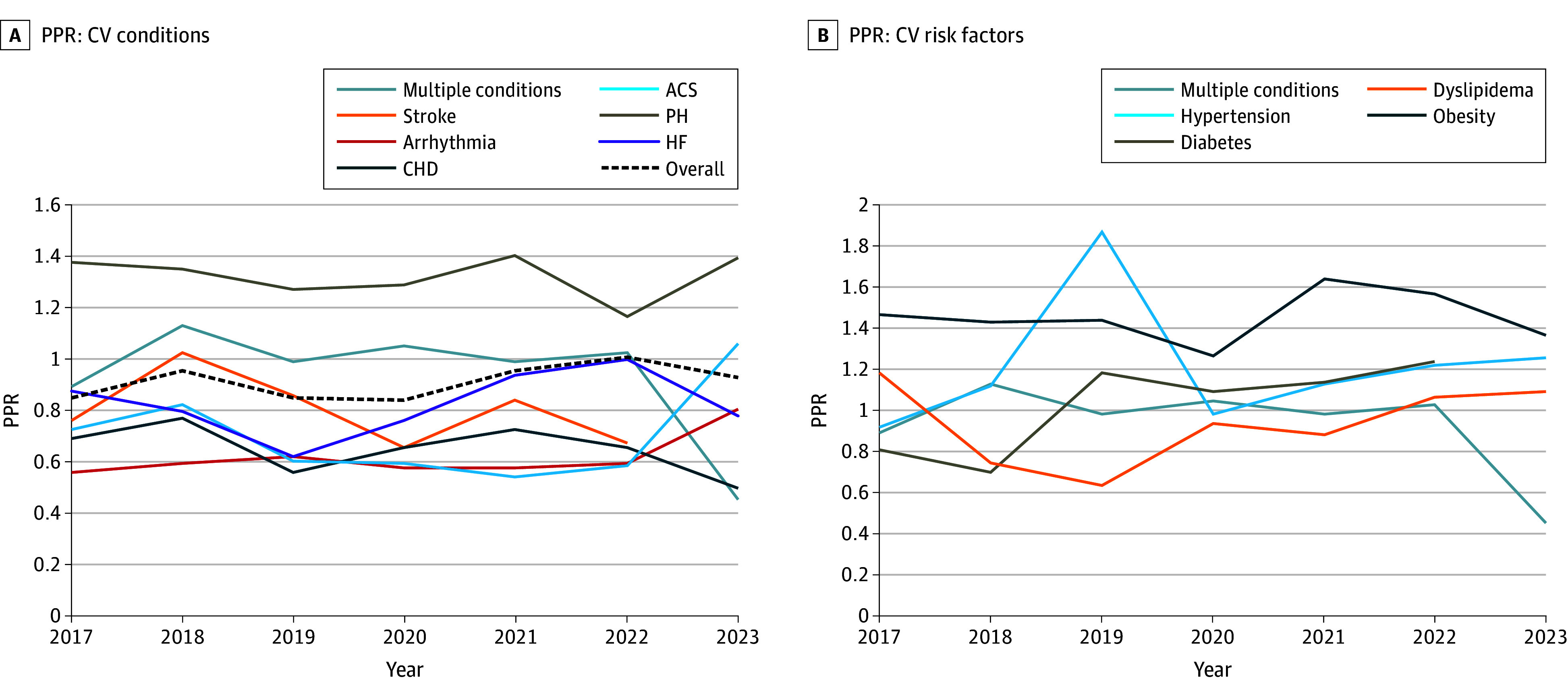
Median Participation:Prevalence Ratio (PPR) in Cardiovascular (CV) Trials From 2017 to 2023 ACS, acute coronary syndrome; CHD, coronary heart disease; HF, heart failure; PH, pulmonary hypertension.

Using Cuzick nonparametric trend analysis, there was no significant change in women’s participation from 2017 to 2024 (*z* = 1.91; *P* = .06). Clinical trials on HF demonstrated significantly increased women participation from 2017 to 2023 (*z* = 1.99; *P* = .046). Trials done for obesity and PH had the highest representation of women, and this trend was consistent (obesity: *z* = 0.38; *P* = .71; PH: *z* = −0.27; *P* = .79). CV trials for arrhythmias (*z* = 0.62; *P* = .53) and CHD (*z* = −1.15; *P* = .25) consistently had the lowest representation of women. During the COVID-19 pandemic years (2019-2022), a significant upward trend in participation of women (PPR, 0.98; *z* = 3.01; *P* = .003) was observed both for trials that were beginning and finishing enrollment during this period. This upward trend was mainly attributed to a significant increase in participation of women for studies on hypertension (PPR, 0.82; *z* = 3.40; *P* = .001), and to a lesser degree, an increase in studies done on dyslipidemia (PPR, 0.88; z = 1.86; *P* = .06) and obesity (PPR, 1.36; z = 0.091; *P* = .09), although the differences were not statistically significant (Figure 4).

### Disease Prevalence and Enrollment Patterns

We examined women’s disease prevalence in the US (eTable in Supplement 1) alongside PPRs to assess enrollment patterns across conditions. Atrial fibrillation or arrhythmia (population prevalence, 65%; PPR, 0.59) and stroke (population prevalence, 57%; PPR, 0.74) showed the greatest discordance, with high prevalence but low PPRs. CHD (population prevalence, 43%; PPR, 0.66) and ACS (population prevalence, 34%; PPR, 0.79) demonstrated consistent underrepresentation with moderate to high prevalence. HF (population prevalence, 45%; PPR, 0.80), diabetes (population prevalence, 44%; PPR, 0.90), dyslipidemia (population prevalence, 55%; PPR, 0.88), and hypertension (population prevalence, 49%; PPR, 0.82) had borderline discordance. PH (population prevalence, 57%; PPR, 1.30) and obesity (population prevalence, 51%; PPR, 1.44) demonstrated concordance, with high prevalence and proportional or overrepresentation.

## Discussion

The findings of this systematic review highlight that, although significant differences exist in the burden, presentation, and outcomes of CV disease between men and women, women remained consistently underrepresented in clinical trials that inform evidence-based care. Our analysis highlights both progress and persistent challenges in representation of women within CV trials. Enrollment patterns continue to vary by disease focus, intervention type, age demographics, and sponsor characteristics. Several underlying factors continue to influence these enrollment patterns, including diagnostic biases, reproductive barriers, age-related exclusions, socioeconomic constraints, gaps in sex-specific reporting, and underrepresentation in trial leadership.

### Sex-Based Diagnostic Bias and Phenotype Exclusion

Historically, women’s CV health has been assessed and managed using male-centric models that overlook sex-specific biological and sociocultural factors. This bias has shaped diagnostic criteria and treatment paradigms for decades, often excluding phenotypes more prevalent in women, such as nonobstructive coronary artery disease.^23,24^ Younger women presenting with ST-elevation myocardial infarction also face delayed diagnosis and undertreatment due to misconceptions, poor communication,^25^ and the lack of awareness, especially in Hispanic and Black women,^26^ leading to poor outcomes.^27,28^

Although multiple policies and guidelines over the past decade have aimed to improve representation and sex-specific analysis—including the establishment of the US Food and Drug Administration (FDA) Office of Women’s Health, the NIH Inclusion Policy, the NIH Sex as a Biological Variable policy, and recent American College of Cardiology/American Heart Association recommendations^11,29^—our analysis shows that enrollment and design adjustments in clinical trials remain.

Our findings demonstrate that women’s participation was highest in obesity and PH trials (PPRs >1.2), where overrepresentation likely corresponds to both increased disease prevalence and improved enrollment strategies.^30,31^ Conversely, enrollment remained lower in traditionally male-perceived conditions, such as arrhythmia, CHD, and ACS, where PPRs remained well below 1 despite ongoing efforts.^32,33^ Participation in HF trials has improved, with PPRs increasing from 0.5 (2010-2017)^31^ to 0.8 in our analysis. Historically, women represented only 20% to 30% of HF trial participants, but recent data show enrollment reaching 58% in HF with preserved ejection fraction trials compared with 24% in HF with reduced ejection fraction trials, reflecting growing recognition of female-predominant phenotypes.^32^

However, examining PPRs alone does not fully capture the impact of disparities. Among diseases with the lowest PPRs, atrial fibrillation and stroke demonstrated much higher prevalence among women (65% and 57%, respectively) compared with CHD and ACS (43% and 34%, respectively). This mismatch poses a major public health concern, as highly prevalent conditions with low trial representation amplify sex-specific evidence gaps. Contributing factors likely include the underenrollment of older adults, procedural exclusions, and physician bias in device-based trials (eg, ablation) for atrial fibrillation, as well as stringent exclusion criteria and male-skewed enrollment in stroke studies.

This pattern underscores the need for prioritizing enrollment improvements not only based on PPR but also on disease burden. Addressing these gaps requires both improved trial inclusion and broader strategies, such as sex- and gender-based education, clinician awareness, and tools like coronary artery calcium scoring for individualized risk.^29,34^

### Reproductive Barriers

Even when diagnostic eligibility is met, additional eligibility barriers arise for reproductive-age women due to persistent safety concerns and exclusionary criteria. Concerns about drug and device safety in pregnant and lactating women continue to drive exclusionary eligibility criteria. The FDA has eased certain prohibitions, such as downgrading pregnancy risk warnings for statins, and NIH policies now emphasize reproductive inclusion. However, challenges persist, particularly in industry-sponsored trials where liability concerns remain a deterrent.^35,36,37^ Trials led by academic research institutions enrolled more women than those sponsored by industry or government, highlighting the role of sponsor policies in shaping inclusion.^32^ To overcome these barriers, trials should accommodate reproductive-age women, ensure patient-centered consent, and collect reproductive history to inform risk.^38,39,40,41,42^ Clinicians must also clearly communicate trial risks and benefits, especially for device and procedural studies, where reproductive concerns drive exclusion.^17,39^

### Age-Related Exclusions

Beyond reproductive status, age-based eligibility exclusions further limit the inclusion of women, especially older adults, who represent a growing at-risk population for CV events. Many CV conditions, including HF and coronary artery disease, manifest later in life for women compared with men. Nevertheless, upper age limits in eligibility criteria remain common, excluding older women who represent a substantial at-risk population. While the NIH’s 2019 Inclusion Across the Lifespan policy encourages broader age representation, our findings—and those of other studies—indicate limited progress since its introduction.^25^ To address this barrier, eligibility criteria should be revised to reflect population age demographics, and enrollment data should be systematically monitored to ensure adequate inclusion of older women.

### Socioeconomic and Logistical Barriers

Even when eligibility criteria are met, practical and socioeconomic burdens frequently prevent participation. Women’s participation in clinical trials is often impeded by caregiving responsibilities, transportation challenges, and rigid study schedules. These burdens are amplified for women from underrepresented racial and ethnic groups (eg, American Indian, Black, Hispanic/Latina, and Pacific Islander women).^5,20,32,43,44,45^ Multiple reviews of randomized clinical trials, along with our own analysis, show that women are more represented in lifestyle and drug trials, which typically involve fewer logistical demands and may align with gendered health perceptions and patterns of health-seeking behavior, especially around obesity (the highest PPR, at 1.44).^46,47,48^ In contrast, HF and coronary artery disease trials—often requiring devices or invasive procedures—continued to demonstrate low PPRs, reflecting both logistical barriers and potentially higher perceived personal risk. Furthermore, women tend to enroll more readily in primary prevention trials, such as those focused on hypertension and lifestyle interventions, where PPRs approached or achieved proportional representation and the perceived personal risk is lower. Participation remains lower in higher-risk procedural trials.^49^ Over the 6-year study period (2017-2023), aggregate PPRs improved modestly (from approximately 0.89 to approximately 0.98), and studies enrolling between 2019 and 2022 (COVID-19 pandemic years) had a relative increase in women’s participation, particularly in hypertension trials. Geographical variations also emerged: US-based trials achieved higher enrollment of women and PPRs than European or Asian studies, reflecting systemic differences in health care infrastructure, recruitment strategies, and sociocultural norms.

The COVID-19 pandemic tested the resilience of trial structures but also spurred innovation. To our knowledge, this is the first study to document increased women’s participation in CV trials during the pandemic. Remote assessments, telehealth, and decentralized enrollment models helped sustain—and, in some cases, improve—women’s participation despite widespread disruptions.^5,42,43,50,51,52,53,54^ Adaptations, such as virtual study visits, remote data collection, and telemedicine, guided by the FDA, European Medicines Agency, and Heart Failure Academic Research Consortium reduced logistical and socioeconomic barriers and likely facilitated greater participation of women.^53,55,56,57^ To improve inclusivity, expanding decentralized or hybrid trial designs, simplifying protocols, increasing scheduling flexibility, and offering targeted outreach to caregivers and underserved populations are key priorities.^44,57,58,59^

### Insufficient Sex-Specific Reporting

Among women who do enroll, insufficient sex-stratified reporting limits the translation of participation into meaningful, sex-specific evidence. Despite longstanding advocacy, fewer than one-third of phase 3 CV trial publications report sex-stratified outcomes.^56^ This omission limits clinicians’ ability to provide evidence-based, sex-specific care and obscures potential treatment disparities. Enhanced enforcement of existing NIH and FDA mandates for sex-disaggregated data reporting is urgently needed. Expanding these requirements to all trial phases and incentivizing the publication of sex-specific outcomes will be critical to improving transparency and advancing equitable care.^60,61,62^

### Underrepresentation in Trial Leadership

Finally, structural influences—including the underrepresentation of women in trial leadership—continue to shape design, eligibility, and reporting practices, reinforcing these disparities. Multiple studies confirm that CV trials led by female investigators enroll more women participants.^63,64^ Yet, female leadership remains scarce, perpetuating research priorities that may overlook sex-specific disease presentations and patient-reported outcomes.^65^ Promoting gender diversity in trial leadership through funding incentives, mentorship initiatives, and deliberate selection of diverse leadership teams can enhance inclusion.^63,64^ Funding agencies can drive this change by incentivizing diverse leadership teams and expanding mentorship programs for women physician-scientists. Engaging patients and community advocates in trial design and recruitment can further ensure that studies address sex- and gender-specific challenges.

Addressing these persistent barriers requires a multifaceted, collaborative effort across investigators, sponsors, regulatory agencies, and patient communities. Strengthening sex- and gender-equitable practices in trial design, leadership, and reporting will be essential for translating scientific advances into improved CV outcomes for women. As trial enrollment and design continue to evolve, sustained accountability and innovation will be critical to closing the sex gap in CV research.

### Limitations

This study has several limitations. Sex-specific prevalence data were drawn from multiple large, population-based sources to ensure contemporaneous and regionally specific estimates, but heterogeneity across sources may limit comparability between disease categories and regions. We used multiple sources to maximize regional and disease-specific granularity, particularly for conditions underrepresented in the GBD study or for which GBD provided only high-level estimates. However, we acknowledge that heterogeneity across sources may limit comparability among disease categories and regions. In future work, use of a single, standardized source, such as the GBD study, may enhance consistency across disease categories. Reliance on ClinicalTrials.gov likely underrepresents trials registered exclusively in non-US or regional databases. The descriptive design precluded multivariate modeling of independent predictors of sex representation, which we acknowledge as a future direction. Certain disease categories, including valvular, aortic, and vascular conditions, were excluded due to inconsistent definitions and the lack of standardized prevalence estimates, which would have compromised the consistency of our PPR calculations. Nonetheless, underrepresentation in these areas remains an important concern and a focus for future research. Most heart failure trials did not stratify by HF with preserved ejection fraction vs with reduced ejection fraction, limiting analysis by subtype. We also could not assess whether women were offered enrollment at the same rate as men, as most trials do not report sex-specific screening or approach data. Sponsor categorization was constrained by ClinicalTrials.gov’s reporting structure, and race, ethnicity, and age-specific disparities were not systematically assessed due to data limitations. Furthermore, while we observed modest improvements in women’s enrollment during the COVID-19 pandemic, future studies across other disease areas (eg, oncology or infectious disease) are needed to determine whether similar gains occurred in response to decentralized and remote trial innovations.

## Conclusions

This systematic review highlights both progress and persistent gaps in the representation of women in CV trials. Increased participation was observed in studies of obesity, PH, HF, and primary prevention studies, reflecting increasing awareness and evolving trial designs. However, underrepresentation persisted in several high-risk and procedural fields, limiting the generalizability of trial outcomes and perpetuating disparities in evidence-based care. Continued efforts to implement inclusive trial designs, improve outreach, and enforce diversity policies are essential to achieving equitable representation in CV research.

## Notes

Supplement 1. **eFigure.** PRISMA Flowchart **eTable.** Estimation of the Percentage of Women in Eleven Cardiovascular Disease Categories by Region
eReferences.

Supplement 2.
Data Sharing Statement
