# Error in Table

**DOI:** 10.1001/jamanetworkopen.2025.40119

**Published:** 2025-10-01

In the Original Investigation titled “Participation of Women in Cardiovascular Trials From 2017 to 2023: A Systematic Review,”^1^ published August 31, 2025, the Table incorrectly gave the percentage of female participants with pulmonary hypertension as 22.1%. This should have been described as 27.7%. There was also an error in the presentation of coauthor Dr Lala’s name. This article has been corrected.^1^
