# Supplementary material for: Participation of Women in Cardiovascular Trials From 2017 to 2023: A Systematic Review
Source: JAMA Netw Open. 2025 Aug 31;8(8):e2529104. doi: 10.1001/jamanetworkopen.2025.29104 (PMC12400126; doi:10.1001/jamanetworkopen.2025.29104)
Supplement: Supplement 1. — eFigure. PRISMA Flowchart eTable. Estimation of the Percentage of Women in Eleven Cardiovascular Disease Categories by Region eReferences. [file jamanetwopen-e2529104-s001.pdf]

Supplemental Online Content

Rivera FB, Magalong JV, Bantayan NRB, et al. Participation of women in cardiovascular trials from 2017 to 2023: a systematic review. *JAMA Netw Open*. 2025;8(8):e2529104. doi:10.1001/jamanetworkopen.2025.29104

eFigure 1. PRISMA Flowchart
eTable 1. Estimation of the Percentage of Women in Eleven Cardiovascular Disease Categories by Region
eReferences.

This supplemental material has been provided by the authors to give readers additional information about their work.

© 2025 Rivera FB et al. *JAMA Network Open*.

eFigure 1. PRISMA Flowchart

Flow of study selection for inclusion in the systematic review. ClinicalTrials.gov records with primary completion dates between January 1, 2017, and December 31, 2023, were screened. Studies were excluded due to irrelevant disease type (n = 974), small sample size (n < 20; n = 189), missing sex data (n = 4), or pediatric median age (< 18 years; n = 23).

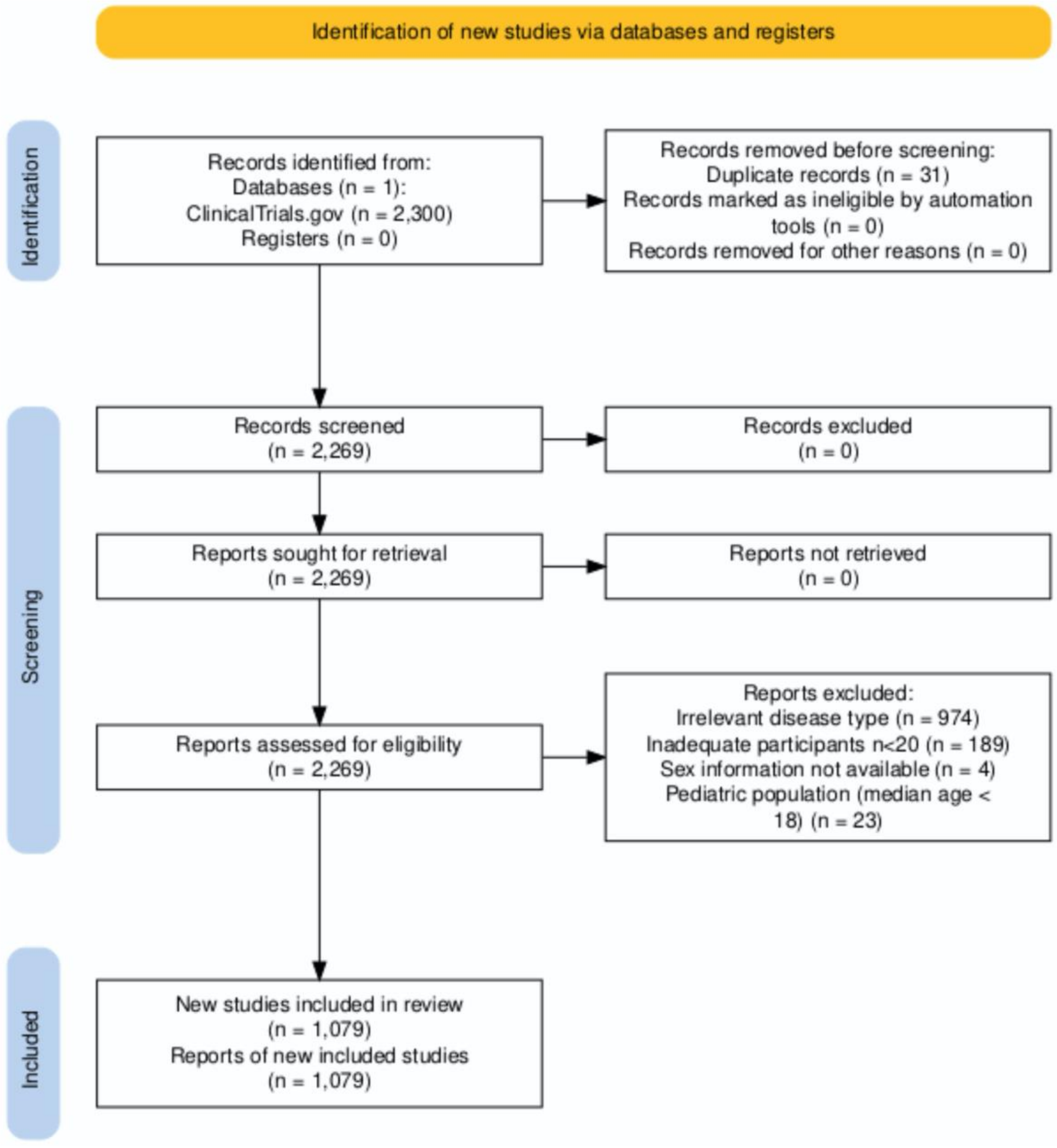

© 2025 Rivera FB et al. *JAMA Network Open*.

eTable 1: Estimation of Percentage of Females in Eleven Cardiovascular Disease Categories

This table shows the estimates of the proportion of women affected by cardiovascular and cardiometabolic conditions across various global regions, including the United States, Europe, Asia, and globally. Data sources include national registries and large epidemiological studies, with values reported as absolute numbers for total cases, number of female and male cases, and calculated proportions of women for each condition.

Disease Type	Region	Total (N)	Females(F)	Males (M)	Proportion of Women	References for Literature	PMID
**CARDIOVASCULAR DISEASE**	Global	422738385	205821777	216916608	0.4869	Global, Regional, and National Burden of Cardiovascular Diseases for 10 Causes, 1990 to 2015	28776245
	USA	127,900,000	62,500,000	65,400,000	0.4887	2024 Heart Disease and Stroke Statistics: A Report of US and Global Data From the American Heart Association.	38264914
**STROKE**	Global	7200000	4100000	3100000	0.5694	Global, Regional, and National Burden of Cardiovascular Diseases for 10 Causes, 1990 to 2015	28776245
	USA	9,400,000	5,400,000	4,000,000	0.5745	2024 Heart Disease and Stroke Statistics: A Report of US and Global Data From the American Heart Association.	38264914
	China (Asia)	45,641	18084	27,557	0.3962	Trends in In-Hospital Mortality among Patients with Stroke in China.	24651454
	Europe	6,434	2,176	4,258	0.3382	Incidence of stroke in Europe at the beginning of the 21st century.	19325154
**ARRHYTHMIA/ ATRIAL FIBRILLATION/DISORDERS OF HEART RHYTHM**	Global	52,500,000	24,650,000	27,850,000	0.4695	2024 Heart Disease and Stroke Statistics: A Report of US and Global Data From the American Heart Association.	38264914
	USA	656.1	428.9	227.2	0.6537	2024 Heart Disease and Stroke Statistics: A Report of US and Global Data From the American Heart Association.	38264914
**CORONARY HEART DISEASE**	Global	252,200,000	108,930,000	143270000	0.4319	2024 Heart Disease and Stroke Statistics: A Report of US and Global Data From the American Heart Association.	38264914
	USA	20,500,000	8,800,000	11700000	0.4293	2024 Heart Disease and Stroke Statistics: A Report of US and Global Data From the American Heart Association.	38264914

© 2025 Rivera FB et al. *JAMA Network Open*.

Disease Type	Region	Total (N)	Females(F)	Males (M)	Proportion of Women	References for Literature	PMID
	Asia	1868	857	1011	0.4588	Examining the Signs and Symptoms Experienced by Individuals With Suspected Acute Coronary Syndrome in the Asia-Pacific Region: A Prospective Observational Study.	22738683
	Europe	12231	3725	8506	0.3046	International differences in acute coronary syndrome patients' baseline characteristics, clinical management and outcomes in Western Europe: the EURHOBOP study.	24790068
**ACUTE CORONARY SYNDROME**	Global	252,200,000	108,930,000	143270000	0.4319	2024 Heart Disease and Stroke Statistics: A Report of US and Global Data From the American Heart Association.	38264914
	USA	9,300,000	3,200,000	6,100,000	0.3441	2024 Heart Disease and Stroke Statistics: A Report of US and Global Data From the American Heart Association.	38264914
	Asia	1868	857	1011	0.4588	Examining the Signs and Symptoms Experienced by Individuals With Suspected Acute Coronary Syndrome in the Asia-Pacific Region: A Prospective Observational Study.	22738683
**ACUTE CORONARY SYNDROME**	Europe	12231	3725	8506	0.3046	International differences in acute coronary syndrome patients' baseline characteristics, clinical management and outcomes in Western Europe: the EURHOBOP study	24790068
**PULMONARY HYPERTENSION**	America				0.5720	Pulmonary Hypertension Surveillance United States, 2001 to 2010	24700091
**HEART FAILURE**	Global	56,500,000	27,300,000	29,200,000	0.4832	The Global Burden of Disease Study Collaborators, The global burden of heart failure: a systematic analysis for the Global Burden of Disease Study 2021.	/

© 2025 Rivera FB et al. *JAMA Network Open*.

Disease Type	Region	Total (N)	Females(F)	Males (M)	Proportion of Women	References for Literature	PMID
**HEART FAILURE**	USA	6,700,000	3,000,000	3,700,000	0.4478	2024 Heart Disease and Stroke Statistics: A Report of US and Global Data From the American Heart Association.	38264914
	Europe	5118	1664	3454	0.3251	EURObservational Research Programme: the Heart Failure Pilot Survey (ESC-HF Pilot)	20805094
	Asia (Total)	27782	10675	17107	0.3842		
	Japan	10219	3086	7133		Trend of westernization of etiology and clinical characteristics of heart failure patients in Japan: First report from the CHART-2 Study	21436596
	Japan	5111	2303	2808		Association between length of stay, frequency of in-hospital death, and causes of death in Japanese patients with acute heart failure syndromes	23434007
	China	12452	5286	7166		Characteristics of in-hospital patients with chronic heart failure in Hubei province from 2000 to 2019	21924083
**DIABETES**	Global	525,600,000	254,800,000	270,800,000	0.4848	2024 Heart Disease and Stroke Statistics: A Report of US and Global Data From the American Heart Association.	38264914
	USA	29,300,000	12,900,000	16,400,000	0.4403	2024 Heart Disease and Stroke Statistics: A Report of US and Global Data From the American Heart Association.	38264914
**OBESITY**	USA	3,467	1,774	1,693	0.5116	2024 Heart Disease and Stroke Statistics: A Report of US and Global Data From the American Heart Association.	38264914
	Global	813,000,000	466,000,000	347,000,000	0.5732	2024 Heart Disease and Stroke Statistics: A Report of US and Global Data From the American Heart Association.	38264914
**DYSLIPIDEMIA**	USA	86,400,000	47,500,000	38,900,000	0.5498	2024 Heart Disease and Stroke Statistics: A Report of US and Global Data From the American Heart Association.	38264914
	Global				0.39	Global epidemiology of dyslipidaemias.	33833450

© 2025 Rivera FB et al. *JAMA Network Open*.

Disease Type	Region	Total (N)	Females(F)	Males (M)	Proportion of Women	Referen
**DYSLIPIDEMIA**	Europe				0.54	Global ep dyslipida
	North America				0.48	
	Africa				0.226	
	Asia (Southeast Asia)				0.29	
**HYPERTENSION**	*USA	122,400,000	59,600,000	62,800,000	0.4869	2024 He Statistics Global D Heart As
	*Global	1278000000	626000000	652000000	0.4898	Worldwic prevalen treatmen 2019: a p populatio with 104

© 2025 Rivera FB et al. *JAMA Network Open*.

eReferences Table 1. List of included trials by study title and NCT number.

	Study Title	NCT Number
1	Souriji et. al / Effects of Linagliptin on Endothelial Function	NCT02350478
2	Voskerician et. al / Piloting Healthcare Coordination in Hypertension	NCT02988193
3	Gelijns et. al / Neuroprotection In Patients Undergoing Aortic Valve Replacement	NCT02389894
4	Berman et. al / Artifact-Free High-Resolution Myocardial Perfusion MRI in Subjects With Abnormal Nuclear Myocardial Perfusion Studies	NCT01949844
5	Amann et al. / Premature Fatigue in Veterans With Heart Failure: Neuronal Influences	NCT02209610
6	Hirshberg et al. / Remote Surveillance of Postpartum Hypertension	NCT03185455
7	Depre et al. / Treadmill Cardiovascular Safety Study of Erenumab (AMG 334)	NCT02575833
8	Novartis Pharmaceuticals / Efficacy, Safety and Tolerability of Serelaxin When Added to Standard Therapy in AHF	NCT01870778
9	Spitzer et al. / Study Comparing the MiStent SES Versus the XIENCE EES Stent	NCT02385279
10	Vorderstrasse et al. / Genetic Risk and Health Coaching for Type 2 Diabetes and Coronary Heart Disease	NCT01884545
11	Novobílský et al. / Verification of the Safety of Early Discharge in Patients After Acute ST-segment Myocardial Infarction	NCT02023983
12	Patel et al. / Evaluating Increasing Physical Activity After Acute Coronary Syndrome	NCT02531022
13	Pappone et al. / AF Substrate Mapping and Guided Ablation	NCT02571218
14	Ruffner et al. / TactiCath® Contact Force Ablation Catheter Study for Atrial Fibrillation Post Approval Study	NCT02310100
15	Pfizer / Study Of The Blood Thinner, Apixaban, For Patients Who Have An Abnormal Heart Rhythm (Atrial Fibrillation) And Expected To Have Treatment To Put Them Back Into A Normal Heart Rhythm (Cardioversion)	NCT02100228
16	Liu et al. / Brilinta Taiwan Post Approval Safety Study	NCT02406248
17	Brooks et al. / Specialized Community Disease Management to Reduce Substance Use and Hospital Readmissions	NCT02059005
18	Janssen Research & Development / CANVAS - CANagliflozin cardioVascular Assessment Study	NCT01032629
19	GSK Clinical Trials / A Method Validation Study for Evaluation of Novel Treatments Limiting Pulmonary Oedema in Cardiac Failure	NCT02135861
20	Janssen Research & Development / A Study of the Effects of Canagliflozin (JNJ- 28431754) on Renal Endpoints in Adult Participants With Type 2 Diabetes Mellitus	NCT01989754
21	Jax et al. / Visual Feedback Therapy for Treating Individuals With Hemiparesis Following Stroke	NCT01662960
22	Idorsia Pharmaceuticals / Dose-finding Study With ACT-132577 (Aprocitentan) in Participants With Essential Hypertension	NCT02603809
23	Zareba et al. Ranolazine Implantable Cardioverter-Defibrillator Trial	NCT01215253
24	Hare et al. / The TRansendocardial Stem Cell Injection Delivery Effects on Neomyogenesis STudy (The TRIDENT Study)	NCT02013674
25	Betts et al. / RF Power, LSI and Oesophageal Temperature Alerts During AF Ablation (PiLOT-AF Study)	NCT02619396
26	Sanofi / Efficacy and Safety of Alirocumab Versus Usual Care on Top of Maximally Tolerated Statin Therapy in Patients With Type 2 Diabetes and Mixed Dyslipidemia (ODYSSEY DM-Dyslipidemia)	NCT02642159

© 2025 Rivera FB et al. *JAMA Network Open*.

27	Novartis Pharmaceuticals / Cardiovascular Risk Reduction Study (Reduction in Recurrent Major CV Disease Events)	NCT01327846
28	Garan et al. / Mineralocorticoid Receptor Antagonists (MRA) in Heart Failure (HF) and Loop Diuretic Resistance	NCT02585843
29	Woodbury et al. / Patient Targeted Upper Extremity Rehabilitation After Stroke	NCT01819506
30	Damush et al. / Stroke Self-Management: Effect on Function and Stroke Specific Quality of Life	NCT01507688
31	Kandzari et al. / Safety and Effectiveness of the Orsiro Sirolimus Eluting Coronary Stent System in Subjects With Coronary Artery Lesions	NCT02389946
32	Gooneratne et al. / Effects of Use of a Connected Pillbox On Medication Adherence	NCT02593032
33	Mullens et al. / Acetazolamide and Spironolactone to Increase Natriuresis in Congestive Heart Failure	NCT01973335
34	Reynolds et al. / Apixaban Evaluation of Interrupted Or Uninterrupted Anticoagulation for Ablation of Atrial Fibrillation	NCT02608099
35	Keddad et al. / Efficacy and Safety Study of F373280	NCT01831856
36	Sanofi et al. / Efficacy and Safety of Alirocumab Versus Placebo on Top of Maximally Tolerated Lipid Lowering Therapy in Patients With Hypercholesterolemia Who Have Type 1 or Type 2 Diabetes and Are Treated With Insulin (ODYSSEY DM - Insulin)	NCT02585778
37	Yang et al. / BIOTRONIK Orsiro Pre-Marketing Registration	NCT02870985
38	Amgen et al. / Safety, PK, and Efficacy of Omecamtiv Mecarbil in Japanese Subjects With Heart Failure With Reduced Ejection Fraction	NCT02695420
39	GlaxoSmithKline / Bioequivalence Study Between GSK3542503 Hydrochlorothiazide + Amiloride Hydrochloride 50 mg: 5 mg Tablets and Reference Product in Healthy Adult Participants Under Fasting Conditions	NCT03031496
40	Abbate et al. / Interleukin-1 Blockade in HF With Preserved EF	NCT02173548
41	Lyden et al. / Safety Evaluation of 3K3A-APC in Ischemic Stroke	NCT02222714
42	Patel et al. / Pragmatic, Randomized Evaluation of Statin Active Choice to Reach Improved Outcomes Based on Evidence	NCT03021759
43	Gustavson et al. / Exenatide Study of Cardiovascular Event Lowering Trial (EXSCEL): A Trial To Evaluate Cardiovascular Outcomes After Treatment With Exenatide Once Weekly In Patients With Type 2 Diabetes Mellitus	NCT01144338
44	Kasner et al. / GORE® Septal Occluder Device for Patent Foramen Ovale (PFO) Closure in Stroke Patients	NCT00738894
45	Weiss et al. / A Prospective Study of B244 Delivered as a Topical Spray to Determine Safety and Efficacy in Subjects With Elevated Blood Pressure	NCT02998840
46	Pekmezaris et al. / Telehealth Management in HF Disparity Patients	NCT02196922
47	Klein et al. / Framingham State Food Study	NCT02068885
48	Tuteja et al. / Assessment of Prospective CYP2C19 Genotype Guided Dosing of Anti-Platelet Therapy in Percutaneous Coronary Intervention	NCT02508116
49	Schiavon et al. / Gastric Bypass to Treat obEse Patients With steAdy hYpertension	NCT01784848
50	LUK et al. / Adjunct Low Frequency Repetitive Transcranial Magnetic Stimulation With Physiotherapy Enhance Upper Extremity Function Restoration	NCT02490371
51	Stardal et al. / A Study to Assess the Effect of Intravenous Dose of (aMBMC) to Subjects With Non-ischemic Heart Failure	NCT02467387
52	Takeda et al. / Cardiovascular Safety of Febuxostat and Allopurinol in Participants With Gout and Cardiovascular Comorbidities (CARES)	NCT01101035
53	Jovin et al. / Clinical Mismatch in the Triage of Wake Up and Late Presenting Strokes Undergoing Neurointervention With Trevo	NCT02142283

© 2025 Rivera FB et al. *JAMA Network Open*.

54	Boehringer Ingelheim / 24 Week Efficacy and Safety Study of Empagliflozin (BI 10773) in Hypertensive Black/African American Patients With Type 2 Diabetes Mellitus and Hypertension	NCT02182830
55	Neuzil et al. / Dipole Density Mapping in Supraventricular Tachycardia	NCT02469623
56	McEvoy et al. / The Platelet Aggregation After tiCagrelor Inhibition and FentanYl Trial (PACIFY)	NCT02683707
57	Rich-Edwards et al. / Heart Health 4 Moms Trial to Reduce CVD Risk After Preeclampsia	NCT02147626
58	Ikonomidis et al. / Effects of Agonists of Glucagon Like Peptide - 1 Receptors (GLP- 1R) on Arterial Stiffness, Endothelial Glycocalyx and Coronary Flow Reserve in Patients With Coronary Artery Disease and Patients With Diabetes Mellitus	NCT03010683
59	Russell et al. / Sub-Q Versus IV Furosemide in Acute Heart Failure	NCT02579057
60	Kavita et al. / Diuretics and Dopamine in Heart Failure With Preserved Ejection Fraction	NCT01901809
61	Gooneratne et al. Reducing Heart Failure Re-admissions by Enhancing Sleep Apnea Treatment Adherence	NCT02312765
62	Solum et al. / Safety and Efficacy of APD811 in Pulmonary Arterial Hypertension	NCT02279160
63	Frei et al. / A Randomized, Concurrent Controlled Trial to Assess the Safety and Effectiveness of the Separator 3D as a Component of the Penumbra System in the Revascularization of Large Vessel Occlusion in Acute Ischemic Stroke	NCT01584609
64	Duffy et al. / A Study of CSL112 in Adults With Moderate Renal Impairment and Acute Myocardial Infarction	NCT02742103
65	Boehringer Ingelheim / Evaluation of Dual Therapy With Dabigatran vs. Triple Therapy With Warfarin in Patients With AF That Undergo a PCI With Stenting (REDUAL-PCI)	NCT02164864
66	Dorbala et al. / Integrated Dual Exercise and Lexiscan Positron Emission Tomography: IDEALPET	NCT01109992
67	Ray et al. / Trial to Evaluate the Effect of ALN-PCSSC Treatment on Low Density Lipoprotein Cholesterol (LDL-C)	NCT02597127
68	Novartis Pharmaceuticals / Description of Tolerability of LCZ696 (Sacubitril / Valsartan) in Heart Failure With Reduced Ejection Fraction (HFrEF) Treated in Real Life Setting	NCT02690974
69	Kocher et al. / The Effects of Intravenous Heme Arginate on Heme Oxygenase-1 Expression (HO-1) and Oxidative Stress in the Human Heart	NCT02314780
70	Sanabria et al. / CiPA Phase 1 ECG Biomarker Validation Study	NCT03070470
71	Sarzynski et al. / Effects of Short-term Curcumin and Multi-polyphenol Supplementation on the Anti-inflammatory Properties of HDL	NCT02998918
72	Saban et al. / Mindful Hearts Study: Mindfulness to Reduce Stress	NCT01784796
73	Gaalema et al. / Increasing Cardiac Rehabilitation Participation Among Medicaid Enrollees	NCT02172820
74	Krucoff et al. / HARMONEE - Japan-USA Harmonized Assessment by Randomized, Multi-Center Study of OrbusNEich's Combo StEnt	NCT02073565
75	Bayer et al. / Rivaroxaban for the Prevention of Major Cardiovascular Events in Coronary or Peripheral Artery Disease	NCT01776424
76	Waymack et al. / Evaluation of Celecoxib Effects on Amlodipine in Subjects With Existing Hypertension Requiring Antihypertensives	NCT02979197
77	Viteri et al. / ToRsemide for pOstpartum HYpertension (TROPHY)	NCT02813551
78	Seto et al. / Comparing TR Band to Statseal in Conjunction With TR Band	NCT03028025
79	Choudhry et al. / Tele-Pharmacy Intervention to Improve Treatment Adherence	NCT02512276

© 2025 Rivera FB et al. *JAMA Network Open*.

80	Davies et al. / Objective Randomised Blinded Investigation With Optimal Medical Therapy of Angioplasty in Stable Angina	NCT02062593
81	Allen et al. / PCORI-1310-06998 Trial of a Decision Support Intervention for Patients and Caregivers Offered Destination Therapy Heart Assist Device	NCT02344576
82	Linder et al. / Forced Aerobic Exercise for Stroke Rehabilitation	NCT02494518
83	Sharma et al. / Auto-PAP for Pulmonary Hypertension Treatment in Decompensated HF Patients With Sleep Apnea.	NCT02963597
84	Carroll et al. / Short-Term Application of Tocilizumab Following Myocardial Infarction	NCT02419937
85	Hanrahan et al. / Trial of IW-1973, A Stimulator of Soluble Guanylate Cyclase (sGC) in Patients With Stable Type 2 Diabetes and Hypertension	NCT03091920
86	GSK Clinical Trials / A Phase 1 Relative Bioavailability Study of Ambrisentan and Tadalfil Fixed Dose Combination Tablets in Healthy Subjects	NCT02688387
87	Novartis Pharmaceuticals / Safety and Efficacy Study of LHW090 in Resistant Hypertension Patients	NCT02515331
88	Reid et al. / Sedentary Intervention Trial in Cardiac Rehabilitation	NCT02821962
89	Borlaug et al. / Inhaled Beta-adrenergic Agonists to Treat Pulmonary Vascular Disease in Heart Failure With Preserved EF (BEAT HFpEF): A Randomized Controlled Trial	NCT02885636
90	Reddy et al. / REnal Sympathetic dEnervaTion as an a Adjunct to Catheter-based VT Ablation	NCT01858194
91	Filippatos et al. / Effect of Elamipretide on Left Ventricular Function in Subjects With Stable Heart Failure With Reduced Ejection Fraction	NCT02788747
92	Zha et al. / Improve Hypertension Monitoring and Self-management by Using mHealth	NCT02632838
93	Leon et al. / Renal Denervation Using the Vessix Renal Denervation System for the Treatment of Hypertension (REDUCE HTN:REINFORCE)	NCT02392351
94	Lutsey et al. / Magnesium Supplementation for the Prevention of Supraventricular Arrhythmias	NCT02837328
95	Takeda et al. / Azilsartan Medoxomil (TAK-491) Compared to Valsartan in Chinese Participants With Hypertension	NCT02480764
96	Oh et al. / Temporary Autonomic Blockade to Prevent Atrial Fibrillation After Cardiac Surgery	NCT02498769
97	Katz et al. / Occult Paroxysmal Atrial Fibrillation in Non-Cryptogenic Ischemic Stroke	NCT02232022
98	Weisbord et al. / Prevention of Serious Adverse Events Following Angiography	NCT01467466
99	Gregory et al. / Power Training Post-stroke	NCT01970592
100	Kasner et al. / Improving Coordination and Transitions of Care in Stroke Patients	NCT02642744
101	Britto et al. / Effects of Cardiac Rehabilitation (CR) on Functional Capacity and Cardiovascular Risk Factors	NCT02575976
102	Angiolillo et al. / Switching From Ticagrelor to Clopidogrel in Patients With Coronary Artery Disease	NCT02287909
103	DeSouza et al. / Nebivolol and Endothelial Regulation of Fibrinolysis (NERF)	NCT01595516
104	Chalasani et al. / Clinical Trial to Evaluation the Safety and Efficacy of GR-MD-02 for the Treatment of Liver Fibrosis and Resultant Portal Hypertension in Patients With Nash Cirrhosis	NCT02462967
105	Pulia et al. / Effects of Oropharyngeal Strengthening on Dysphagia in Patients Post- stroke	NCT02322411
106	McCarthy et al. / ABLATE Post Approval Study - Synergy Ablation Lesions for Non- Paroxysmal Atrial Fibrillation	NCT01694563

© 2025 Rivera FB et al. *JAMA Network Open*.

107	Wang et al. / Affordability and Real-world Antiplatelet Treatment Effectiveness After Myocardial Infarction Study	NCT02406677
108	Perchenet et al. / PORtopulmonary Hypertension Treatment wIth maCitentan - a randOmized Clinical Trial	NCT02382016
109	Feld et al. / Evaluation of the Amigo Robotic System for Ablation of the Cavo-Tricuspid Isthmus	NCT02467179
110	Massachusetts General Hospital / Effects of Sublingual and Transdermal Administration of Nitroglycerin for Coronary CT Angiography on Image Quality	NCT02961946
111	Eiger BioPharmaceuticals / A Study of Ubenimex in Patients With Pulmonary Arterial Hypertension (WHO Group 1)	NCT02664558
112	MedImmune LLC / Multiple Ascending Doses of MEDI6012 in Subjects With Stable Atherosclerotic Cardiovascular Disease	NCT03004638
113	Dolan et al. / Evaluation of Safety and Efficacy of Lumason/SonoVue in Subjects Undergoing Pharmacologic Stress BR1-141	NCT02522481
114	Hernandez et al. / Subcutaneous Furosemide in Acute Decompensated Heart Failure Pilot	NCT02877095
115	Levine et al. / Chronotropic Incompetence in Patients With HFpEF	NCT02524145
116	Oral et al. / Use of Protamine for Heparin Reversal After Catheter Ablation of Atrial Fibrillation	NCT03140631
117	Grünig et al. / Early Treatment of Borderline Pulmonary Arterial Hypertension Associated With Systemic Sclerosis (SSc-APAH)	NCT02290613
118	Han et al. / Targeting Right Ventricle in Pulmonary Hypertension Gilead	NCT02829034
119	Wang et al. / Pragmatic Airway Resuscitation Trial	NCT02419573
120	PhaseBio Pharmaceuticals Inc. / Study to Assess the Safety, Tolerability and PK/PD After 4 Weekly SC Injections of PB1046 in Subjects With Stable HFrEF	NCT02808585
121	Boehringer Ingelheim / Safety and Efficacy of Alteplase When Administered in Chinese Patients With Acute Ischemic Hemispheric Stroke Where Thrombolysis is Initiated Between 3 and 4.5 Hours After Stroke Onset	NCT02930837
122	Hernandez et al. / Inorganic Nitrite Delivery to Improve Exercise Capacity in HFpEF	NCT02742129
123	Hernandez et al. / Stand & Move at Work	NCT02566317
124	Galinier et al. / Optimization of the Ambulatory Monitoring for Patients With Heart Failure by Tele-cardiology	NCT02068118
125	Howard et al. / IV Iron Replacement for Iron Deficiency in Idiopathic Pulmonary Arterial Hypertension (IPAH) Patients	NCT01447628
126	Novartis Pharmaceuticals / Safety and Tolerability During Open-label Treatment With LCZ696 in Patients With CHF and Reduced Ejection Fraction	NCT02226120
127	Piccini et al. / Genetically Targeted Therapy for the Prevention of Symptomatic Atrial Fibrillation in Patients With Heart Failure	NCT01970501
128	Gary et al. / Exercise and Cognitive Retraining to Improve Cognition in Heart Failure.	NCT02151266
129	Packer et al. / Catheter Ablation vs Anti-arrhythmic Drug Therapy for Atrial Fibrillation Trial	NCT00911508
130	Reiffel et al. / REVEAL AF: Incidence of AF in High Risk Patients	NCT01727297
131	Verheye et al. / Elixir Medical Evaluation of the DESolve® Novolimus Eluting Bioresorbable Coronary Scaffold System - Cx Registry	NCT04034121
132	Pokushalov et al. / DURABLE-I Study: Dielectric Unravelling of Radiofrequency ABLation Effectiveness	NCT02878213
133	Price et al. / Medtronic Resolute Onyx 2.0 mm Clinical Study	NCT02412501
134	Mehra et al. / Influence of Sleep Apnea on Risk of Atrial Fibrillation	NCT02576587
135	Medtronic Cardiac Rhythm and Heart Failure / Micra Accelerometer Sensor	NCT02930980

© 2025 Rivera FB et al. *JAMA Network Open*.

	Study 2	
136	Willems et al. / Utilizing Novel Dipole Density Capabilities to Objectively Visualize the Etiology of Rhythms in Atrial Fibrillation	NCT02825992
137	Clancy et al. / Bridge Occlusion Balloon in Lead Extraction Procedure	NCT02714153
138	Zaidat et al. / Analysis of Revascularization in Ischemic Stroke With EmboTrap	NCT02488915
139	Wells et al. / Vertebral Artery and Cerebral Hemodynamics After Various Head Positions & Manipulation in Patients With Neck Pain	NCT02667821
140	Huang et al. / SMART China, A Multi-center Clinical Registry Study	NCT02485925
141	Oxford University Hospitals NHS Trust / The Oxford Optimisation of PCI Study (OXOPT-PCI Study)	NCT03111940
142	Kandzari et al. / Sapphire II PRO US Clinical Study	NCT03052530
143	Stone et al. / Evaluation of Intracoronary Hyperoxemic Oxygen Therapy in Anterior Acute Myocardial Infarction Patients (IC-HOT)	NCT02603835
144	Mansourati et al. / Clinical Evaluation of LEft VEntricular Auto Threshold Algorithm (LEVEA)	NCT03014180
145	Fadol et al. / REcovery of Left Ventricular Dysfunction in CAncer Patients (RECAP Trial)	NCT02543294
146	Simon et al. / Inhaled Nitrite in Subjects With Pulmonary Hypertension	NCT01431313
147	Ginsburg et al. / Family Health History in Diverse Care Settings (FHH)	NCT01956773
148	Levine et al. / Chronotropic Incompetence in Patients With HFpEF	NCT02524145
149	Knight et al. / Sustained Treatment of Paroxysmal Atrial Fibrillation Post-Approval Study (STOP AF PAS)	NCT01456949
150	Sticherling et al. / BIO|MASTER.Ilivia Family / Plexa	NCT02774616
151	Shin et al. / Effects of a New Dispatcher-Assisted Basic Life Support Training Program	NCT02142387
152	Shin et al. / Dispatcher-Activated Neighborhood Access Defibrillation and Cardiopulmonary Resuscitation	NCT02010151
153	Kim et al. / SJM MRI Diagnostic Imaging Registry (IDE)	NCT02807948
154	Goldstein et al. / Prospective, Double Blind, Placebo Control, Bariatric IV Ace	NCT02452320
155	Pagoto et al. / Feasibility Trial of a Problem-Solving Weight Loss Mobile Application	NCT02192905
156	Camilleri et al. / Ursodiol on Insulin Sensitivity, Gastric Emptying and Body Weight With Type 2 Diabetes on Metformin	NCT02033876
157	Long et al. / A Multi-center Trial of IMPaCT CHW Support for Chronically-ill Patients	NCT02347787
158	Cook et al. / Weight Loss Using the Take Shape For Life Program or the Medifast Direct Program Versus a Self-Directed Diet	NCT02835092
159	Rosenberg et al. / I-STAND R21: Reducing Sedentary Time in Older Adults	NCT02692560
160	Stumvoll et al. / A Multiple-ascending-dose Study to Evaluate the Efficacy, Safety, and Pharmacokinetics (PK) of MEDI0382 in Overweight and Obese Participants With Type 2 Diabetes Mellitus	NCT02548585
161	Krukowski et al. / Dissemination of the Look Ahead Weight Management Treatment in the Military	NCT02063178
162	University of Miami / Effect of Liraglutide on Epicardial Fat in Subjects With Type 2 Diabetes	NCT02014740
163	McCarthy et al. / Is MyPlate Approach to Helping Overweight Patients Lose Weight More Patient-centered?	NCT02514889
164	Beavers et al. / Effect of High Protein Weight Loss for Seniors	NCT02730988

© 2025 Rivera FB et al. *JAMA Network Open*.

165	Novo Nordisk A/S / Investigation of Safety and Efficacy of Once-daily Semaglutide in Obese Subjects Without Diabetes Mellitus	NCT02453711
166	Hu et al. / A Short Term Evaluation of a Structured Weight Loss Plan in Overweight and Obese Adults	NCT02774668
167	Devin et al. / Effect of DPP4 Inhibition on Growth Hormone Secretion	NCT01701973
168	Lee et al. / A Study to Evaluate the Efficacy and Safety of Cyclo-Z in Patients With Obese Type 2 Diabetes	NCT02784275
169	Venditti et al. / The Pitt Retiree Study: A Diabetes Prevention Program for Medicare Eligible Older Adults	NCT03192475
170	Monk et al. / Surgical Conditions During Laparoscopic Bariatric Surgery	NCT02703909
171	Jay et al. / Improving Weight Management at the VA	NCT02626819
172	Wadden et al. / Lifestyle Modification and Lorcaserin for Weight Loss Maintenance	NCT02388568
173	Shah et al. / Fish Oils and Adipose Inflammation Reduction	NCT02010359
174	Rose et al. / Promoting Gastrointestinal Health and Reducing Subclinical Inflammation in Obese Individuals	NCT02602496
175	Heshmati et al. / Study of Gelesis100 on Body Weight in Overweight and Obese Subjects With and Without Type 2 Diabetes	NCT02307279
176	GSK Clinical Trials / Safety, Tolerability and Preliminary Pharmacokinetics (PK) and Pharmacodynamics (PD) of Single and Repeat Oral Doses of GSK3008356 in Healthy and Obese Subjects	NCT02742766
177	Vitolins et al. / Latinos Combating Diabetes	NCT01831921
178	Van Horn et al. / Maternal-Offspring Metabolics:Family Intervention Trial	NCT01631747
179	Garcia et al. / Feasibility and Acceptability of a Beverage Intervention for Hispanic Adults	NCT02911753
180	Fox et al. / High-Flow Nasal Cannula and Desaturation Episodes in the Morbidly Obese Patients	NCT03148262
181	Novartis Pharmaceuticals / Effects of Carbohydrate in Diet and Supplements on the Gastrointestinal Tolerability of LIK066	NCT03198767
182	Spring et al. / Opt-IN: Optimization of Remotely Delivered Intensive Lifestyle Treatment for Obesity	NCT01814072
183	Kohrt et al. / Females, Aging, Metabolism, and Exercise	NCT01712230
184	Shibao et al. / Racial Differences in Vagal Control of Glucose Homeostasis	NCT02365285
185	Acosta et al. / Ox Bile- Conjugated Bile Acids Sodium in Type II Diabetes Mellitus	NCT02871882
186	Sherwood et al. / The SIM-PLICITY Study: The SIMulation Project - LIstening & Intervention in Pediatric obeSITY	NCT02946515
187	Schroeder et al. / Comparison of Outreach Methods to Encourage Enrollment in Diabetes Prevention and Weight Management Programs	NCT03200535
188	University of Pennsylvania / The Weigh Forward, Financial Incentives for Maintenance of Weight Loss	NCT02538783
189	Gudzune et al. / Sugar Champ: Pilot Social Network Intervention to Reduce Intake of Sugary Drinks	NCT02138240
190	Rothstein et al. / ENDObesity® II Study: TransPyloric Shuttle® System for Weight Loss	NCT02518685
191	Weiss et al. / Bariatric Embolization of Arteries for the Treatment of Obesity	NCT02165124
192	Joslin Diabetes Center / Metabolic Effects of Betaine Supplementtion	NCT01950039
193	Buman et al. / Stand & Move at Work	NCT02566317

194	Boehringer Ingelheim / Empagliflozin and ACEi- Effects on Hyperfiltration: BETWEEN Study	NCT02632747
195	Campbell et al. / Pilot Behavioral Support Intervention After Bariatric Surgery	NCT03092479
196	Singh et al. / CRT Implant Strategy Using the Longest Electrical Delay for Non-left Bundle Branch Block Patients (ENHANCE CRT)	NCT01983293
197	Won et al. / The Effects of Swimming on Elderly Women With Stage-2 Hypertension	NCT03546270
198	Han et al. / Targeting the Right Ventricle in Pulmonary Hypertension	NCT01839110
199	Ballantyne et al. / Evaluation of the Efficacy and Safety of Bempedoic Acid (ETC- 1002) as Add-on to Ezetimibe Therapy in Patients With Elevated LDL-C (CLEAR Tranquility)	NCT03001076
200	Horowitz et al. / Genetic Testing to Understand and Address Renal Disease Disparities (GUARDD)	NCT02234063
201	Bateman et al. / Characterization of Changes in Ventricular Mechanics in Response to Lexiscan Stress Using Tagged Cine Cardiac Magnetic Resonance Imaging	NCT02115308
202	Rosenstock et al. / Cardiovascular and Renal Microvascular Outcome Study With Linagliptin in Patients With Type 2 Diabetes Mellitus (CARMELINA)	NCT01897532
203	Bardoxolone Methyl Evaluation in Patients With Pulmonary Hypertension (PH) - LARIAT	NCT02036970
204	Schwartz et al. / ODYSSEY Outcomes: Evaluation of Cardiovascular Outcomes After an Acute Coronary Syndrome During Treatment With Alirocumab	NCT01663402
205	Marian et al. / Crushed Ticagrelor Versus Eptifibatide Bolus + Clopidogrel	NCT02925923
206	Tien Ng et al. / Aquaresis Utility for Hyponatremic Acute Heart Failure Study	NCT02183792
207	Aonuma et al. / A Study to Evaluate the Safety and Effectiveness of the Left Atrial Appendage Closure Therapy Using BSJ003W (SALUTE)	NCT03033134
208	FitzGerald et al. / Testing of a Valsalva Assist Device (VAD) in Healthy Volunteers Performing a Valsalva Manoeuvre	NCT03298880
209	Gladwin et al. / Oral Nitrite in Adults With Metabolic Syndrome and Hypertension	NCT01681810
210	Hernandez et al. / Effect of Albiglutide, When Added to Standard Blood Glucose Lowering Therapies, on Major Cardiovascular Events in Subjects With Type 2 Diabetes Mellitus	NCT02465515
211	Wachter et al. / Comparison of Pre- and Post-discharge Initiation of LCZ696 Therapy in HFrEF Patients After an Acute Decompensation Event (TRANSITION)	NCT02661217
212	Schoenthaler et al. / Tailored Approaches to Improve Medication Adherence	NCT01643473
213	Ray et al. / Evaluation of Long-Term Safety and Tolerability of ETC-1002 in High- Risk Patients With Hyperlipidemia and High CV Risk (CLEAR Harmony)	NCT02666664
214	Schaffer et al. / Lipid Biomarkers for Diabetic Heart Disease	NCT01752842
215	Ho et al. / Hybrid Effectiveness-Implementation Study to Improve Clopidogrel Adherence	NCT01609842
216	Crimm et al. / Focused Field of View Calcium Scoring Prior to Coronary CT Angiography (FOCUS-CCTA)	NCT02972242
217	Koh et al. / Microvascular Assessment of Ranolazine in Non-Obstructive Atherosclerosis (MARINA) (MARINA)	NCT02147067
218	Lee et al. / Taekwondo Training and Postmenopausal Women With Stage-2 Hypertension	NCT03544307
219	Tran et al. / Noninvasive Neuromodulation to Reserve Diastolic Dysfunction (NERDD)	NCT02983448

© 2025 Rivera FB et al. *JAMA Network Open*.

220	Femoral Vein Hemostasis After Ablation for Atrial Fibrillation With Manual Pressure Versus a Figure of 8 Suture (Figure 8)	NCT03040661
221	Trankle et al. / Vitamin C in Atrial Fibrillation Ablation (VitC-AF)	NCT03148236
222	Laufs et al. / Evaluation of the Efficacy and Safety of Bempedoic Acid (ETC-1002) in Patients With Hyperlipidemia and Statin Intolerant (CLEAR Serenity)	NCT02988115
223	Khandwalla et al. / Study on the Effects of Sacubitril/Valsartan on Physical Activity and Sleep in Heart Failure With Reduced Ejection Fraction Patients. (AWAKE-HF)	NCT02970669
224	Hobbs et al. / A Prospective Evaluation of Natriuretic Peptide Based Referral of CHF Patients in Primary Care	NCT02807857
225	Voors et al. / A Trial to Study Neladenoson Bialanate Over 20 Weeks in Patients With Chronic Heart Failure With Reduced Ejection Fraction (PANTHEON)	NCT02992288
226	Ortiz et al. / Effect of Dapagliflozin on Blood Pressure Variability in Prediabetes and Prehypertension	NCT03006471
227	Langen et al. / The Effect of Ibuprofen on Post-partum Blood Pressure in Women With Hypertensive Disorders of Pregnancy	NCT02891174
228	Schopfer et al. / The Healthy Heart Study	NCT02105246
229	Di Santo et al. / Utility of a Smart Phone Application in Assessing Radial Artery Patency - the CAPITAL iRADIAL Study	NCT02519491
230	Soetisna et al. / Transepicardial With Transseptal Autologous CD 133+ Bone Marrow Cell Implantation in Patient Following CABG Surgery	NCT02870933
231	Rivaroxaban Versus Warfarin in the Evaluation of Progression of Coronary Calcium	NCT02376010
232	Reed et al. / The IPED (Investigation of Palpitations in the ED) Study (IPED)	NCT02783898
233	Velikonja et al. / Impact of Consumption of Beta-glucans on the Intestinal Microbiota and Glucose and Lipid Metabolism	NCT02041104
234	Piepoli et al. / randOmized stUdy Using accelerometTry to Compare Sacubitril/valsarTan and Enalapril in Patients With Heart Failure (OUTSTEP-HF)	NCT02900378
235	Ono et al. / Clinical Pharmacology Trial to Investigate the Dose of OPC-61815 Injection Equivalent to Tolvaptan 15-mg Tablet in Patients With Congestive Heart Failure	NCT03254108
236	Bhatt et al. / A Study of AMR101 to Evaluate Its Ability to Reduce Cardiovascular Events in High-Risk Patients With Hypertriglyceridemia and on Statin (REDUCE- IT)	NCT01492361
237	Alsouqi et al. / Tissue Sodium in Pre-hypertensive Patients	NCT02236520
238	Catino et al. / Efficacy of Optison Echo Contrast to Detect Thrombus in Left Atrial Appendage	NCT01721447
239	Rosenson et al. / Comparative Efficacy of Ticagrelor Versus Aspirin on Blood Viscosity in Peripheral Artery Disease Patients With Type 2 Diabetes	NCT02325466
240	Abraham et al. / C-Pulse IDE Feasibility Study- A Heart Assist System	NCT00815880
241	Bohula et al. / A Study to Evaluate the Effect of Long-term Treatment With BELVIQ (Lorcaserin HCl) on the Incidence of Major Adverse Cardiovascular Events and Conversion to Type 2 Diabetes Mellitus in Obese and Overweight Subjects With Cardiovascular Disease or Multiple Cardiovascular Risk Factors (CAMELLIA- TIMI)	NCT02019264
242	Multicenter Study to Evaluate the Procedural Safety and Efficacy of ELCA in CAD	NCT03284229
243	Shah et al. / A Trial to Study Neladenoson Bialanate Over 20 Weeks in Patients With Chronic Heart Failure With Preserved Ejection Fraction (PANACHE)	NCT03098979
244	Scalzo et al. / Impact of Sitagliptin on Cardiovascular Exercise Performance in Type 2 Diabetes	NCT01951339
245	Laufs et al. / Pharmacy-based Interdisciplinary Program for Patients With Chronic Heart Failure (PHARM-CHF)	NCT01692119

© 2025 Rivera FB et al. *JAMA Network Open*.

246	Ballantyne et al. / A Study Evaluating the Safety and Efficacy of Bempedoic Acid Plus Ezetimibe Fixed-Dose Combination Compared to Bempedoic Acid, Ezetimibe, and Placebo in Patients Treated With Maximally Tolerated Statin Therapy	NCT03337308
247	White et al. / Phase III Clinical Worsening Study of UT-15C in Subjects With PAH Receiving Background Oral Monotherapy (FREEDOM-EV)	NCT01560624
248	Rosenfield et al. / Renal Stent Placement for the Treatment of Renal Artery Stenosis in Patients With Resistant Hypertension (ARTISAN)	NCT01673373
249	Velasquez et al. / Comparison of Sacubitril/Valsartan Versus Enalapril on Effect on NT-proBNP in Patients Stabilized From an Acute Heart Failure Episode. (PIONEER- HF)	NCT02554890
250	Witte et al. / Carillon Mitral Contour System® for Reducing Functional Mitral Regurgitation (REDUCE FMR)	NCT02325830
251	Mullen et al. / Connected Health Blood Pressure Monitoring In Stroke and TIA Patients (CHAMPS)	NCT02450760
252	Kwon et al. / Preventive Effects of Ginseng Against Atherosclerosis (PEGASUS)	NCT02796664
253	Huffman et al. / Positive Psychology for Acute Coronary Syndrome Patients (PEACE-IV)	NCT03122184
254	Ali et al. / INtegrating DEPrEssioN and Diabetes treatmENT (INDEPENDENT) Study (INDEPENDENT)	NCT02022111
255	Adlam et al. / A Study of Acute Myocardial Infarction Using FDY-5301	NCT03470441
256	Tsimikas et al. / Phase 2 Study of ISIS 681257 (AKCEA-APO(a)-LRx) in Participants With Hyperlipoproteinemia(a) and Cardiovascular Disease	NCT03070782
257	Ako et al. / Evaluation of Effect of Alirocumab on Coronary Atheroma Volume in Japanese Patients Hospitalized for Acute Coronary Syndrome With Hypercholesterolemia (ODYSSEY J-IVUS)	NCT02984982
258	Kronish et al. / Comparison of Depression Identification After Acute Coronary Syndrome: Quality of Life and Cost Outcomes (CODIACSQoL)	NCT01993017
259	Lock et al. / Bexagliflozin Efficacy and Safety Trial (BEST)	NCT02558296
260	Gaffo et al. / Center of Research Translation (CORT) Project 2	NCT02038179
261	Trankle et al. / Alirocumab in Patients With Acute Myocardial Infarction	NCT02938949
262	Han et al. / Evaluation of Alirocumab Versus Ezetimibe on Top of Statin in Asia in High Cardiovascular Risk Patients With Hypercholesterolemia (ODYSSEY EAST)	NCT02715726
263	Frias et al. / Usability Study of the Commercial Auto-injector Device and the New Auto-injector Device (SYDNEY) in Patients With High or Very High CV Risk With Hypercholesterolemia Not Adequately Controlled With Their Lipid-Modifying Therapy	NCT03415178
264	Yau et al. / Safety & Efficacy of Intramyocardial Injection of Mesenchymal Precursor Cells on Myocardial Function in LVAD Recipients	NCT02362646
265	Carubelli et al. / The Clinical Study of the Safety and Efficacy of Istaroxime in Treatment of Acute Decompensated Heart Failure	NCT02617446
266	Diener et al. / Dabigatran Etexilate for Secondary Stroke Prevention in Patients With Embolic Stroke of Undetermined Source (RE-SPECT ESUS)	NCT02239120
267	Storey et al. / A Medical Research Study to Evaluate the Effects of ACT-246475 in Adults With Coronary Artery Disease	NCT03384966
268	Gerstein et al. / Researching Cardiovascular Events With a Weekly Incretin in Diabetes (REWIND) (REWIND)	NCT01394952
269	Rosenstock et al. / CAROLINA: Cardiovascular Outcome Study of Linagliptin Versus Glimepiride in Patients With Type 2 Diabetes	NCT01243424
270	Goldberg et al. / Evaluation of Long-Term Efficacy of Bempedoic Acid (ETC-1002) in Patients With Hyperlipidemia at High Cardiovascular Risk (CLEAR	NCT02991118

© 2025 Rivera FB et al. *JAMA Network Open*.

	Wisdom)	
271	Noc et al. / COOL AMI EU Pivotal Trial to Assess Cooling as an Adjunctive Therapy to PCI In Patients With Acute MI (Phase A)	NCT03173313
272	Al-Ali et al. / The Capillary Index Score Trial	NCT02618031
273	Renoprotective Effects of Dapagliflozin in Type 2 Diabetes (RED)	NCT02682563
274	Abbate et al. / Treatment of Diabetes in Patients With Systolic Heart Failure	NCT02920918
275	Hohnloser et al. / Edoxaban Treatment Versus Vitamin K Antagonist (VKA) in Patients With Atrial Fibrillation (AF) Undergoing Catheter Ablation (ELIMINATE- AF)	NCT02942576
276	Husain et al. / A Trial Investigating the Cardiovascular Safety of Oral Semaglutide in Subjects With Type 2 Diabetes (PIONEER 6)	NCT02692716
277	Cox et al. / Comparison of Oral or Intravenous Thiazides vs Tolvaptan in Diuretic Resistant Decompensated Heart Failure	NCT02606253
278	Mehra et al. / MOMENTUM 3 IDE Clinical Study Protocol (HM3™)	NCT02224755
279	White et al. / Intensive Versus Standard Blood Pressure Lowering to Prevent Functional Decline in Older People	NCT01650402
280	Vellone et al. / MOTIVATional intErviewing to Improve Self-care in Heart Failure Patients (MOTIVATE-HF)	NCT02894502
281	Oyanguren et al. / Safety and Effectiveness of Drug up Titration by Nurses Specialized in Heart Failure (HF) Patients (ETIFIC)	NCT02546856
282	Toscos et al. / Keep it SIMPLE: Improving Anti-Coagulation Medication Adherence	NCT02690649
283	Kirstein et al. / A Feasibility Study to Evaluate the Effect of Concomitant Renal Denervation and Cardiac Ablation on AF Recurrence (RDN+AF)	NCT01907828
284	Mentz et al. / Predischarge Initiation of Ivabradine in the Management of Heart Failure (PRIME-HF) (PRIME-HF)	NCT02827500
285	ENABLE CHF-PC (Comprehensive Heartcare For Patients and Caregivers) (ENABLE CHF PC)	NCT02505425
286	Perkovic et al. / Evaluation of the Effects of Canagliflozin on Renal and Cardiovascular Outcomes in Participants With Diabetic Nephropathy (CREDENCE)	NCT02065791
287	Muliawan et al. / The Role of Trimetazidine on Right Ventricle Function in Pulmonary Arterial Hypertension (TRIMETA-PH)	NCT03273387
288	Humphries et al. / Integrated Management Program Advancing Community Treatment of Atrial Fibrillation (IMPACT-AF)	NCT01927367
289	Ruff et al. / A Study to Evaluate the Safety, Pharmacokinetics, and Pharmacodynamic Effects of MEDI5884 in Adults With Stable Coronary Heart Disease	NCT03351738
290	Lopes et al. / A Study of Apixaban in Patients With Atrial Fibrillation, Not Caused by a Heart Valve Problem, Who Are at Risk for Thrombosis (Blood Clots) Due to Having Had a Recent Coronary Event, Such as a Heart Attack or a Procedure to Open the Vessels of the Heart	NCT02415400
291	Brachmann et al. / Randomized Evaluation of Atrial Fibrillation Treatment With Focal Impulse and Rotor Modulation Guided Procedures (REAFFIRM)	NCT02274857
292	Sinnaeve et al. / A Medical Research Study to Evaluate the Effects of ACT-246475 in Adults With Heart Attack	NCT03487445
293	Ding et al. / Genomic Outcomes of Metformin (GOMET)	NCT02986659
294	Allegretti et al. / An Integrated Assessment of the Safety and Effectiveness of Bexagliflozin for the Management of Essential Hypertension	NCT03514641
295	Bisognano et al. / Conditioned Pharmacotherapeutic Effects in Hypertension	NCT02056626

© 2025 Rivera FB et al. *JAMA Network Open*.

296	Buck et al. / Post-Embolic Rhythm Detection With Implantable Versus External Monitoring (PERDIEM)	NCT02428140
297	St-Onge et al. / Active Clearance of Chest Tubes After Cardiac Surgery: A Prospective Randomized Controlled Study	NCT02808897
298	Manoukian et al. / Shortened Aggrastat® Versus Integrilin in Percutaneous Coronary Intervention (SAVI-PCI)	NCT01522417
299	AMPLATZER Cardiac Plug Clinical Trial	NCT01118299
300	Mordi et al. / SGLT2 Inhibition in Combination With Diuretics in Heart Failure (RECEDE-CHF)	NCT03226457
301	Ronsein et al. / Evaluate Carotid Artery Plaque Composition by Magnetic Resonance Imaging in People Receiving Cholesterol Medication (CPC)	NCT00715273
302	Desai et al. / Study of Effects of Sacubitril/Valsartan vs. Enalapril on Aortic Stiffness in Patients With Mild to Moderate HF With Reduced Ejection Fraction (EVALUATE-HF)	NCT02874794
303	Franchi et al. / Platelet Inhibition With Cangrelor and Crushed Ticagrelor in STEMI (CANTIC)	NCT03247738
304	Abbate et al. / Interleukin-1 (IL-1) Blockade in Acute Myocardial Infarction (VCU-ART3) (VCU-ART3)	NCT01950299
305	Gasecka et al. / Antiplatelet Therapy Effect on Extracellular Vesicles in Acute Myocardial Infarction (AFFECT EV)	NCT02931045
306	Neeland et al. / Visceral Adiposity and Diabetes: Translating Form to Function Using Imaging	NCT02833415
307	Downs et al. / Healthy Mom Zone: A Gestational Weight Gain Management Intervention (HMZ)	NCT03945266
308	Methodology Study To Examine 6-Week Food Intake With Liraglutide In Obese Subjects	NCT03041792
309	Ma et al. / Research Aimed at Improving Both Mood and Weight (RAINBOW-ENGAGE)	NCT02246413
310	Parker et al. / A Study to Investigate Different Doses of 0382 in Overweight and Obese Subjects With Type 2 Diabetes Mellitus.	NCT03244800
311	Kaviani et al. / Daily Self-weighing and Holiday-associated Weight Gain in Adults	NCT03718000
312	Hughes et al. / Fit and Strong! Plus Comparative Effectiveness Trial	NCT03180008
313	Cabassa et al. / Peer-Led Healthy Lifestyle Program in Supportive Housing	NCT02175641
314	Dandona et al. / The Effect of LINAGLIPTIN on Inflammation, Oxidative Stress and Insulin Resistance in Obese Type 2 Diabetes Subjects (1971)	NCT02372630
315	Thompson et al. / Helping Moms to be Healthy After Baby	NCT03257657
316	McVay et al. / Increasing Uptake of Behavioral Weight Loss Programs Among Primary Care Patients	NCT02708121
317	Myers et al. / Location-based Smartphone Technology to Guide College Students Healthy Choices Ph II	NCT02996864
318	Ramachandran et al. / Effect of Vestibular Stimulation on Fat Consumption and Energy Expenditure as Assessed Using Indirect Calorimetry	NCT03138382
319	Bays et al. / A Study to Evaluate the Change in Weight After 24 Weeks Treatment With LIK066 in Obese or Overweight Adults	NCT03100058
320	Lindberg et al. / De Por Vida: A Diabetes Risk Reduction Intervention for Hispanic Women	NCT03113916
321	Perez et al. / Intrapartum Epidural Catheter Displacement: Dressing Methods	NCT03574441
322	Velikonja et al. / Impact of Consumption of Beta-glucans on the Intestinal Microbiota and Glucose and Lipid Metabolism	NCT02041104
323	Gedeon et al. / Use of Non-Invasive Positive Pressure Ventilation in Patients With Severe Obesity Undergoing Upper Endoscopy Procedures	NCT03529461
324	Braun et al. / Effects of Pitavastatin on Insulin Sensitivity and Liver Fat	NCT02290106

© 2025 Rivera FB et al. *JAMA Network Open*.

325	Voils et al. / Incentivizing Behavior Change Skills to Promote Weight Loss	NCT02691260
326	Nahra et al. / A Study to Evaluate the Efficacy and Safety of MEDI0382 in the Treatment of Overweight and Obese Subjects With Type 2 Diabetes	NCT03235050
327	Stinson et al. / Predicting Adherence to a Heart-Healthy Diet in Lean and Obese Individuals	NCT01862796
328	Wadden et al. / Effect and Safety of Liraglutide 3.0 mg as an Adjunct to Intensive Behaviour Therapy for Obesity in a Non-specialist Setting (SCALE™ IBT)	NCT02963935
329	Xiang et al. / Beta Cell Restoration Through Fat Mitigation (BetaFat)	NCT01763346
330	Apolzan et al. / The DEB Pilot Study (DEB Pilot)	NCT03190993
331	Voils et al. / Development of a Weight Maintenance Intervention for Bariatric Surgery Patients (MAINTAIN-B)	NCT03246672
332	Viteria et al. / Cervical Ripening for Obese Women: A Randomized, Comparative Effectiveness Trial (CROWN)	NCT02639429
333	Tan et al. / Study of Pharmacodynamics of LIK066 in Overweight and Obese Women With Polycystic Ovary Syndrome (PCOS)	NCT03152591
334	Yu et al. / Fecal Microbiota Transplant for Improvement of Metabolism (FMT-TRIM)	NCT02530385
335	Ard et al. / Qsymia as an Adjunct to Surgical Therapy in the Superobese	NCT02301416
336	Du et al. / Naltrexone and Bupropion Combination on Obese,Smoking Patients With Schizophrenia	NCT02736474
337	Mason et al. / Biology and Experience of Eating in Women With Obesity (BEE)	NCT02805972
338	Yokote et al. / A Dose-finding Study to Evaluate the Change in Weight After Treatment With LIK066 in Japanese Patients With Obesity	NCT03320941
339	Ramsden et al. / Dietary Essential Fatty Acid Regulation of Omega-3 HUFA Metabolism; Satiety and Body Composition	NCT01251887
340	Yancy et al. / Jump Start Shared Medical Appointments for Diabetes With Weight Management (Jump Start)	NCT01973972
341	Croghan et al. / LLLT for Reducing Waste Circumference and Weight (LLLT)	NCT02877004
342	Dushay et al. / Mechanisms of Weight Loss With SGLT2 Inhibition	NCT02360774
343	Rebello et al. / The Effect Of NS-0200 and NS-0300 Versus Placebo on Weight In Obesity	NCT03364335
344	Demidowich et al. / Effects of Colchicine in Non-Diabetic Adults With Metabolic Syndrome	NCT02153983
345	Garvey et al. / Effect and Safety of Liraglutide 3.0 mg in Subjects With Overweight or Obesity and Type 2 Diabetes Mellitus Treated With Basal Insulin (SCALE™ Insulin)	NCT02963922
346	Kang et al. / A First-in-human Study to Evaluate the Safety, Tolerability, Pharmacokinetics and Pharmacodynamics of HM15211	NCT03374241
347	Berger et al. / Use of High Flow Nasal Cannula During Sedation of Morbidly Obese Patients in the Endoscopy Suite	NCT02859597
348	Abu Dayyeh et al. / Spatz Adjustable Balloon for Obesity (SABO) (SABO)	NCT02812160
349	Powell et al. / Utility of Ultrasound in Identification of Midline and Placement of Epidural in Severely Obese Parturients	NCT03100968
350	Laroche et al. / Family Obesity Intervention: Motivational Interviewing and Community Support	NCT02425046
351	Wei et al. / Oxytocin Dosage to Decrease Induction Duration (OPS)	NCT03140488
352	Allegretti et al. / Fecal Microbiota Transplantation for the Treatment of Obesity (FMT Obesity)	NCT02741518
353	Unick et al. / Exercise as a Buffer Against Stress-induced Overeating	NCT02936076

© 2025 Rivera FB et al. *JAMA Network Open*.

354	Wadden et al. / Lifestyle Modification and Liraglutide (MODEL)	NCT02911818
355	Baer et al. / Integrating Online Weight Management With Primary Care Support (PROPS)	NCT02656693
356	Miller et al. / Study of Growth Hormone and Bone in Obesity	NCT01724489
357	Reddy et al. / The Lowering Weight in Severe Obesity by Embolization of the Gastric Artery Trial (LOSEIT)	NCT03185949
358	Bain et al. / Study of MEDI0382 in Combination With Dapagliflozin and Metformin in Overweight/Obese Participants With Type 2 Diabetes	NCT03444584
359	Espinoza et al. / The Physiologic Effects of Intranasal Oxytocin on Sarcopenic Obesity (INOSO)	NCT03119610
360	Bauer et al. / Obesity Prevention in Head Start	NCT03672227
361	Smyth et al. / Ketamine vs Hydromorphone	NCT03001843
362	Evaluation of Safety, Efficacy and Thermogenesis-induction of RZL-012 in Overweight and Obese Volunteers	NCT03171415
363	Pisegna et al. / Gastrointestinal Hormonal Regulation of Obesity	NCT01146704
364	Stote et al. / Blueberry Consumption and Type 2 Diabetes	NCT02972996
365	Klose et al. / Safety Study of the Switch From Oral Selexipag to Intravenous Selexipag in Subjects With Stable Pulmonary Arterial Hypertension	NCT03187678
366	Nepper et al. / Assessing Home Food Environment and Diabetes Self-management Among Adult Type 2 Diabetes Patients	NCT03039569
367	Mor-Avi et al. / Stress CT Perfusion in Patients With Chest Pain	NCT01969916
368	Use of Lexiscan for Myocardial Stress Perfusion Computed Tomography With a 3rd Generation Dual Source CT System	NCT03103061
369	Bliden et al. / Vorapaxar on Thrombin Generation and Coagulability	NCT03207451
370	Zareba et al. / Long-term Efficacy Study of Sodium Channel Blocker in LQT3 Patients	NCT01648205
371	Attain Stability™ Quad Clinical Study	NCT03099655
372	Ruffner et al. / TactiSense IDE Trial of TactiCath SE for Paroxysmal Atrial Fibrillation	NCT03354663
373	Clinical Study for Workflow and Performance Evaluation of the THERMOCOOL SMARTTOUCH® SF-5D System for Treating Symptomatic Paroxysmal AF (QDOT-Micro)	NCT02944968
374	Cené et al. / Patient and Family Partners in Heart Failure Care: Pilot Test of a Pre- visit Question Prompt List to Enhance Engagement During Medical Visits	NCT03491800
375	Liu et al. / Contrast ICE for Myocardial Scar in VT Ablations	NCT03212326
376	Han et al. / 11C-acetate/18Fluorodeoxyglucose-FDG PET/CT and Cardiac MRI in Pulmonary Hypertension	NCT01917136
377	Reddy et al. / Clinical Study for Safety and Acute Performance Evaluation of the THERMOCOOL SMARTTOUCH® SF-5D System Used With Fast Ablation Mode in Treatment of Patients With Paroxysmal Atrial Fibrillation.	NCT03459196
378	Januzzi et al. / Effects of Sacubitril/Valsartan Therapy on Biomarkers, Myocardial Remodeling and Outcomes.	NCT02887183
379	Franchi et al. / Vorapaxar as an Add-On Antiplatelet Therapy in Patients With and Without Diabetes Mellitus (OPTIMUS-5)	NCT02548650
380	Shi et al. / Efficacy and Safety of Low-dose Ticagrelor	NCT03381742
381	Reddy et al. / IMPULSE: A Safety and Feasibility Study of the IOWA Approach Endocardial Ablation System to Treat Atrial Fibrillation	NCT03700385
382	Bazerbachi et al. / EUS Guided Core Liver Biopsy and IGB Placement for the Diagnosis and Management of NASH and Obesity	NCT02880189

© 2025 Rivera FB et al. *JAMA Network Open*.

383	Tsymbal et al. / Oxygen Reserve Index: Utility as Early Warning for Desaturation in Morbidly Obese Patients	NCT03021551
384	Reddy et al. / REnal SympatheticC Denervation to sUpprEss Tachyarrhythmias in ICD Recipients	NCT01747837
385	Vitamin D, Insulin Resistance, and Cardiovascular Disease	NCT00736632
386	A Study of CLR325 in Chronic Stable Heart Failure Patients.	NCT02696967
387	Steg et al. / A Study Comparing Cardiovascular Effects of Ticagrelor Versus Placebo in Patients With Type 2 Diabetes Mellitus	NCT01991795
388	Gill et al. / Coronary Artery Disease Screening in Kidney Transplant Candidates	NCT02082483
389	Mahfoud et al. / The Peregrine Post-Market Study for the Treatment of Hypertension	NCT02570113
390	Schilling et al. / Multi-electrode Radiofrequency Balloon Catheter Use for the Isolation of the Pulmonary Veins.	NCT03437733
391	van Berkel et al. / Intraoperative Amiodarone to Prevent Atrial Fibrillation in Lung Transplant Patients	NCT03221764
392	Singh et al. / Multicenter Automatic Defibrillator Implantation Trial - Chemotherapy- Induced Cardiomyopathy	NCT02164721
393	Tsutsui et al. / Study of Efficacy and Safety of LCZ696 in Japanese Patients With Chronic Heart Failure and Reduced Ejection Fraction	NCT02468232
394	Sanz et al. / Quartet Lead and Resynchronization Therapy Options III (QUARTO_III)	NCT02476201
395	Oudiz et al. / Beraprost-314d Added-on to Tyvaso® (BEAT)	NCT01908699
396	Crandall et al. / Prevention of Non-Surgical Bleeding by Management of HeartMate II Patients Without Antiplatelet Therapy	NCT02836652
397	Perez et al. / Apple Heart Study: Assessment of Wristwatch-Based Photoplethysmography to Identify Cardiac Arrhythmias	NCT03335800
398	Frerichs et al. / Reducing Cardiovascular Disease Risk Factors in Rural Communities in North Carolina	NCT02707432
399	Jonas et al. / BIONICS 38 mm Trial EluNIR Ridaforolimus Eluting Coronary Stent System (EluNIR) In Coronary Stenosis Trial	NCT03702608
400	Bruce et al. / Integrated Tele-monitoring and Patient-centric Health Coaching Strategy in Patients Hospitalized With Heart Failure	NCT02391987
401	Li et al. / Human Urinary Kallidinogenase Improve Short Term Motor Functional Outcome of Acute Ischemia Stroke Patients	NCT04102956
402	Sylvia et al. / Healthy Hearts Healthy Minds	NCT03373110
403	Crozier et al. / Extravascular ICD Pilot Study	NCT03608670
404	Su et al. / Observation of ImageReady™ MR Conditional Defibrillation System in China	NCT03451721
405	Sapozhnikov et al. / Effect of Introducer Length on the Rate of Radial Artery Occlusion During Endovascular Coronary Procedures	NCT03854253
406	Abraham et al. / Evaluate Safety and Efficacy of the OPTIMIZER® System in Subjects With Moderate-to-Severe Heart Failure: FIX-HF-5C	NCT01381172
407	Abraham et al. / Evaluation of the Safety and Effectiveness of the OPTIMIZER System in Subjects With Heart Failure: FIX-HF-5	NCT00112125
408	Pang et al. / B-lines Lung Ultrasound Guided ED Management of Acute Heart Failure Pilot Trial	NCT03136198
409	Kini et al. / Side Branch FFR After Provisional Stenting	NCT03115580
410	Ali et al. / Shockwave Coronary Lithoplasty® Study (Disrupt CAD II)	NCT03328949
411	Cardiovascular Inflammation Reduction Trial - Inflammation Imaging Study	NCT02576067
412	Angermann et al. / CardioMEMS European Monitoring Study for Heart Failure	NCT02693691
413	Wray et al. / Contribution of Endothelin-1 to Exercise Intolerance in Heart Failure	NCT02124824

© 2025 Rivera FB et al. *JAMA Network Open*.

414	Berger et al. / Subclinical Cardiovascular Disease in Psoriatic Disease	NCT03228017
415	Kalantari et al. / Demonstration of Reverse Remodeling Effects of Entresto. Using Echocardiography Endocardial Surface Analysis	NCT02754518
416	Dukkipati et al. / Continued Access to PREVAIL (CAP2) - WATCHMAN Left Atrial Appendage (LAA) Closure Technology	NCT01760291
417	Ramos et al. / Arrhythmia Detection In Obstructive Sleep Apnea (ADIOS)	NCT02743520
418	DeAngelis et al. / Enoxaparin Versus Aspirin in Patients With Cancer and Stroke	NCT01763606
419	Taylor et al. / Response to Anti-hypertensives in Pregnant and Postpartum Patients	NCT03506724
420	McCabe et al. / Heart Failure Medication Adherence	NCT03402750
421	Repetitive Activation Patterns and Focal Impulses Identification and Ablation in Persistent AF	NCT03064451
422	O'Brien et al. / Nicotinamide Riboside in Systolic Heart Failure	NCT03423342
423	Gaudet et al. / Safety, Tolerability, and Effect of Alirocumab in High Cardiovascular Risk Patients With Severe Hypercholesterolemia Not Adequately Controlled With Conventional Lipid-modifying Therapies (ODYSSEY APPRISE)	NCT02476006
424	Daoud et al. / Same Day Subcutaneous ICD And Send Home (DASH) (DASH)	NCT03504839
425	Ridker et al. / Cardiovascular Inflammation Reduction Trial	NCT01594333
426	Gill et al. / Rate Control Therapy Evaluation in Permanent Atrial Fibrillation (RATE- AF)	NCT02391337
427	Lanfear et al. / A Study of Ivabradine in African-American/ Black Subjects With Heart Failure and Left Ventricular Systolic Dysfunction.	NCT03456856
428	Franchi et al. / A Pharmacodynamic Study Comparing Prasugrel Versus Ticagrelor in Patients Undergoing PCI With CYP2C19 Loss-of-function	NCT02065479
429	Gerdisch et al. / AtriClip® Left Atrial Appendage Exclusion Concomitant to Structural Heart Procedures (ATLAS)	NCT02701062
430	Persell et al. / The Smart Hypertension Control Study	NCT03288142
431	Stavrakis et al. / Transcutaneous Electrical Vagus Nerve Stimulation to Suppress Atrial Fibrillation	NCT02548754
432	Marcus et al. / The HOLIDAY (HOw ALcohol InDuces Atrial TachYarrhythmias) Study	NCT01996943
433	Vorapaxar in Patients With Prior Myocardial Infarction Treated With Prasugrel and Ticagrelor	NCT02545933
434	Giustino et al. / EXCEL Clinical Trial	NCT01205776
435	Evaluation of the iD-SystemTM, One-Handed Disposable Internal Defibrillation System.	NCT04011631
436	Hill et al. / Investigation of the Safety and Pharmacology of Dry Powder Inhalation of Treprostinil	NCT03399604
437	Simon et al. / A Dose-escalation Study in Subjects With Pulmonary Arterial Hypertension (PAH)	NCT03177603
438	Felker et al. / A Study of Continuous Infusions of HNO (Nitroxyl) Donor in Patients With Heart Failure and Impaired Systolic Function	NCT03357731
439	Chapin et al. / Apixaban Versus Warfarin for the Management of Post-operative Atrial Fibrillation	NCT02889562
440	Parikh et al. / The Drift-Reduction for Improved FFR Using Fiberoptic Technology (DRIFT) Study	NCT03848650
441	Celano et al. / Researching Emotions And Cardiac Health: Phase III	NCT03220204
442	Casida et al. / Self-Management App for Patients With Left-Ventricular Assist Devices	NCT03049748

443	Olgin et al. / Vest Prevention of Early Sudden Death Trial and VEST Registry	NCT01446965
444	The Effects of the Dietary Supplement CardioFlex Q10 on Reducing Cardiovascular Disease Risk Factors in Adults	NCT03826914
445	Spertus et al. / ISCHEMIA-Chronic Kidney Disease Trial	NCT01985360
446	Huebschmann et al. / Targeting Physical Activity to Improve Cardiovascular Health in Type 2 Diabetes	NCT02473926
447	Crowley et al. / The Effect of Farlong® NotoGinseng™ (Ginseng Plus®) on Cholesterol and Blood Pressure	NCT04069715
448	Labovitz et al. / Apixaban for Early Prevention of Recurrent Embolic Stroke and Hemorrhagic Transformation	NCT02283294
449	Vinnakota et al. / Study to Determine How Cialis Effects the Renal Function in Response to Volume Expansion in Preclinical Diastolic Cardiomyopathy (Aim3)	NCT02058095
450	Collins et al. / Texting for Mobility in Overweight/Obese Adults With Peripheral Artery Disease	NCT03694652
451	Mansour et al. / Safety and Effectiveness of STSF Catheter Evaluated for Treating Symptomatic Persistent Atrial Fibrillation (PsAF) (PRECEPT)	NCT02817776
452	Edoxaban Treatment Versus Vitamin K Antagonist in Patients With Atrial Fibrillation Undergoing Percutaneous Coronary Intervention	NCT02866175
453	Solomon et al. / Efficacy and Safety of LCZ696 Compared to Valsartan, on Morbidity and Mortality in Heart Failure Patients With Preserved Ejection Fraction	NCT01920711
454	Reddy et al. / RADAR Clinical Trial	NCT03263702
455	Clunies-Ross et al. / Carbetocin on Myocardial Repolarization Dynamics in Obstetrics Study	NCT03716076
456	Armstrong et al. / A Study of Vericiguat in Participants With Heart Failure With Reduced Ejection Fraction (HFrEF) (MK-1242-001)	NCT02861534
457	Poole et al. / ASSURE WCD Clinical Evaluation - Detection and Safety Study	NCT03887052
458	Buechel et al. / Deep-Learning Image Reconstruction in CCTA	NCT03980470
459	Felker et al. / Evaluate the Safety and Efficacy of 48-Hour Infusions of HNO (Nitroxyl) Donor in Hospitalized Patients With Heart Failure	NCT03016325
460	Angiolillio et al. / Low Maintenance Dose Ticagrelor Versus Clopidogrel in Diabetes Patients Undergoing PCI	NCT03437044
461	Azhar et al. / Nutritional Therapy Interventions in Heart Failure	NCT03424265
462	Kunneman et al. / SDM for Stroke Prevention in Atrial Fibrillation	NCT02905032
463	Nassif et al. / Dapagliflozin Effect on Symptoms and Biomarkers in Patients With Heart Failure	NCT02653482
464	Maron et al. / International Study of Comparative Health Effectiveness With Medical and Invasive Approaches (ISCHEMIA)	NCT01471522
465	Klein et al. / Imaging With a Radio Tracer to Guide VT Ablations	NCT01250912
466	Hearon et al. / Improving Metabolic Health in Patients With Diastolic Dysfunction	NCT03448185
467	Yuan et al. / The Effect of Venlafaxine on Language Function in Patients With Subcortical Aphasia: A fMRI Study	NCT03588572
468	Traaen et al. / Effect of Treatment of Sleep Apnea in Patients With Paroxysmal Atrial Fibrillation	NCT02727192
469	Mehran et al. / Ticagrelor With Aspirin or Alone in High-Risk Patients After Coronary Intervention	NCT02270242
470	Reddy et al. / Adjunctive Renal Denervation in the Treatment of Atrial Fibrillation	NCT01635998
471	Tardif et al. / Colchicine Cardiovascular Outcomes Trial (COLCOT)	NCT02551094
472	Kirtane et al. / EVOLVE Short DAPT Study	NCT02605447

© 2025 Rivera FB et al. *JAMA Network Open*.

473	McMurray et al. / Study to Evaluate the Effect of Dapagliflozin on the Incidence of Worsening Heart Failure or Cardiovascular Death in Patients With Chronic Heart Failure	NCT03036124
474	Pokorney et al. / Trial to Evaluate Anticoagulation Therapy in Hemodialysis Patients With Atrial Fibrillation	NCT02942407
475	Moroz et al. / Remote BP Monitoring in the PP Period	NCT03728790
476	Peer Support Dyads in Churches	NCT03463941
477	Ray et al. / Inclisiran for Subjects With ASCVD or ASCVD-Risk Equivalents and Elevated Low-density Lipoprotein Cholesterol	NCT03400800
478	Chou et al. / Walking Exercise on Memory, Subjective Cognitive Complaint, and Brain-derived Neurotrophic Factor for Hypertension	NCT04930263
479	Stewart et al. / eIMPACT Trial: Modernized Collaborative Care to Reduce the Excess CVD Risk of Older Depressed Patients	NCT02458690
480	CONVERGE - Epi/Endo Ablation For Treatment of Persistent Atrial Fibrillation(AF)	NCT01984346
481	Steinwender et al. / Micra Atrial TRacking Using A Ventricular AccELerometer 2	NCT03752151
482	Study to Assess the Safety, Pharmacokinetics, and Pharmacodynamics of DS-1040b in Subjects With Acute Ischemic Stroke	NCT02586233
483	Su et al. / STOP Persistent AF	NCT03012841
484	Schmidt-Ott et al. / FAST PV and mGFR™ Technology in Congestive Heart Failure	NCT03808948
485	A Study of the Effect of IW-1973 on the Exercise Capacity of Patients With Heart Failure With Preserved Ejection Fraction (HFpEF)	NCT03254485
486	Simonneau et al. / BAY63-2521 - Long-term Extension Study in Patients With Chronic Thromboembolic Pulmonary Hypertension	NCT00910429
487	Humbert et al. / BAY63-2521:Long-term Extension Study in Patients With Pulmonary Arterial Hypertension	NCT00863681
488	Paruchuri et al. / A Smartphone-based Application Post-myocardial Infarction to Manage Cardiovascular Disease Risk	NCT03416920
489	Ang et al. / Dapagliflozin and Measures of Cardiovascular Autonomic Function in Patients With Type 2 Diabetes (T2D)	NCT02973477
490	Mechanistic Evaluation of Glucose-lowering Strategies in Patients With Heart Failure	NCT02917031
491	Green et al. / Blood Pressure Checks for Diagnosing Hypertension (BP-CHECK)	NCT03130257
492	Chen et al. / China Micra Transcatheter Pacing Study	NCT03624504
493	Chin et al. / The Efficacy and Safety of Initial Triple Versus Initial Dual Oral Combination Therapy in Patients With Newly Diagnosed Pulmonary Arterial Hypertension	NCT02558231
494	DEF-314 Better Accuracy in Ejection Fraction (EF) Assessment With DEFINITY	NCT03571672
495	Shah et al. / Anti-inflammatory Effects of Colchicine in PCI	NCT01709981
496	Shah et al. / Colchicine in Percutaneous Coronary Intervention	NCT02594111
497	Maron et al. / Tadalafil for Pulmonary Hypertension Due to Chronic Lung Disease	NCT01862536
498	Caceres et al. / iPhone Helping Evaluate Atrial Fibrillation Rhythm Through Technology	NCT02731326
499	Hubers et al. / Study to Determine How Cialis Effects the Renal Function in Response to Volume Expansion in Preclinical Systolic Cardiomyopathy (Aim2)	NCT01970176
500	Ray et al. / Inclisiran for Participants With Atherosclerotic Cardiovascular Disease and Elevated Low-density Lipoprotein Cholesterol	NCT03399370

© 2025 Rivera FB et al. *JAMA Network Open*.

501	Vonk Noordegraaf et al. / REPAIR: Right vEntricular Remodeling in Pulmonary ArterIal hypeRtension	NCT02310672
502	Study of Ambrisentan in Participants With Pulmonary Hypertension	NCT00777920
503	Kuliopulos et al. / Safety of PZ-128 in Subjects Undergoing Non-Emergent Percutaneous Coronary Intervention	NCT02561000
504	Damman et al. / Effects of Empagliflozin on Clinical Outcomes in Patients With Acute Decompensated Heart Failure	NCT03200860
505	Toh et al. / Cardio-vascular Protective Effects of Wolfberry in Middle-aged and Older Adults	NCT03535844
506	Catanzaro et al. / Microfidelity (MIFI) Ablation Technology Versus Standard Ablation Catheter for Atrioventricular Nodal Ablation	NCT03114124
507	Myers et al. / PCI Alternative Using Sustained Exercise	NCT03520400
508	Shah et al. / Exercise Therapy to Reduce Heart Failure Symptoms; Sorting Mechanisms of Benefit	NCT03648762
509	Anker et al. / This Study Tests Empagliflozin in Patients With Chronic Heart Failure With Reduced Ejection Fraction (HFrEF). The Study Looks at How Far Patients Can Walk in 6 Minutes and at Their Heart Failure Symptoms	NCT03448419
510	Taqueti et al. / Coronary Flow Reserve to Assess Cardiovascular Inflammation (CIRT-CFR)	NCT02786134
511	Hypertension In Postpartum Preeclampsia Study	NCT03011567
512	Anker et al. / This Study Tests Empagliflozin in Patients With Chronic Heart Failure With Preserved Ejection Fraction (HFpEF). The Study Looks at How Far Patients Can Walk in 6 Minutes and at Their Heart Failure Symptoms.	NCT03448406
513	Armstrong et al. / Patient-reported Outcomes in Vericiguat-treated Patients With HFpEF	NCT03547583
514	Janevic et al. / A Partnership to Translate an Evidence-based Intervention (Take Heart) for Vulnerable Older Adults With Heart Disease	NCT02950818
515	Holter Versus Zio Patch Electrocardiographic Monitoring in Children	NCT03309956
516	Valgimigli et al. / XIENCE 28 Global Study	NCT03355742
517	Rengo et al. / Transitional Rehabilitation in CABG Patients	NCT03892460
518	Voors et al. / v4 Study Evaluating the Safety, Tolerability and Preliminary Pharmacokinetics and Pharmacodynamics of MYK-491	NCT03447990
519	Pieske et al. / A Randomized, Double-blind Controlled Study Comparing LCZ696 to Medical Therapy for Comorbidities in HFpEF Patients	NCT03066804
520	Barsuk et al. / Use of Simulation to Improve VAD Self-management	NCT03073005
521	Juraschek et al. / The Effect of Sodium Reduction on Blood Pressure and Physical Function in Older Adults	NCT04074941
522	Ballantyne et al. / Assessment of the Long-Term Safety and Efficacy of Bempedoic Acid (CLEAR Harmony OLE)	NCT03067441
523	Morgan et al. / A Study to Assess the Safety, Tolerability and Efficacy of IONIS- AGT-LRx, an Antisense Inhibitor Administered Subcutaneously to Hypertensive Participants With Controlled Blood Pressure	NCT03714776
524	Franchi et al. / Impact of Chronic Kidney Disease on the Effects of Ticagrelor in Patients With Diabetes and Coronary Artery Disease	NCT02539160
525	Kautzner et al. / DiamondTemp™ Ablation System for the Treatment of Paroxysmal Atrial Fibrillation	NCT03334630
526	Hill et al. / Safety and Efficacy of Nerinetide (NA-1) in Subjects Undergoing Endovascular Thrombectomy for Stroke	NCT02930018
527	Tardif et al. / Study of ISIS 678354 (AKCEA-APOCIII-LRx) in Participants With Hypertriglyceridemia and Established Cardiovascular Disease (CVD)	NCT03385239
528	Khera et al. / Pecto-Intercostal Fascial Block for Postoperative Analgesia After Cardiac Surgery	NCT03482973

© 2025 Rivera FB et al. *JAMA Network Open*.

529	Halle et al. / Exercise Capacity Study of LCZ696 vs. Enalapril in Patients With Chronic Heart Failure and Reduced Ejection Fraction.	NCT02768298
530	Still et al. / A COmmunity and Tech-Based ApproaCh for Hypertension Self-MANagement	NCT03724487
531	McCullough et al. / Extended Release Exenatide Versus Placebo In Diabetic Patients With Type 4 Cardiorenal Syndrome	NCT02251431
532	Batuman et al. / Effect of Renin-angiotensin-system Blockade on Urinary Free Light Chains in Patients With Type 2 Diabetes Mellitus	NCT02046395
533	Djousse et al. / Intervention With Vitamin D and Omega-3 Supplements and Incident Heart Failure	NCT02271230
534	Lopes Perdigao et al. / Furosemide for Accelerated Recovery of Blood Pressure Postpartum	NCT03556761
535	Gold et al. / Understanding Outcomes With the EMBLEM™ S-ICD in Primary Prevention Patients With Low Ejection Fraction	NCT02433379
536	Henry et al. / Safety and Potential Bioactivity of CLBS16 in Patients With Coronary Microvascular Dysfunction and Without Obstructive Coronary Artery Disease	NCT03508609
537	Felker et al. / An Investigational Study of Continuous 8-Hour Intravenous Administrations of BMS-986231 in Participants With Heart Failure and Reduced Heart Function Given a Standard Dose of Loop Diuretic	NCT03730961
538	Subramaniam et al. / Comparison of Sternal Wound Infiltration With Liposomal Bupivacaine v. Bupivacaine Hydrochloride	NCT03270514
539	Johnston et al. / THALES - Acute STroke or Transient IscHaemic Attack Treated With TicAgreLor and ASA for PrEvention of Stroke and Death	NCT03354429
540	Bhardwaj et al. / Myocardial Infarction, COmbined-device, Recovery Enhancement Study	NCT03760796
541	Relative Bioavailability Study of Marketed and Lower Dose Ambrisentan in Healthy Adult Participants	NCT04095286
542	Gardiner et al. / Reducing Disparities in the Treatment of Hypertension Using the OWL mHealth Tool	NCT03974334
543	Sapp et al. / Peanuts and Glycemic Control	NCT03654651
544	Waxman et al. / Safety and Efficacy of Inhaled Treprostinil in Adult PH With ILD Including CPFE	NCT02630316
545	Kandzari et al. / Scoreflex NC - Scoring PTCA Catheter	NCT03763747
546	Cannon et al. / Cardiovascular Outcomes Following Ertugliflozin Treatment in Type<br>2 Diabetes Mellitus Participants With Vascular Disease, The VERTIS CV Study (MK-8835-004)	NCT01986881
547	Okumura et al. / Study of DU-176b Aged 80 Years or Older	NCT02801669
548	Dogan et al. / Disease Management for Coronary Artery Patients	NCT04556006
549	Lilliu et al. / Effects of Echo-optimization of Left Ventricular Assist Devices on Functional Capacity (VAFRACT)	NCT03937570
550	Hung et al. / Metformin in Kidney Disease	NCT02252081
551	Saman et al. / Pre-Diabetes Cardiovascular (CV) Care (Pre-Diabetes Wizard)	NCT02759055
552	Rodgers et al. / Weight Loss With Exenatide Treatment	NCT01590433
553	Bush et al. / Study of the Gut Hormone Analogue G3215 in Adult Subjects	NCT02692040
554	Petersen et al. / MOWI Research Pilot	NCT03104192
555	Patel et al. / Social Incentives To Encourage Physical Activity and Understand Predictors (STEPUP)	NCT03311230
556	Safety and Tolerability Study of MEDI0382 in Japanese Preobese or Obese Subjects With Type 2 Diabetes	NCT03645421

© 2025 Rivera FB et al. *JAMA Network Open*.

557	Namynanik et al. / Comparing the Efficacy of a Dual-Frequency LLLT Device With a Sham Device as a Therapy for Adipose Tissue Loss	NCT03811093
558	Dapagliflozin and Metformin, Alone and in Combination, in Overweight/Obese Prior GDM Women	NCT02338193
559	A Study to Evaluate the Safety and Efficacy of JNJ-64565111 in Non-diabetic Severely Obese Participants	NCT03486392
560	Heymsfield et al. / Safety, Pharmacokinetics and Efficacy of Bimagrumab in Overweight and Obese Patients With Type 2 Diabetes	NCT03005288
561	Basolo et al. / Investigation of the Gut Microbiota in Regulating Nutrient Absorption in Humans	NCT02037295
562	Sherwood et al. / BestFIT: a Personalized Weight Loss Program	NCT02368002
563	A Study to Evaluate the Safety and Efficacy of JNJ-64565111 in Severely Obese Participants With Type 2 Diabetes Mellitus	NCT03586830
564	Pearl et al. / Psychological Intervention to Reduce Weight Bias Internalization	NCT03572218
565	Elkind-Hirsch et al. / Combined Liraglutide and Metformin Therapy in Women With Previous Gestational Diabetes Mellitus (GDM)	NCT01234649
566	Heise et al. / A Study to Evaluate the Safety and Tolerability of MEDI0382 in Overweight and Obese Participants With Type 2 Diabetes Mellitus	NCT03745937
567	Butryn et al. / FitLink: Improving Weight Loss Maintenance by Using Digital Data to Provide Support and Accountability	NCT03337139
568	Ruetzler et al. / McGrath Videolaryngoscopy and Direct Laryngoscopy in Morbidly Obese Patients	NCT03467048
569	Brookfield et al. / Magnesium Sulfate in Obese Preeclamptics	NCT02835339
570	Kittah et al. / Exendin-9,39 and Satiety After Bariatric Surgery	NCT02779075
571	Unick et al. / Feelings About Exercise	NCT03598647
572	Moore et al. / Reporting Patient Generated Health Data and Patient Reported Outcomes With Health Information Technology	NCT03386773
573	Snitker et al. / A Study Comparing the Injection Site Pain Experience After the Injection of 2 Different Solutions of Semaglutide With 2 Different Injection Pens, a Compound for the Treatment of Type 2 Diabetes and Obesity	NCT04007107
574	Barnouin et al. / Lifestyle Intervention and Testosterone Replacement in Obese Seniors (LITROS)	NCT02367105
575	Legget et al. / Neuronal and Behavioral Effects of Implicit Priming in Obese Individuals	NCT02347527
576	Butryn et al. / Project Impact: An Innovative Approach to Weight Loss Maintenance	NCT02363010
577	West et al. / Internet Assisted Obesity Treatment Enhanced With Financial Incentives (iREACH3)	NCT02688621
578	Frost et al. / The Acute Effect of Propionate on Energy Homeostasis (PROEM)	NCT04093453
579	Horowitz et al. / Protection Against Insulin Resistance in Obesity (PAIR)	NCT02717832
580	Barnett et al. / Deaf Weight Wise 2.0: A Healthy Lifestyle Intervention With Deaf Adults Who Are Overweight or Obese (DWW2)	NCT03060525
581	Vitolins et al. / Healthy Living Partnerships to Prevent Diabetes in Veterans Pilot Study (HELP Vets)	NCT02835495
582	Katzmarzyk et al. / Promoting Successful Weight Loss in Primary Care in Louisiana (PROPEL)	NCT02561221
583	Burger et al. / Examination of Bromocriptine on Homeostatic and Hedonic Mechanisms of Food Intake in Individuals at High Risk for T2DM	NCT05405244
584	Harvey et al. / Examining Cooking as a Health Behavior	NCT03783962
585	Kocsis et al. / Depression, Obesity and Inflammatory Markers	NCT02765100
586	McElfish et al. / Healthy Body Healthy Souls in the Marshallese Population	NCT03377244

© 2025 Rivera FB et al. *JAMA Network Open*.

587	Harrison et al. / Effect of LIK066 on Reduction of Fatty Content in Livers of Obese Patients	NCT03205150
588	Pagoto et al. / RELAX: A Mobile Application Suite Targeting Obesity and Stress	NCT02615171
589	Swindle et al. / Developing and Testing Implementation Strategies for Evidence- Based Obesity Prevention in Childcare	NCT03075085
590	Multiple Ascending Dose Study of AMG 598 in Adults With Obesity	NCT03757130
591	A Study to Evaluate the Effect of MEDI0382 on Energy Balance in Overweight and Obese Participants With Type 2 Diabetes Mellitus	NCT03596177
592	Befort et al. / Rural Engagement in Primary Care for Optimizing Weight Reduction	NCT02456636
593	MyoKardia, Inc. / A Phase 2 Study of Mavacamten in Adults With Symptomatic Non-Obstructive Hypertrophic Cardiomyopathy (nHCM)	NCT03442764
594	Syneos Health / DEF-315 Better Accuracy in Ejection Fraction (EF) Assessment With DEFINITY	NCT03719612
595	Karmpaliotis et al. / A Clinical Trial to Assess the SYNERGY 48 mm Stent System for the Treatment of Atherosclerotic Lesion(s)	NCT03350542
596	Sankyo et al. / Safety of DS-1040b in Acute Ischemic Stroke Patients Treated With Thrombectomy	NCT03198715
597	Biotronik, Inc. / QP ExCELs: MultiPole Pacing (MPP) Sub-Study	NCT03155724
598	Thomas et al. / Educational Videos to Address Racial Disparities in Implantable Cardioverter Defibrillator Therapy	NCT02819973
599	Bayer / Riociguat rEplacing PDE-5i Therapy evaLuated Against Continued PDE-5i thErapy	NCT02891850
600	Pfister et al. / Effect of Selexipag on Daily Life Physical Activity of Patients With Pulmonary Arterial Hypertension.	NCT03078907
601	United Therapeutics / An Open-Label Extension Trial of UT-15C Sustained-release (SR) in Subjects With Pulmonary Arterial Hypertension	NCT01027949
602	Badimon et al. / Are the "Cardiac Benefits" of Empagliflozin Independent of Its Hypoglycemic Activity? (ATRU-4).	NCT03485222
603	Tarajki et al. / A Fib Clinic of the Future Using KardiaPro Platform for Chronic Care of Patients With AF After Ablation Procedure	NCT03557034
604	Regeneron Pharmaceuticals / Evaluating Effect of the Study Drug Praluent (Alirocumab) on Neurocognitive Function When Compared to Placebo	NCT02957682
605	AstraZeneca / DETERMINE-reduced - Dapagliflozin Effect on Exercise Capacity Using a 6-minute Walk Test in Patients With Heart Failure With Reduced Ejection Fraction	NCT03877237
606	Kandzari et al. / A Single Arm Trial With Resolute Onyx in ONE-Month DAPT for High-Bleeding Risk Patients Who Are Considered One-Month Clear (Onyx ONE Clear)	NCT03647475
607	Kosiborod et al. / Empagliflozin Impact on Hemodynamics in Patients With Heart Failure	NCT03030222
608	Guo et al. / Evaluate the Performance and Safety of Comet Pressure Guidewire in the Measurement of FFR	NCT04098172
609	Rocco et al. / Clinical Study to Assess the Efficacy and Safety of Macitentan in Patients With Pulmonary Hypertension After Left Ventricular Assist Device Implantation	NCT02554903
610	MyoKardia, Inc. / Clinical Study to Evaluate Mavacamten (MYK-461) in Adults With Symptomatic Obstructive Hypertrophic Cardiomyopathy	NCT03470545
611	Attune Medical / Esophageal Cooling for AF Ablation	NCT03691571
612	Feld et al. / Evaluation of the CIRCA Monitoring System in Prevention of Esophageal Lesions Following RFCA	NCT02467166
613	Raj et al. / Hemodynamic Effects of Compression in POTS	NCT03484273

© 2025 Rivera FB et al. *JAMA Network Open*.

614	Hospital Civil de Guadalajara / The Effect in Renal Function on Patients With Type 1 Cardiorenal Syndrome Treated With Two Strategies of Furosemide.	NCT04393493
615	Hage et al. / Heart Rate Response to Regadenoson and Sudden Cardiac Death	NCT01842035
616	Marcus et al. / Individualized Studies of Triggers of Paroxysmal Atrial Fibrillation	NCT03323099
617	Tenax Therapeutics, Inc. / Hemodynamic Evaluation of Levosimendan in Patients With PH-HFpEF	NCT03541603
618	AstraZeneca / AZD5718 Phase IIa Study to Evaluate Efficacy, Safety and Tolerability of Oral AZD5718 in Patients With Coronary Artery Disease (CAD).	NCT03317002
619	Saver et al. / Treatment With Intent to Generate Endovascular Reperfusion	NCT03474549
620	Brittain et al. / A Mobile Health Intervention in Pulmonary Arterial Hypertension	NCT03069716
621	Townsend et al. / SPYRAL PIVOTAL - SPYRAL HTN-OFF MED Study	NCT02439749
622	Duke University / Exercise and Pharmacotherapy for Anxiety in Cardiac Patients	NCT02516332
623	Brown et al. / Effect of Chronic ACE and DPP4 Inhibition on Blood Pressure	NCT02130687
624	Boehringer Ingelheim / EMPagliflozin outcomE tRial in Patients With chrOnic heaRt Failure With Reduced Ejection Fraction (EMPEROR-Reduced)	NCT03057977
625	Lakkireddy et al. / DOACs for Stroke Prevention Post Ventricular Tachycardia Ablation	NCT02666742
626	Erlinge et al. / PROSPECT II & PROSPECT ABSORB - an Integrated Natural History Study and Randomized Trial.	NCT02171065
627	Reyentovich et al. / HCV Positive Heart Donors	NCT03382847
628	Stone et al. / Disrupt CAD IV With the Shockwave Coronary IVL System	NCT04151628
629	Taub et al. / Effect of Ivabradine on Patients With Postural Orthostatic Tachycardia Syndrome	NCT03182725
630	Budoff et al. / Effect of Vascepa on Improving Coronary Atherosclerosis in People With High Triglycerides Taking Statin Therapy	NCT02926027
631	Eli Lilly and Company / A Study That Looks at the Function of the Heart in Patients With Heart Failure Who Take Empagliflozin	NCT03332212
632	Nissen et al. / Outcomes Study to Assess STatin Residual Risk Reduction With EpaNova in HiGh CV Risk PatienTs With Hypertriglyceridemia	NCT02104817
633	Vardeny et al. / INfluenza Vaccine to Effectively Stop Cardio Thoracic Events and Decompensated Heart Failure	NCT02787044
634	Gray et al. / Rapid Assessment of Potential Ischaemic Heart Disease With CTCA	NCT02284191
635	Kitzman et al. / A Trial of Rehabilitation Therapy in Older Acute Heart Failure Patients	NCT02196038
636	Massachusetts General Hospital / Esophageal Deviation in Atrial Fibrillation Ablation	NCT03261973
637	Wu et al. / Vitamin C Supplementation Intervention	NCT04036110
638	Bolao et al. / Electrical Coupling Information From The Rhythmia HDx System And DirectSense Technology In Subjects With Paroxysmal Atrial Fibrillation	NCT03232645
639	Simari et al. / Combination of Mesenchymal and C-kit+ Cardiac Stem Cells as Regenerative Therapy for Heart Failure	NCT02501811
640	Wazni et al. / STOP AF First: Cryoballoon Catheter Ablation in an Antiarrhythmic Drug Naive Paroxysmal Atrial Fibrillation	NCT03118518
641	Kapoor et al. / Shared Decision-Making: AFib 2gether Mobile App	NCT04118270
642	Jones et al. / Aspirin Dosing: A Patient-Centric Trial Assessing Benefits and Long- term	NCT02697916

© 2025 Rivera FB et al. *JAMA Network Open*.

643	National Institute of Diabetes and Digestive and Kidney Diseases (NIDDK) / Coordinating Pragmatic Primary Care Population Management for Obesity	NCT03998046
644	Birk et al. / Investigating Fear Of Recurrence as a Modifiable Mechanism of Behavior Change	NCT03853213
645	Matsumaru et al. / Safety Trial of OPC-61815 Injection in Patients With Congestive Heart Failure Who Have Difficulty With or Are Incapable of Oral Intake	NCT03962101
646	Gottlieb et al. / Oxygen Versus PAP for Sleep Apnea in Heart Failure	NCT01807897
647	Gamboa et al. / Peripheral Dopamine in Postural Tachycardia Syndrome	NCT00685919
648	AstraZeneca / DETERMINE-preserved - Dapagliflozin Effect on Exercise Capacity Using a 6-minute Walk Test in Patients With Heart Failure With Preserved Ejection Fraction	NCT03877224
649	Ionis Pharmaceuticals, Inc. / A Study to Assess the Safety, Tolerability and Efficacy of IONIS-AGT-LRx	NCT04083222
650	Sato et al. / Efficacy and Safety Trial of OPC-61815 Injection Compared With Tolvaptan 15-mg Tablet in Patients With Congestive Heart Failure	NCT03772041
651	Hernandez et al. / Multiple Cardiac Sensors for the Management of Heart Failure	NCT03237858
652	Ponikowski et al. / Study to Compare Ferric Carboxymaltose With Placebo in Patients With Acute Heart Failure and Iron Deficiency	NCT02937454
653	McMurray et al. / The Effects of Sacubitril/Valsartan Compared to Valsartan on LV Remodelling in Asymptomatic LV Systolic Dysfunction After MI	NCT03552575
654	Jankelson et al. / Introductory Clinical Trial for Measuring Patients Before, During, and After an Electrophysiology (EP) Procedure With a Novel, Body-Worn Sensor	NCT03657134
655	Shea et al. / Retail Outlet Health Kiosk Hypertension Trial	NCT03515681
656	Alexander et al. / Evaluate the Efficiency Impact of Conducting Active Temperature Management During Cardiac Cryoablation Procedures	NCT04087122
657	Chung et al. / Effects of Family Sodium Watcher Program on Outcomes in Heart Failure Patient-Family Caregiver Dyads	NCT03560206
658	Tung et al. / His Bundle Pacing Versus Coronary Sinus Pacing for Cardiac Resynchronization Therapy	NCT02700425
659	Redeker et al. / Insomnia Self-Management in Heart Failure	NCT02660385
660	Weiss et al. / Inflammation and Coronary Endothelial Function	NCT02366091
661	Weiss et al. / Inflammatory Pathogenesis of Coronary Atherosclerosis in HIV	NCT02624180
662	Bernstein et al./ Rate of Atrial Fibrillation Through 12 Months in Patients With Recent Ischemic Stroke of Presumed Known Origin	NCT02700945
663	Worldwide Clinical Trials / Phase 2b Study of KBP-5074 in Subjects With Uncontrolled Hypertension and Advanced Chronic Kidney Disease	NCT03574363
664	Sankyo et al. / Prasugrel Switching Study in Patients With Acute Coronary Syndrome (ACS) Who Underwent Percutaneous Coronary Intervention (PCI)	NCT03672097
665	Steinberg et al. / Junctional AV Ablation in CRT-D: JAVA-CRT	NCT02946853
666	Parodi et al. / Ticagrelor Administered as Standard Tablet or Orodispersible Formulation	NCT03822377
667	Steinhubl et al. / mHealth Screening to Prevent Strokes	NCT02506244
668	Amgen et al. / Registrational Study With Omecamtiv Mecarbil (AMG 423) to Treat Chronic Heart Failure With Reduced Ejection Fraction	NCT02929329
669	Gilbert et al. / A SAD/MAD Study of Safety, Tolerability and Pharmacologic Activity of BT200 in Normal Volunteers	NCT04103034

© 2025 Rivera FB et al. *JAMA Network Open*.

670	Anstrom et al. / EntrestoTM (LCZ696) In Advanced Heart Failure (LIFE Study)	NCT02816736
671	Gold et al. / Strategic Management to Optimize Response To Cardiac Resynchronization Therapy	NCT03089281
672	Dave et al. / Noninvasive Real-time Intracardiac Pressure Measurements Using Subharmonic Ultrasound	NCT03245255
673	Joshi et al. / Impact of Liraglutide 3.0 on Body Fat Distribution	NCT03038620
674	Svedjeholm et al. / Glutamate for Metabolic Intervention in Coronary Surgery II	NCT02592824
675	Francis et al. / Self-Assessment Method for Statin Side-effects Or Nocebo	NCT02668016
676	Pereira et al. / Tailored Antiplatelet Therapy Following PCI	NCT01742117
677	Angiolillo et al. / Impact of Evolocumab on the Effects of Clopidogrel in Patients With High On-Treatment Platelet Reactivity	NCT03096288
678	Medtronic Vascular / RESOLUTE ONYX Post-Approval Study (Bifurcation Cohort)	NCT03584464
679	Radhakrishnan et al. / Sensor-controlled Digital Game for Heart Failure Self-management	NCT03947983
680	Allen et al. / Electronic Health Record-leveraged, Patient-centered, Intensification of Chronic Care for HF	NCT03334188
681	Samir et al. / Strategic Management to Improve CRT Using Multi-Site Pacing Post Approval Study (Reference # C1918)	NCT03257436
682	Ray et al. / Effect of RVX000222 on Time to Major Adverse Cardiovascular Events in High-Risk T2DM Subjects With CAD	NCT02586155
683	Samady et al. / Wall Shear Stress and Neointimal Healing Following PCI in Angulated Coronary Vessels	NCT02098876
684	Brett et al. / MOMENTUM 3 Continued Access Protocol	NCT02892955
685	Gamboa et al. / Dietary Sodium's Effect on Urinary Sodium and Dopamine Excretion in Patients With Postural Tachycardia Syndrome	NCT01563107
686	Gamboa et al. / Dietary Salt in Postural Tachycardia Syndrome	NCT01547117
687	Actelion / Clinical Study to Assess the Long-term Safety and Tolerability of ACT 064992 in Patients With Symptomatic Pulmonary Arterial Hypertension	NCT00667823
688	Merck Sharp & Dohme LLC / A Study of Single Doses of MK -5475 on Pulmonary Vascular Resistance (MK-5475-002)	NCT03744637
689	Nambi et al. / Biomarker Guided Therapies in Stage A/B Heart Failure	NCT02230891
690	University of Pretoria / A Trial to Compare American Heart Association (AHA) and Simple (SIM)Method to Give Adenosine to Treat Supra-ventricular Tachycardia (SVT)	NCT04392362
691	Monlezun et al. / Cooking for Health Optimization With Patients	NCT03443635
692	Amgen / Imaging of Coronary Plaques in Participants Treated With Evolocumab	NCT03570697
693	Neuzil et al. / A Safety and Feasibility Study of the FARAPULSE Endocardial Ablation System to Treat Paroxysmal Atrial Fibrillation	NCT03714178
694	Abbott Medical Devices / Safety and Effectiveness of TactiCath™ Contact Force, Sensor Enabled™ (TactiCath SE) Catheter for Ablation of Drug Refractory, Symptomatic, Persistent Atrial Fibrillation	NCT03650556
695	Taher et al. / Cryoablation System FIM/CE Mark Study	NCT03723070
696	Lepor et al. / A Dose-range Finding Study of MAA868 in Patients With Atrial Fibrillation	NCT04213807
697	Bayer / REALIsM-HF Pilot Study	NCT03507439
698	Vassy et al. / Integrating Pharmacogenetics In Clinical Care	NCT02871934
699	Kronenberg et al. / Myocardial Perfusion, Oxidative Metabolism, and Fibrosis in HFpEF	NCT02589977

© 2025 Rivera FB et al. *JAMA Network Open*.

700	Doshi et al. / Investigational Device Evaluation of the WATCHMAN FLX™ LAA Closure Technology	NCT02702271
701	Luberto et al. / MBCT Delivered Via Group Videoconferencing for ACS Patients With Elevated Depression Symptoms	NCT03878160
702	Angiolillo et al. Pharmacodynamic Effects of Low-dose Rivaroxaban With Antiplatelet Therapies	NCT03718429
703	Iqvia Pty Ltd / ECG App Algorithms Clinical Validation Study	NCT04247581
704	Kim et al. / A Post-market Clinical Evaluation of St. Jude Medical™ MR Conditional ICD System on Patients Undergoing Magnetic Resonance Imaging	NCT02877693
705	Dukkipati et al. / Bipolar Ventricular Tachycardia (VT) Study	NCT02374476
706	Bravata et al. / Protocol-guided Rapid Evaluation of Veterans Experiencing New Transient Neurological Symptoms	NCT02769338
707	Mehran et al. / XIENCE 28 USA Study	NCT03815175
708	Grace et al. / AcQMap Objectively Visualize the Etiology of Recurrent AF Following a Failed AF Ablation	NCT03368781
709	Banning et al. / Oxford Acute Myocardial Infarction - Pressure-controlled Intermittent Coronary Sinus Occlusion	NCT03473015
710	Masimo Corporation / Validation of Noninvasive Blood Pressure Device	NCT04676152
711	Raj et al. / Effect of Dietary Sodium Intake on Vascular Endothelium	NCT01550315
712	New Ulm at HOME (Healthy Offerings Via the Mealtime Environment), NU-HOME	NCT02973815
713	Family Empowerment for Enhanced Development	NCT03641716
714	A Study Comparing the Injection Site Pain Experience After the Injection of Semaglutide B and Semaglutide D With 2 Different Injection Pens, a Compound for the Treatment of Type 2 Diabetes and Obesity	NCT04143945
715	Bicalutamide With or Without Metformin for Biochemical Recurrence in Overweight or Obese Prostate Cancer Patients	NCT02614859
716	Comparison of Injection Site Pain Experience for Semaglutide and Dulaglutide sc	NCT04189848
717	Teaching Obesity Treatment Options to Adult Learners Trial	NCT03856320
718	Research Study Investigating How Well Semaglutide Works in People Suffering From Overweight or Obesity	NCT03548987
719	A Mobile Intervention to Reduce Pain and Improve Health (MORPH)	NCT03377634
720	Research Study Investigating How Well NNC0174-0833 Works in People Suffering From Overweight or Obesity.	NCT03856047
721	Peer Navigators to Address Obesity-Related Concerns for African Americans With Serious Mental Illness	NCT03382782
722	Exercise After Clinically Significant Weight Loss	NCT03685123
723	Research Study to Look at How Well Semaglutide is at Lowering Weight When Taken Together With an Intensive Lifestyle Program	NCT03611582
724	Research Study Investigating How Well Semaglutide Works in People With Type 2 Diabetes Suffering From Overweight or Obesity	NCT03552757
725	Weight Management in Rural Communities	NCT02932748
726	Targeting Obesity to Optimize Health in Cardiac Rehab (TOPCARE)	NCT03423238
727	Pilot Study of Time Restricted Feeding as a Weight Loss Intervention	NCT03571048
728	The GEM (Goals for Eating and Moving) Study	NCT03006328
729	Real World Effectiveness of Combining an Employer-based Weight Management Program With Medication for Chronic Weight Management in Employees With Obesity	NCT03799198

© 2025 Rivera FB et al. *JAMA Network Open*.

730	The HOMBRE Trial: Comparing Two Innovative Approaches to Reduce Chronic Disease Risk Among Latino Men	NCT03092960
731	Pilot of Lifestyle and Asthma Intervention	NCT03291808
732	PDE5 Inhibition for Obesity-Related Cardiometabolic Dysfunction	NCT02819440
733	Negative Pressure Wound Therapy in Obese Gynecologic Oncology Patients	NCT02309944
734	Social and Financial Incentives to Increase Physical Activity Among Overweight and Obese Veterans	NCT03563027
735	Approaches for Improving Long-term Weight Loss	NCT03799289
736	tDCS for Impulsivity and Compulsivity in Obesity	NCT04405089
737	Coordinating Pragmatic Primary Care Population Management for Obesity	NCT03998046
738	Self-monitoring Activity: a Randomized Trial of Game-oriented Applications	NCT02341235
739	MOWI Home-Based Pilot	NCT03104205
740	Lifestyle Intervention in Overweight and Obese Pregnant Hispanic Women	NCT01868230
741	Continuous Glucose Monitoring in Diabetes and Prediabetes	NCT03805412
742	Positive Psychology for Physical Activity Promotion	NCT03826173
743	EQW, DAPA, EQW/DAPA, DAPA/MET ER and PHEN/TPM ER in Obese Women With PolycysticOvary Syndrome (PCOS)	NCT02635386
744	Studying the Impact of Product Packaging in a Virtual Store Environment	NCT04381481
745	Compare the Oncological Benefit of Deep Neuromuscular Block in Gastric Cancer Obesity Patient	NCT03196791
746	REAL HEALTH-Diabetes: Reach Ahead for Lifestyle and Health-Diabetes	NCT02320253
747	A Randomized, Controlled Pilot Study of a Patient-Initiated Approach to Increasing Weight Communication in Primary Care	NCT04486235
748	Empowered With Movement to Prevent Obesity and Weight Regain	NCT02923674
749	Daily Caloric Restriction and Intermittent Fasting in Overweight and Obese Adults With Autosomal Dominant Polycystic Kidney Disease	NCT03342742
750	Multicenter Endoscopic Sleeve Gastrectomy (ESG) Trial (MERIT Trial)	NCT03406975
751	INtervention Study In overweiGHT Patients With COPD	NCT02634268
752	Effect of Liraglutide for Weight Management in Paediatric Subjects With Prader- Willi Syndrome	NCT02527200
753	DPE Technique in Labor Epidural for Morbidly Obese Women	NCT03074695
754	Setmelanotide (RM-493), Melanocortin-4 Receptor (MC4R) Agonist, in Bardet- Biedl Syndrome (BBS) and Alström Syndrome (AS) Participants With Moderate to Severe Obesity	NCT03746522
755	Skeletal Muscle Diacylglycerol and Sphingolipids - Impact of Localization and Species on Insulin Resistance in Humans	NCT03077360
756	STEP 6: Research Study Investigating How Well Semaglutide Works in People Living With Overweight or Obesity	NCT03811574
757	Cooking for Health Optimization With Patients	NCT03443635
758	TOSS Feasibility + Fitbit Community = Reduced Obesity in Older Black Women	NCT04114071
759	Mi Puente: My Bridge to Better Cardiometabolic Health and Well-Being	NCT02723019
760	Bicki et al. / Activity Trackers for Improving BP	NCT03325426
761	Lloyd et al. / Comparison of Percutaneous Closure to Manual Compression for Hemostasis	NCT04180540
762	Bonaca et al. / A Study to Evaluate the Safety and Efficacy of MEDI6012 in Acute ST Elevation Myocardial Infarction (REAL-TIMI 63B)	NCT03578809

© 2025 Rivera FB et al. *JAMA Network Open*.

763	Del Prato et al. / A Study of Tirzepatide (LY3298176) Once a Week Versus Insulin Glargine Once a Day in Participants With Type 2 Diabetes and Increased Cardiovascular Risk (SURPASS-4)	NCT03730662
764	Mazur et al. / Evaluating Active Esophageal Cooling During Cardiac Ablation Procedures	NCT04063761
765	Pitt et al. / Efficacy and Safety of Finerenone in Subjects With Type 2 Diabetes Mellitus and the Clinical Diagnosis of Diabetic Kidney Disease (FIGARO-DKD)	NCT02545049
766	Pfeffer et al. / Prospective ARNI vs ACE Inhibitor Trial to DetermIne Superiority in Reducing Heart Failure Events After MI (PARADISE-MI)	NCT02924727
767	Cummings et al. / Southeastern Collaboration to Improve Blood Pressure Control	NCT02866669
768	Van Mieghem et al. / Edoxaban Compared to Standard Care After Heart Valve Replacement Using a Catheter in Patients With Atrial Fibrillation (ENVISAGE-TAVI AF)	NCT02943785
769	A Study to Evaluate Whether Macitentan is an Effective and Safe Treatment for Patients With Heart Failure With Preserved Ejection Fraction and Pulmonary Vascular Disease (SERENADE)	NCT03153111
770	Pierce et al. / CoQ10 and D-ribose in Patients With Diastolic Heart Failure	NCT03133793
771	Tung et al. / Pan-Asia United States PrEvention of Sudden Cardiac Death Catheter Ablation Trial (PAUSE-SCD)	NCT02848781
772	Stavrakis et al. / Neuromodulation to Treat Patients With Heart Failure With Preserved Ejection Fraction	NCT03327649
773	Ellis et al. / Perioperative Antibiotic Therapy to Prevent Cardiac Implantable Electronic Device Infections. (ENVELOPE)	NCT02809131
774	Min et al. / The Effect of GLP-1 Agonists Versus OCs on Reproductive Disorders and Cardiovascular Risks in Overweight PCOS	NCT03151005
775	Grossman et al. / Safety Study of Allogeneic Mesenchymal Precursor Cell Infusion in Myocardial Infarction (AMICI)	NCT01781390
776	Meurer et al. / Reach Out: Emergency Department-Initiated Hypertension Behavioral Intervention Connecting Multiple Health Systems (ReachOut ED)	NCT03422718
777	Abel et al. / Technology Coaching Intervention for Black Women With Hypertension	NCT03577990
778	Chinitz et al. / Renal Nerve Denervation in Patients With Hypertension and Paroxysmal and Persistent Atrial Fibrillation (Symplicity AF)	NCT02064764
779	Anker et al. / EMPagliflozin outcomE tRial in Patients With chrOnic heaRt Failure With Preserved Ejection Fraction (EMPEROR-Preserved)	NCT03057951
780	Adams et al. / Tolvaptan For Worsening Outpatient Heart Failure: Role of Copeptin In Identifying Responders (TROUPER)	NCT02476409
781	Danaietash et al. / A Research Study to Show the Effect of Aprocitentan in the Treatment of Difficult to Control (Resistant) High Blood Pressure (Hypertension) and Find Out More About Its Safety (PRECISION)	NCT03541174
782	COmparing arNi and Ace For Improving Erectile Dysfunction in mEN With reduCed Ejection Fraction Heart Failure (CONFIDENCE-HF)	NCT03917459
783	Voors et al. / A Study to Test the Effect of Empagliflozin in Patients Who Are in Hospital for Acute Heart Failure	NCT04157751
784	Luther et al. / Cardiovascular Effects of GLP-1 Receptor Activation	NCT03101930
785	Markman et al. / Magnetic Stimulation to Treat VT Storm (STAR-VT)	NCT04043312
786	Tran et al. / MOBILE Intervention in College Students With Elevated Blood Pressure (MOBILE)	NCT05956925
787	Couderc et al. / Video-based Detection of Atrial Fibrillation	NCT04267133
788	McElrath et al. / Comparing Nifedipine and Enalapril in Medical Resources Used in the Postpartum Period	NCT04236258

© 2025 Rivera FB et al. *JAMA Network Open*.

789	Montalescot et al. / Firibastat or Ramipril After Acute Myocardial Infarction for Prevention of Left Ventricular Dysfunction (QUORUM)	NCT03715998
790	Felker et al. / Daxor - Blood Volume Analysis	NCT04111185
791	Study of the Safety of BMS-986259 in Participants With Post-Acute Decompensated Heart Failure	NCT04318093
792	Brubaker et al. / Exercise Intolerance in Elderly Patients With HFpEF(Heart Failure With Preserved Ejection Fraction) (SECRET-II)	NCT02636439
793	Upadhyay et al. / Tragus Stimulation to Prevent Atrial Fibrillation After Cardiac Surgery (TraP-AF)	NCT03392649
794	Freedland et al. / Stepped Care for Depression in Heart Failure (DASH-2)	NCT02997865
795	Nassif et al. / Dapagliflozin in PRESERVED Ejection Fraction Heart Failure (PRESERVED-HF)	NCT03030235
796	Franchi et al. / PD and PK Profiles of Switching Between Cangrelor and Ticagrelor Following Ticagrelor Pre-treatment (SWAP-5)	NCT04634162
797	Dhande et al. / Cardiac Resynchronization in the Elderly	NCT03031847
798	Haq et al. / Adaptive CRT Effect on Electrical Dyssynchrony (aCRT-ELSYNC)	NCT02543281
799	Victoria-Castro et al. / Evaluating Efficacy of Digital Health Technology in the Treatment of Congestive Heart Failure	NCT04394754
800	Ding et al. / Pulsewatch: Smartwatch Monitoring for Atrial Fibrillation After Stroke	NCT03761394
801	Verma et al. / Esophageal Temperature Management During Cryo AF Ablation (EnsoETM) (EnsoETM)	NCT04079634
802	Tardif et al. / Effect of Dalcetrapib vs Placebo on CV Risk in a Genetically Defined Population With a Recent ACS (dal-GenE)	NCT02525939
803	Taveira et al. / Group Medical Visits in Heart Failure (MEDIC-HF)	NCT02481921
804	Piccini et al. / Study to Gather Information About the Proper Dosing of the Oral FXIa Inhibitor BAY 2433334 and to Compare the Safety of the Study Drug to Apixaban, a Non-vitamin K Oral Anticoagulant (NOAC) in Patients With Irregular Heartbeat (Atrial Fibrillation) That Can Lead to Heart-related Complications. (PACIFIC-AF)	NCT04218266
805	Sharrief et al. / Stroke Telemedicine Outpatient Prevention Program for Blood Pressure Reduction (STOP-Stroke)	NCT03923790
806	Pletcher et al. / The PCORnet Blood Pressure Home Monitoring Study (BP Home)	NCT03796689
807	Gharacholou et al. / The Randomized OPTIMAL-ACT Trial	NCT03772613
808	IsHak et al. / Personalized Treatments for Depressive Symptoms in Patients With Advanced Heart Failure	NCT03688100
809	Parmana et al. / Role of Glutamine as Myocardial Protector in Elective On-Pump CABG Surgery With Low EF	NCT04560309
810	Cook et al. / Gut Butyrate and Blood Pressure in African Americans	NCT04415333
811	Samsonov et al. / Study to Evaluate the Effect on Parameters of Systemic Inflammation and Disease Outcomes and Safety of RPH-104 in Subjects With Acute ST-elevation Myocardial Infarction	NCT04463251
812	Jøns et al. / BIO monitorinG in Patients With Preserved Left ventricUlar Function AfteR Diagnosed Myocardial Infarction (BIO-GUARD-MI)	NCT02341534
813	Lewis et al. / Study to Assess the Effect of Omecamtiv Mecarbil on Exercise Capacity in Subjects With Heart Failure (METEORIC-HF)	NCT03759392
814	Spertus et al. / A Study on Impact of Canagliflozin on Health Status, Quality of Life, and Functional Status in Heart Failure (CHIEF-HF)	NCT04252287
815	Rosenson et al. / The Effects of Evolocumab in Patients With Diabetes and Atherosclerotic Vascular Disease	NCT03829046
816	Crews et al. / Five, Plus Nuts and Beans for Kidneys	NCT03299816

© 2025 Rivera FB et al. *JAMA Network Open*.

817	Campos et al. / SMS System for Patients With Uncontrolled Hypertension	NCT03596242
818	Kariuki et al. / Active You: A Novel Exercise Program for African Americans	NCT04280783
819	Postpartum Weight Loss for Women at Elevated Cardiovascular Risk (EMPOWER- Mom)	NCT04914819
820	Matlock et al. / A Multicenter Trial of a Shared DECision Support Intervention for Patients Offered Implantable Cardioverter-DEfibrillators	NCT03374891
821	Trinity et al. / Understanding the Exercise-Hypertension Paradox	NCT02034422
822	A Study to Measure Stomach Emptying in Overweight Non-diabetic and Diabetic Participants Using Tirzepatide	NCT04407234
823	Edelman et al. / Effectiveness of Orally Dosed Emergency Contraception in Obese Women - LNG (EC-Obesity)	NCT02863445
824	Garvey et al. / Two-year Research Study Investigating How Well Semaglutide Works in People Suffering From Overweight or Obesity (STEP 5)	NCT03693430
825	Ultrasonography Versus Palpation for Spinal Anesthesia in Obese Parturients Undergoing Cesarean Delivery	NCT03792191
826	Elkind-Hirsch et al. / Liraglutide 3mg (Saxenda) on Weight, Body Composition, Hormonal and Metabolic Parameters in Obese Women With PCOS (SAXAPCOS)	NCT03480022
827	Shariat et al. / Efficacy Of Quadratus Lumborum II Block For Laparoscopic Sleeve Gastrectomy	NCT04073056
828	Papamargaritis et al. / Saxenda in Obesity Services (STRIVE Study) (STRIVE)	NCT03036800
829	Lofton et al. / Clinical Efficacy and Safety of Using 3.0mg Liraglutide to Treat Weight Regain After Roux-en-Y Gastric Bypass Surgery	NCT03048578
830	Hebert et al. / Financial vs. Non-Financial Rewards for Weight Loss and Weight Maintenance	NCT02957539
831	Kauffman et al. / Computer-Delivered Intervention for Individuals With Obesity and Elevated Anxiety Sensitivity	NCT03917901
832	Ptomey et al. / Research Study to Investigate How Well Semaglutide Works Compared to Liraglutide in People Living With Overweight or Obesity (STEP 8)	NCT04074161
833	Corbin et al. / ACT1ON Phase 2 (SMART Pilot) and Phase 3 (Efficacy Trial Development) (ACT1ON DP3)	NCT03651622
834	Hoerster et al. / DVD Lifestyle Intervention (D-ELITE)	NCT03260140
835	Gorin et al. / Physical Activity Choices Everyday (PACE)	NCT03824769
836	Rebello et al. / Potato Research for Enhancing Metabolic Outcomes (PREMO)	NCT04203238
837	Odibo et al. / 17OHP-C Dosing Among Obese Pregnant Women	NCT03433040
838	Pierce et al. / Antibiotic Prophylaxis to Prevent Obesity-Related Induction Complications in Nulliparae at Term (APPOINT)	NCT03801252
839	Silver et al. / Cardiovascular Effects of GLP-1 Receptor Activation	NCT03101930
840	Luu et al. / Dysregulation of FSH in Obesity: Functional and Statistical Analysis	NCT02478775
841	Grilo et al. / Behavioral and Pharmacologic Treatment of Binge Eating and Obesity:<br>Acute Treatment	NCT03045341
842	Pearl et al. / Behavioral Weight Loss and Stigma Reduction	NCT03704064
843	Dixon et al. / Trial of Roflumilast in Asthma Management (TRIM) (TRIM)	NCT03532490
844	Cáceres et al. / Inhibitory Control Adult Weight Management	NCT04747886
845	McGee et al. / Effects of Exercise Training Intensity on Fitness and Insulin Sensitivity in African Americans (HI-PACE)	NCT02892331
846	Griebeler et al. / Comparing a Virtual vs. Face to Face Weight Management Program Using Phentermine for Patients With Overweight or Obesity	NCT04614545

© 2025 Rivera FB et al. *JAMA Network Open*.

847	Wisnivesky et al. / Asthma and Obesity: Pilot Study	NCT04113746
848	Wang et al. / Pilot Study of the Effect of Liraglutide 3.0 mg on Weight Loss and Gastric Functions in Obesity	NCT03523273
849	Saxena et al. / A 12-WEEK TITRATE STUDY TO EVALUATE SAFETY, TOLERABILITY AND PHARMACODYNAMICS OF PF-06882961 IN ADULTS WITH TYPE 2 DIABETES MELLITUS AND IN NON-DIABETIC ADULTS WITH OBESITY	NCT04617275
850	Onslow et al. / Sleep Disordered Breathing, Obesity and Pregnancy Study (SOAP) (SOAP)	NCT02086448
851	Tregellas et al. / Nicotinic Agonist Effects on BMI and Neuronal Response	NCT02458313
852	A Digital Flu Intervention for People With Cardiovascular Conditions (CardioFlu)	NCT04584645
853	Ellison et al. / Role of Sugammadex as Reversal Agent in Patients Extubated Immediately After Isolated Coronary Artery Bypass Grafting Surgery	NCT03939923
854	Gruß et al. / SPREAD-NET: PRactices Enabling Adapting and Disseminating in the Safety NET	NCT02325531
855	O'Donoghue et al. / Olpasiran Trials of Cardiovascular Events And LipoproteiN(a) Reduction - DOSE Finding Study	NCT04270760
856	Chen et al. / A New Operation for the Treatment for Long-standing Atrial Fibrillation	NCT03347695
857	Pereira et al. / Circulating NEP and NEP Inhibition Study in Heart Failure With Preserved Ejection Fraction	NCT03506412
858	Long-term Safety and Efficacy of Ralinepag in Pulmonary Arterial Hypertension	NCT02279745
859	Zhou et al. / Qubic Stim Cardiac Stimulator in China	NCT04390841
860	Tsay et al. / Atrial Fibrillation Algorithms Clinical Validation Study	NCT04699812
861	Dukkipati et al. / Intramural Needle Ablation for the Treatment of Refractory Ventricular Arrhythmias	NCT03204981
862	Safety of Argatroban Infusion in Conduction Disturbances	NCT05740371
863	Bensimhon et al. / Furoscix Real-World Evaluation for Decreasing Hospital Admissions in Heart Failure	NCT03458325
864	Vijayaraman et al. / ECG Belt to Assess Electrical Synchronization	NCT04583709
865	Di Biase et al. / Evaluation of VISITAG SURPOINT™ Module With External Processing Unit (EPU)	NCT03624881
866	Deneke et al. / Master Study of the BIOMONITOR III and Incision and Insertion Tool (FIT OneStep)	NCT04025710
867	Reddy et al. / Feasibility Study of the FARAPULSE Endocardial Multi Ablation System in the Treatment of Persistent Atrial Fibrillation	NCT04170621
868	Parrish et al. / Nashville - Hypertension Management Model	NCT04232124
869	Grünig et al. / An Open-Label, Long-Term Study of Oral Treprostinil in Subjects With Pulmonary Arterial Hypertension	NCT01560637
870	Galiè et al. /ACT-293987 in Pulmonary Arterial Hypertension	NCT01112306
871	Wali et al. / mHealth for Self-care of Heart Failure in Uganda	NCT04426630
872	Rubin et al. / RoBotic TCD Ultrasound BubbLe Study Compared to Transthoracic Echocardiography for Detection of Right to Left Shunt	NCT04604015
873	Shah et al. / The CASCADE HF Soft Launch and Calibration Phase I and II	NCT04738279
874	Vijgen et al. / A Study Assessing Arrhythmia Mapping With the Multi-Electrode OPTRELL™ Mapping Catheter	NCT04983797
875	Macle et al. / Performance of QDOT Micro™ Catheter With nGEN Generator for Patients With AFIB.	NCT04545619
876	Wang et al. / Mobile Technology for Blood Pressure Management	NCT04841317

© 2025 Rivera FB et al. *JAMA Network Open*.

877	Ray et al. / An Extension Trial of Inclisiran in Participants With Cardiovascular Disease and High Cholesterol	NCT03060577
878	Waxman et al. / A Study of Sotatercept for the Treatment of Pulmonary Arterial Hypertension	NCT03738150
879	Al-Shaer et al. / Bioavailability of SPMs in Obese Humans	NCT04701138
880	A Drug-Drug Interaction Study Between PF-06882961 and PF-06865571 in Healthy Adult Participants and Overweight Adults or Adults With Obesity Who Are Otherwise Healthy	NCT04839393
881	Flack et al. / Behavioral and Pharmacologic Treatment of Binge Eating and Obesity: Specialist Treatment	NCT04651218
882	Nelson et al. / Veteran Peer Coaches Optimizing and Advancing Cardiac Health (Vet- COACH)	NCT02697422
883	Rickard et al. / ECG Belt for CRT Response	NCT03504020
884	Hoppe et al. / Young Adult Hypertension Self-Management Clinical Trial (MyHEART)	NCT03158051
885	Sawyer et al. / Pregnancy-Related Hypertension: Adherence to a New Type of Monitoring (PHANTOM)	NCT04823949
886	Angiolillo et al. / Switching From DAPT to Dual Pathway Inhibition With Low-dose Rivaroxaban in Adjunct to Aspirin in Patients With Coronary Artery Disease (SWAP- AC)	NCT04006288
887	Bublitz et al. / Prenatal Mindfulness & Hypertension Study (HTN)	NCT03679117
888	Kapadia et al. / PROTECTED TAVR: Stroke PROTECTion With SEntinel During Transcatheter Aortic Valve Replacement (PROTECTED TAVR)	NCT04149535
889	Marcus et al. / Wire Instrumentation Using Radiofrequency Energy to Impact Transseptal Efficiency (WIRE-IT)	NCT04645342
890	Barr et al. / The Impact of Sharing Audio Recorded Clinic Visits on Self-management in Older Adults	NCT04344301
891	Pivotal Bioequivalence Study to Qualify Manufacturing Site Transfer for Prazosin Hydrochloride Capsules	NCT04967443
892	Shoamanesh et al. / Study to Gather Information About Proper Dosing and Safety of the Oral FXIa Inhibitor BAY 2433334 in Patients Following a Recent Non Cardioembolic Ischemic Stroke Which Occurs When a Blood Clot Has Formed Somewhere in the Human Body (But Not in the Heart) Travelled to the Brain.<br>(PACIFIC-STROKE)	NCT04304508
893	Rao et al. / Study to Gather Information About the Proper Dosing and Safety of the Oral FXIa Inhibitor BAY 2433334 in Patients Following an Acute Heart Attack (PACIFIC-AMI)	NCT04304534
894	Cooper et al. / Reducing Inequities in Care of Hypertension, Lifestyle Improvement for Everyone (RICH LIFE Project) (RICH LIFE)	NCT02674464
895	Vazquez et al. / Improving Chronic Disease Management With Pieces (ICD-Pieces)	NCT02587936
896	Umpierrez et al. / Dapagliflozin at Discharge on Hospital Heart Failure Readmission	NCT04249778
897	Humbert et al. / A Study of Sotatercept for the Treatment of Pulmonary Arterial Hypertension (PAH) (PULSAR)	NCT03496207
898	The Effects of Sacubitril/Valsartan on Cardiac Oxygen Consumption and Efficiency of Cardiac Work in Heart Failure Patients (TurkuPET)	NCT03300427
899	Piccini et al. / Botulinum Toxin Type A (AGN-151607) for the Prevention of Post-operative Atrial Fibrillation in Adult Participants Undergoing Open-chest Cardiac Surgery (NOVA)	NCT03779841
900	Solomon et al. / Dapagliflozin Evaluation to Improve the LIVEs of Patients With PReserved Ejection Fraction Heart Failure. (DELIVER)	NCT03619213
901	Melamed et al. / Effects of Vitamin D and Fish Oil on the Kidney in	NCT02757872

© 2025 Rivera FB et al. *JAMA Network Open*.

	Hypertensives	
902	Sharma et al. / A Study on BMS-986177 for the Prevention of a Stroke in Patients Receiving Aspirin and Clopidogrel (AXIOMATIC-SSP)	NCT03766581
903	Tita et al. / Chronic Hypertension and Pregnancy (CHAP) Project (CHAP)	NCT02299414
904	de Bruin et al. / Helping Hypertension Patients to Interpret Blood Pressure Readings and Motivate Blood Pressure Control	NCT04485637
905	Stone et al. / Absorb IV Randomized Controlled Trial	NCT02173379
906	Borlaug et al. / Inorganic Nitrite to Amplify the Benefits and Tolerability of Exercise<br>Training in Heart Failure With Preserved Ejection Fraction (HFpEF) (INABLE-Training) (INABLE)	NCT02713126
907	Yamashita et al. / Study of Efficacy and Safety of Inclisiran in Japanese Participants With High Cardiovascular Risk and Elevated LDL-C (ORION-15)	NCT04666298
908	Konstam et al. / Avoiding Treatment in the Hospital With Furoscix for the Management of Congestion in Heart Failure - A Pilot Study (AT HOME-HF)	NCT04593823
909	Emeruwa et al. / Lasix for the Prevention of De Novo Postpartum Hypertension (LAPP)	NCT04752475
910	Maykin et al. / Furosemide vs. Placebo for Severe Antepartum Hypertension	NCT04615624
911	Friedman et al. / Extravascular ICD Pivotal Study (EV ICD)	NCT04060680
912	Kitzman et al. / Verinurad Plus Allopurinol for Heart Failure With Preserved Ejection Fraction: The AMETHYST Randomized Clinical Trial	NCT04327024
913	von Lewinsky et al. / Impact of EMpagliflozin on Cardiac Function and Biomarkers of Heart Failure in Patients With Acute MYocardial Infarction (EMMY)	NCT03087773
914	Reddy et al. / Efficacy Study of Pacemakers to Treat Slow Heart Rate in Patients With Heart Failure (RAPID-HF)	NCT02145351
915	Wang et al. / Neonatal Sleep Intervention to Improve Postpartum Hypertension	NCT04864249
916	Efficacy and Safety of LCZ696 Compared to Valsartan on Cognitive Function in Patients With Chronic Heart Failure and Preserved Ejection Fraction (PERSPECTIVE)	NCT02884206
917	Douglas et al. / The PRECISE Protocol: Prospective Randomized Trial of the Optimal Evaluation of Cardiac Symptoms and Revascularization (PRECISE)	NCT03702244
918	Tranquart et al. / A Study of Flurpiridaz (18F) Injection for PET Imaging for Assessment of MPI Quality Using HPLC and SPE Manufacturing Processes	NCT04594941
919	Ferdinand et al. / Text My Hypertension BP Meds NOLA	NCT05074173
920	Meyer et al. / Cilostazol for HFpEF	NCT05126836
921	Freeman et al. / A Study of CIN-107 in Adults With Treatment-Resistant Hypertension (rHTN) (BrigHTN)	NCT04519658
922	Triebwasser et al. / Nudge to Drive Transitions of Care (REMIND)	NCT04660032
923	De Pooter et al. / Automated Assessment of PVI Using a Novel EP Recording System (PVISION)	NCT05043883
924	Mistry et al. / Blood Pressure After Endovascular Stroke Therapy-II (BEST-II)	NCT04116112
925	Kawut et al. / Pulmonary Hypertension and Anastrozole Trial (PHANTOM)	NCT03229499
926	Chirinos et al. / Effect of KNO3 Compared to KCl on Oxygen UpTake in Heart Failure With Preserved Ejection Fraction (KNO3CK OUT HFPEF)	NCT02840799
927	Haff et al. / Personalizing Intervention to Reduce Clinical Inertia in the Treatment of Hypertension	NCT04603560
928	Hoeper et al. / A Study of Sotatercept for the Treatment of Pulmonary Arterial Hypertension (MK-7962-003/A011-11)(STELLAR)	NCT04576988
929	Fanaroff et al. / Intermittent Fasting Adherence and Self Tracking (iFAST)	NCT04836312

© 2025 Rivera FB et al. *JAMA Network Open*.

930	Testani et al. / Mechanism and Effects of Manipulating Chloride Homeostasis in Acute Heart Failure	NCT03446651
931	Laffin et al. / Aldosterone Synthase Inhibition With Lorundrostat for Uncontrolled Hypertension: The Target-HTN Randomized Clinical Trial	NCT05001945
932	A Study of CIN-107 in Patients With Uncontrolled Hypertension (HALO)	NCT05137002
933	Neilan et al. / STOP-CA (Statins TO Prevent the Cardiotoxicity From Anthracyclines)	NCT02943590
934	Beaty et al. / Comparison of Bolus Dosing of Methohexital and Propofol in Elective Direct Current Cardioversion	NCT04187196
935	Ishani et al. / Diuretic Comparison Project (DCP)	NCT02185417
936	Frantz et al. / GB002 in Adult Subjects With Pulmonary Arterial Hypertension (PAH)	NCT04456998
937	O'Connor et al. / Medication Adherence Clinical Decision Support (ADH-Wizard)	NCT03748420
938	Yang et al. / Role of On-site CT-derived FFR in the Management of Suspect CAD Patients (TARGET)	NCT03901326
939	Wilkoff et al. / AdaptResponse Clinical Trial	NCT02205359
940	Vongpatanasin et al. / Preventing Metabolic Side Effects of Thiazide Diuretics With KMgCitrate	NCT02665117
941	Nissen et al. / Evaluation of Major Cardiovascular Events in Participants With, or at<br>High Risk for, Cardiovascular Disease Who Are Statin Intolerant Treated With Bempedoic Acid (ETC-1002) or Placebo (CLEAR Outcomes)	NCT02993406
942	Wang et al. / Effects of SGLT-2 Inhibition on Myocardial Fibrosis and Inflammation as Assessed by Cardiac MRI in Patients With DM2	NCT03782259
943	Borlaug et al. / Dapagliflozin (DAPA) Effects in HFpEF	NCT04730947
944	Sarraj et al. / SELECT2: A Randomized Controlled Trial to Optimize Patient's Selection for Endovascular Treatment in Acute Ischemic Stroke (SELECT2)	NCT03876457
945	Kapadia et al. / WATCH-TAVR, WATCHMAN for Patients With Atrial Fibrillation Undergoing Transcatheter Aortic Valve Replacement	NCT03173534
946	Lewis et al. / CES1 Crossover Trial of Clopidogrel and Ticagrelor	NCT03161678
947	Willekes et al. / Prophylactic Maze to Prevent Atrial Fibrillation in Adult Cardiac Surgery (PREVENT-AF)	NCT03604432
948	Holst et al. / A Study Evaluating the Efficacy and Safety of AP30663 for Cardioversion in Participants With Atrial Fibrillation (AF)	NCT04571385
949	Morrow et al. / Changes in NT-proBNP, Safety, and Tolerability in HFpEF Patients With a WHF Event (HFpEF Decompensation) Who Have Been Stabilized and Initiated at the Time of or Within 30 Days Post-decompensation (PARAGLIDE-HF)<br>(PARAGLIDE-HF)	NCT03988634
950	Suture Closure AFtEr VEIN Access for Cardiac Procedures (SAFE-VEIN)<br>Trial (SAFE-VEIN)	NCT04632641
951	Santoro et al. / Reprometabolic Syndrome Mediates Subfertility in Obesity	NCT02653092
952	Edelman et al. / Effectiveness of Orally Dosed Emergency Contraception in Obese Women - UPA (UPA-Obesity)	NCT02859337
953	Kapogiannis et al. / Intermittent Calorie Restriction, Insulin Resistance, and Biomarkers of Brain Function	NCT02460783
954	Hechenbleikner et al. / Endoscopic vs. Suction Device Calibration in Sleeve Gastrectomy	NCT03939819
955	Burger et al. / Neurobehavioral Plasticity to Regular Sugar-Sweetened Beverage Intake: An fMRI Experiment	NCT03490734

© 2025 Rivera FB et al. *JAMA Network Open*.

956	Jastreboff et al. / A Study of Tirzepatide (LY3298176) in Participants With Obesity or Overweight (SURMOUNT-1)	NCT04184622
957	Miller et al. / Augmented Care at Worksite for Diabetes Prevention	NCT03382873
958	Houston et al. / Utilizing Protein During Weight Loss to Impact Physical Function (UPLIFT)	NCT03074643
959	Buskmiller et al. / Early Anatomy Scan for Evaluation of Obese Pregnant Women (EASE-O)	NCT04639973
960	Jastreboff et al. / A Study of LY3437943 in Participants Who Have Obesity or Are Overweight	NCT04881760
961	Plessow et al. / The Effects of Oxytocin in Obese Adults	NCT03043053
962	Acosta et al. / Individualized Obesity Pharmacotherapy	NCT03374956
963	A Study to Measure Energy Expenditure and Food Intake in Participants With Obesity Using Tirzepatide	NCT04081337
964	Aguirre et al. / Assessment of and Treatment Applied to Food Addiction in a Rural Healthy Behaviors Clinic	NCT03431831
965	Acosta et al. / Anti-Obesity Phentermine-Topiramate Extended Release Pharmacotherapy vs Placebo Among Patients Using a Wearable Activity Tracker	NCT04408586
966	Buckeridge et al. / Study of Multiple Oral Doses of PF-07081532 in Adult Participants With Type 2 Diabetes Mellitus	NCT05158244
967	Wharton et al. / A Study of LY3502970 in Participants With Obesity or Overweight With Weight-related Comorbidities	NCT05051579
968	le Roux et al. / A Study to Test Whether Different Doses of BI 456906 Help People With Overweight or Obesity to Lose Weight	NCT04667377
969	Frey et al. / A Pilot Study: High Versus Low Oxytocin Dosing for Induction of Labor in Pregnant Patients With Obesity (HILIO-PILOT)	NCT05289869
970	Vaughn et al. / Evaluation of an Enhanced Delivery Model for Go NAPSACC	NCT03938103
971	McElroy et al. / Saxenda® in Obese or Overweight Patients With Stable Bipolar Disorder (Investigator Initiated)	NCT03158805
972	Rosenberg et al. / Healthy Aging Resources to Thrive (HART) (HART)	NCT03739762
973	Zimmerman et al. / Impact of Sleep Restriction on Performance in Adults	NCT02960776
974	A Study of Tirzepatide in Overweight and Very Overweight Participants	NCT04311411
975	Zhao et al. / A Study of Tirzepatide (LY3298176) in Chinese Participants Without Type 2 Diabetes Who Have Obesity or Overweight (SURMOUNT-CN) (SURMOUNT-CN)	NCT05024032
976	Cherrington et al. / Preventing Diabetes in the Deep South: Extending Partnerships and Adapting Interventions to Reach Rural Communities at High Risk.	NCT04343872
977	Zampieri et al. / A Study to Evaluate Mavacamten in Adults With Symptomatic Obstructive HCM Who Are Eligible for Septal Reduction Therapy	NCT04349072
978	Shake et al. / Bingocize: A Novel Mobile Application for Older Adult Health	NCT03629912
979	Brewer et al. / The FAITH! Trial: A mHealth Intervention to Improve Cardiovascular Health Among African-Americans	NCT03777709
980	Longenecker et al. / A Nurse-led Intervention to Extend the HIV Treatment Cascade for Cardiovascular Disease Prevention	NCT03643705
981	Lincoff et al. / A Study to Evaluate the Effect of Testosterone Replacement Therapy (TRT) on the Incidence of Major Adverse Cardiovascular Events (MACE) and Efficacy Measures in Hypogonadal Men	NCT03518034
982	Reynolds et al. / Nuts and Oil Pilot Study	NCT04361617
983	Chinitz et al. / Accelerometer Sensing for Micra AV Study	NCT04245345
984	Weber et al. / Worksite Lifestyle Program for Reducing Diabetes and Cardiovascular Risk in India	NCT02813668

© 2025 Rivera FB et al. *JAMA Network Open*.

985	Crijns et al. / INhalation of Flecainide to Convert Recent Onset SympTomatic Atrial Fibrillation to siNus rhyThm (INSTANT)	NCT03539302
986	Wu et al. / Study Evaluating the Pharmacokinetics of Mavacamten in Healthy Adult Chinese Subjects	NCT05135871
987	Kepper et al. / PREVENT Tool Study: Late Effects Clinic	NCT04530825
988	Osorio et al. / Evaluation of QDOT MICRO™ Catheter for Pulmonary Vein Isolation in Subjects With Paroxysmal Atrial Fibrillation	NCT03775512
989	Viera et al. / Tailored to You (TTY) Pilot Study	NCT05141175
990	O'Donoghue et al. / Further Cardiovascular Outcomes Research With PCSK9 Inhibition in Subjects With Elevated Risk Open-label Extension	NCT02867813
991	McGuire et al. / Clinical Study to Assess the Safety, Tolerability and Efficacy of Macitentan in Subjects With Inoperable Chronic Thromboembolic Pulmonary Hypertension	NCT02060721
992	Faro et al. / Study of Clinic-delivered Physical Activity Referrals to Cancer Survivors	NCT05216380
993	Knops et al. / Acute Feasibility Investigation of a New S-ICD Electrode	NCT03802110
994	Maddahi et al. / An International Study to Evaluate Diagnostic Efficacy of Flurpiridaz (18F) Injection PET MPI in the Detection of Coronary Artery Disease (CAD)	NCT03354273
995	Miller et al. / EXPEDITE: A Study of Remodulin Induction Followed by Orenitram Optimization to Treat Pulmonary Arterial Hypertension	NCT03497689
996	AcQBlate Force Sensing Ablation System US IDE for Atrial Flutter (AcQForce Flutter)	NCT04658940
997	Ortega-Paz et al. / Impact of Chronic Kidney Disease on Clopidogrel Effects in Diabetes Mellitus	NCT03774394
998	Stevenson et al. / Intramural Needle Ablation for Ablation of Recurrent Ventricular Tachycardia	NCT01791543
999	Gupta et al. / Perclose Multi-Access Duplex Ultrasound (DUS) Study	NCT04904809
1000	De Groot et al. / Safety and Feasibility of Atrial Deganglionation as Adjunctive Therapy for AF	NCT04775264
1001	Nair et al. / TactiFlex Paroxysmal Atrial Fibrillation IDE Trial	NCT04356040
1002	Clinical Validation Study for CPM Device	NCT05445206
1003	Zareba et al. / Pilot Randomized Trial With Flecainide in ARVC Patients	NCT03685149
1004	Respiration Validation for CPM Device	NCT05445492
1005	PAVS in Cardiology	NCT04656132
1006	Park et al. / Abbott Next Generation Drug Eluting Stent 48mm Study	NCT04282148
1007	Kini et al. / Effect of Evolocumab on Coronary Plaque Characteristics	NCT04710368
1008	Bowdle et al. / Sugammadex Titration in Cardiac Surgery Patients	NCT05246397
1009	Ellenbogen et al. / Boston Scientific's Cryoballoon in the Treatment of Symptomatic Drug Refractory Paroxysmal Atrial Fibrillation	NCT04133168
1010	Nash et al. / A Study to Evaluate BMS-986141 Added on to Aspirin or Ticagrelor or the Combination, on Thrombus Formation in a Thrombosis Chamber Model in Participants With Stable Coronary Artery Disease and Healthy Participants	NCT05093790
1011	Verma et al. / Pulsed Field Ablation to Irreversibly Electroporate Tissue and Treat AF	NCT04198701
1012	Feasibility Study of the FARAPULSE™ Cardiac Ablation System Plus in the Treatment of Persistent Atrial Fibrillation(PersAFOne II)	NCT05152966
1013	Shlofmitz et al. / A Study to EXhibit Percutaneous Coronary Artery Dilatation With Non-Slip Element Balloon	NCT04985773

1014	Sharp et al. / SPYRAL DYSTAL Renal Denervation Global Clinical Study	NCT04311086
1015	Argente et al. / Setmelanotide Phase 2 Treatment Trial in Participants With Rare Genetic Disorders of Obesity	NCT03013543
1016	Padilla et al. / Effects of Walking and Heating on Vascular Function in Diabetic Patients	NCT03203694
1017	Bandera et al. / TOPS for African American Breast Cancer Survivors	NCT04741802
1018	Darcey et al. / Brain Dopamine Function in Human Obesity	NCT03648892
1019	Olson et al. / Targeting Body Image Among Women of Higher Body Weight	NCT04810247
1020	Rogers et al. / Endoscopic Surgery for Bariatric Revision After Weight Loss Failure	NCT01871896
1021	Weight Regain Treatment Post-Bariatric Surgery	NCT04662801
1022	Study in Primary Care Evaluating Inclisiran Delivery Implementation + Enhanced Support (SPIRIT)	NCT04807400
1023	Risbano et al. / Oral Nitrite in Patients With Pulmonary Hypertension and Heart Failure With Preserved Ejection Fraction (PH-HFpEF)	NCT03015402
1024	Franchi et al. / Pharmacodynamic and Pharmacokinetic of Switching From Cangrelor to Prasugrel in ACS Patients Undergoing PCI (SWAP-6)	NCT04668144
1025	Mentz et al. / Randomized Placebo-controlled Trial of FCM as Treatment for Heart Failure With Iron Deficiency / and Sub-Study (HEART-FID)	NCT03037931
1026	Hennessey et al. / Dynamic Coronary Roadmap for Contrast Reduction (DCR4Contrast)	NCT04085614
1027	Ali et al. / ILUMIEN IV: OPTIMAL Percutaneous Coronary Intervention (PCI)	NCT03507777
1028	Bennett et al. / Nourish: A Digital Health Program to Promote the DASH Eating Plan Among Adults With High Blood Pressure	NCT03875768
1029	Rifkin et al. / Self-management of Blood Pressure Medication for Hypertensive Veterans	NCT03224624
1030	Sharma et al. / Rotational Atherectomy Combined With Cutting Balloon to Optimize Stent Expansion in Calcified Lesions (ROTA-CUT)	NCT04865588
1031	Amro et al. / Effectiveness of Higher Aspirin Dosing for Prevention of Preeclampsia in High Risk Obese Gravida (ASPREO)	NCT03961360
1032	Magnani et al. / Atrial Fibrillation Health Literacy and Information Technology Trial (AFibLITT)	NCT04075994
1033	Kosiborod et al. / Research Study to Investigate How Well Semaglutide Works in People Living With Heart Failure and Obesity (STEP-HFpEF)	NCT04788511
1034	Zile et al. / Regression of Fibrosis & Reversal of Diastolic Dysfunction in HFpEF Patients Treated With Allogeneic CDCs (RegressHFpEF)	NCT02941705
1035	Ter Maaten et al. / Pragmatic Urinary Sodium-based Treatment algoritHm in Acute Heart Failure (PUSH-AHF)	NCT04606927
1036	Vijayaraman et al. / HIS-Purkinje Conduction System Pacing Optimized Trial of Cardiac Resynchronization Therapy (HOT-CRT)	NCT04561778
1037	Cox et al. / Efficacy and Safety of Dapagliflozin in Acute Heart Failure (DICTATE- AHF)	NCT04298229
1038	Mankowski et al. / Nicotinamide Riboside as an Enhancer of Exercise Therapy in Hypertensive Older Adults (The NEET Trial)	NCT04112043
1039	Magnani et al. / Atrial Fibrillation Health Literacy and Information Technology Trial in Rural Pennsylvania Counties (AFibLITT_R)	NCT04076020
1040	Carson et al. / Myocardial Ischemia and Transfusion (MINT)	NCT02981407
1041	Lyons et al. / Taking Care of Us: A Dyadic Intervention for Heart Failure Couples (TCU)	NCT04737759
1042	Lincoff et al. / Semaglutide Effects on Heart Disease and Stroke in Patients With Overweight or Obesity (SELECT)	NCT03574597

© 2025 Rivera FB et al. *JAMA Network Open*.

1043	Elewa et al. / Ablation Targets of Scar-related Ventricular Tachycardia Identified by Dynamic Functional Substrate Mapping	NCT05086510
1044	Kutyifa et al. / Multicenter Automatic Defibrillator Implantation Trial With Subcutaneous Implantable Cardioverter Defibrillator (MADIT S-ICD)	NCT02787785
1045	Yan et al. / Treatment of Early Hypertension Among Persons Living With HIV in Haiti	NCT04692467
1046	Leary et al. / Repurposing a Histamine Antagonist to Benefit Patients With Pulmonary Hypertension (REHAB-PH)	NCT03554291
1047	Eichen et al. / Novel Executive Function Training for Obesity (NEXT)	NCT03724396
1048	Venditti et al. / Reducing Cardiometabolic Risk and Promoting Functional Health in Older Adults With Obesity and Prediabetes (Sustain-DPP)	NCT03500640
1049	Voils et al. / Using Partners to Enhance Long-Term Weight Loss (Partner2Lose)	NCT03801174
1050	A Study to Compare Two Different Forms of PF-07081532 in Adults Who Are Overweight or Obese	NCT05677867
1051	Leahey et al. / Peer Support for Weight Loss Maintenance	NCT03396653
1052	Garvey et al. / A Study of Tirzepatide (LY3298176) in Participants With Type 2 Diabetes Who Have Obesity or Are Overweight (SURMOUNT-2)	NCT04657003
1053	Bays et al. / A Study of VI-0521 on Ambulatory Blood Pressure (ABPM) in Overweight or Obese Subjects	NCT05215418
1054	Santos et al. / Healthy Mothers-Healthy Children Nutrition (HMHC)	NCT03866902
1055	Wadden et al. / A Study of Tirzepatide (LY3298176) In Participants After A Lifestyle Weight Loss Program (SURMOUNT-3)	NCT04657016
1056	Aronne et al. / A Study of Tirzepatide (LY3298176) in Participants With Obesity or Overweight for the Maintenance of Weight Loss (SURMOUNT-4)	NCT04660643
1057	Unick et al. / Phone Coaching and Internet-delivered Weight Loss	NCT03867981
1058	A Study of Tirzepatide (LY3298176) in Participants With Obesity Disease (SURMOUNT-J)	NCT04844918
1059	Saucedo et al. / Misoprostol Dosing in BMI Greater Than 30 (MD30 RCT)	NCT05262738
1060	Jacobs et al. / Modulation of the Intestinal Microbiome by a High Protein Diet (HPD)	NCT04812964
1061	Research Study Looking at How Well Semaglutide Works in People Suffering From Obesity and Knee Osteoarthritis	NCT05064735
1062	Davey et al. / Assess and Adapt to the Impact of COVID-19 on CVD Self-Management and Prevention Care in Adults Living With HIV (AAIM-High)	NCT04661813
1063	Plat et al. / Safety Study of Etripamil Nasal Spray for Patients With Paroxysmal Supraventricular Tachycardia. NODE-303	NCT04072835
1064	Berman et al. / Effect of Evolocumab on Coronary Atherosclerosis	NCT03689946
1065	Nakamura et al. / Absorb GT1 Japan PMS	NCT03409731
1066	Wright et al. / Trial to Assess the Effect of Long Term Dosing of Inclisiran in Subjects With High CV Risk and Elevated LDL-C	NCT03814187
1067	A Study Assessing Arrhythmia Mapping With a Globe-Shaped, High-Density, Multi- Electrode Mapping Catheter	NCT05373862
1068	Morris et al. / Comparison of Traditional, Web-based or a Combined Cardiac Rehabilitation Programme	NCT05326529
1069	Duytschaever et al. / A Study for Treatment of Paroxysmal Atrial Fibrillation (PAF) by Pulsed Field Ablation (PFA) System With Irreversible Electroporation (IRE)	NCT04524364
1070	Mahler et al. / EMERALD (Emergency Medicine Cardiovascular Risk Assessment for Lipid Disorders)	NCT05742841
1071	Gurewich et al. / Unmet Social Needs Study	NCT04977583

© 2025 Rivera FB et al. *JAMA Network Open*.

1072	Cortés et al. / Reducing Cardiovascular Disease Risk in Perimenopausal Latinas	NCT04313751
1073	Guldan et al. / Comparison of Propofol and Sevoflurane as a Primary Anesthetic for Cardiac Ablation of Atrial Fibrillation.	NCT02697448
1074	Duffy et al. / Feasibility Study of a Novel mHealth Application to Enable Community Health Workers to Manage	NCT05479097
1075	Study of Efficacy of Oral Sacubitril/Valsartan in Adult Patients With Non-obstructive Hypertrophic Cardiomyopathy	NCT04164732
1076	Henriksen et al. / LiquID Guide Catheter Extension Safety Study	NCT05406596
1077	McVay et al. / Preventing Weight Gain Among Those Who Decline Behavioral Weight Loss Treatment (STEADY)	NCT04751656
1078	Melanson et al. / Brown Adipose Tissue Activity in Pre- and Postmenopausal Women	NCT02927392
1079	Szymkiewicz et al. / Benefits of μCor in Ambulatory Decompensated Heart Failure (BMADHF)	NCT03476187

© 2025 Rivera FB et al. *JAMA Network Open*.
