# Supplementary material for: Participation of Women in Cardiovascular Trials From 2017 to 2023: A Systematic Review
Source: JAMA Netw Open. 2025 Aug 31;8(8):e2529104. doi: 10.1001/jamanetworkopen.2025.29104 (PMC12400126; doi:10.1001/jamanetworkopen.2025.29104)
Supplement: Supplement 2. — Data Sharing Statement [file jamanetwopen-e2529104-s002.pdf]

Data Sharing Statement

Rivera. Participation of Women in Cardiovascular Trials From 2017 to 2023. *JAMA Netw Open*. Published August 31, 2025. doi:10.1001/jamanetworkopen.2025.29104

Data
Data available: No
